# Bi-stability in type 2 diabetes mellitus multi-organ signalling network

**DOI:** 10.1371/journal.pone.0181536

**Published:** 2017-08-02

**Authors:** Shubhankar Kulkarni, Sakshi Sharda, Milind Watve

**Affiliations:** 1 Biology, Indian Institute of Science Education and Research, Pashan, Pune, Maharashtra, India; 2 Institute of Ecology and Evolution, University of Bern, Bern, Switzerland; University of Michigan, UNITED STATES

## Abstract

Type 2 diabetes mellitus (T2DM) is believed to be irreversible although no component of the pathophysiology is irreversible. We show here with a network model that the apparent irreversibility is contributed by the structure of the network of inter-organ signalling. A network model comprising all known inter-organ signals in T2DM showed bi-stability with one insulin sensitive and one insulin resistant attractor. The bi-stability was made robust by multiple positive feedback loops suggesting an evolved allostatic system rather than a homeostatic system. In the absence of the complete network, impaired insulin signalling alone failed to give a stable insulin resistant or hyperglycemic state. The model made a number of correlational predictions many of which were validated by empirical data. The current treatment practice targeting obesity, insulin resistance, beta cell function and normalization of plasma glucose failed to reverse T2DM in the model. However certain behavioural and neuro-endocrine interventions ensured a reversal. These results suggest novel prevention and treatment approaches which need to be tested empirically.

## Introduction

The classical thinking about the pathogenesis of Type 2 diabetes mellitus (T2DM) can be summarized in the form of five postulates: (i) Obesity results when net energy intake exceeds net energy expenditure. (ii) Obesity leads to insulin resistance. (iii) To compensate for the insulin resistance, more insulin is produced by the pancreatic β-cells. (iv) Chronically increased rate of insulin synthesis leads to ‘exhaustion’ or some form of dysfunction of β-cells which causes relative insulin insufficiency. This combination of insulin resistance and relative insulin insufficiency results in hyperglycaemia. (v) The pathophysiological complications of T2DM are a consequence of chronically elevated glucose levels in the blood [1,2].

A number of recent studies have exposed many gaps, flaws and paradoxes in this thinking [1–3]. The inability to cure diabetes can be attributed to these flaws and the clinical approach that uses this classical thinking in patient treatment. Since hyperglycemia was assumed to be the primary cause of the macrovascular and microvascular complications, treating hyperglycemia was the major course of treatment for T2DM patients. It was observed in many large scale clinical trials that normalizing blood glucose is not sufficient to avoid diabetic complications [4].

One of the fundamental paradoxes of T2DM is that the diabetic state is known to be irreversible although no component of the pathophysiology is individually irreversible. Beta cell loss was considered irreversible for some time but they are shown to have good regeneration capacity[5–8]. Therefore, the reason why T2DM cannot be cured is not known. Experiments in rodents and humans using different means to suppress insulin production have shown that whenever insulin production was suppressed, insulin sensitivity increased and blood sugar remained normal [9–16]. Such experiments have raised doubts whether insulin resistance and inadequate insulin production is necessary and sufficient for hyperglycemia in T2DM.

Although T2DM is historically identified as a condition of increased plasma glucose levels owing to inadequate insulin action, we know today that not only insulin and glucose but a large number of metabolites, hormones, growth factors, neurotransmitters, neuropeptides, cytokines, behaviours and neuronal signals are up or down-regulated in this disorder. Whether alterations in these signals are causes or consequences of altered insulin signalling and hyperglycemia is not clearly known [2]. We need to be open to the possibility that insulin and glucose are not central players but only two of the links in a complex network of signals. In order to get a good understanding of the pathophysiology of T2DM we need to consider all demonstrated interactions between molecules and other signals involved in T2DM without any prejudice and construct a comprehensive model.

We constructed a multi-organ multi-signal interactive network model of pathophysiology of T2DM and studied its behaviour. We show here that a network explains the pathophysiology of T2DM better than a simplistic insulin and glucose centred model. The model was validated by testing many of its predictions and the results demonstrated that most of the characteristics of T2DM are contributed by the structure of the network rather than impairment of insulin signalling alone. Since the classical drug targets for the treatment of T2DM failed to ensure a complete cure [17], a systematic search for alternative markers and targets is needed and a network model is likely to give some directions for the search. In the model, interventions that could reverse the insulin resistant state were not related to obesity, beta cell functionality, insulin production or insulin action but to a set of behavioural and neuro-endocrine targets.

## Materials and methods—The network model

### Identifying nodes and links of the network

**Fig 1:**
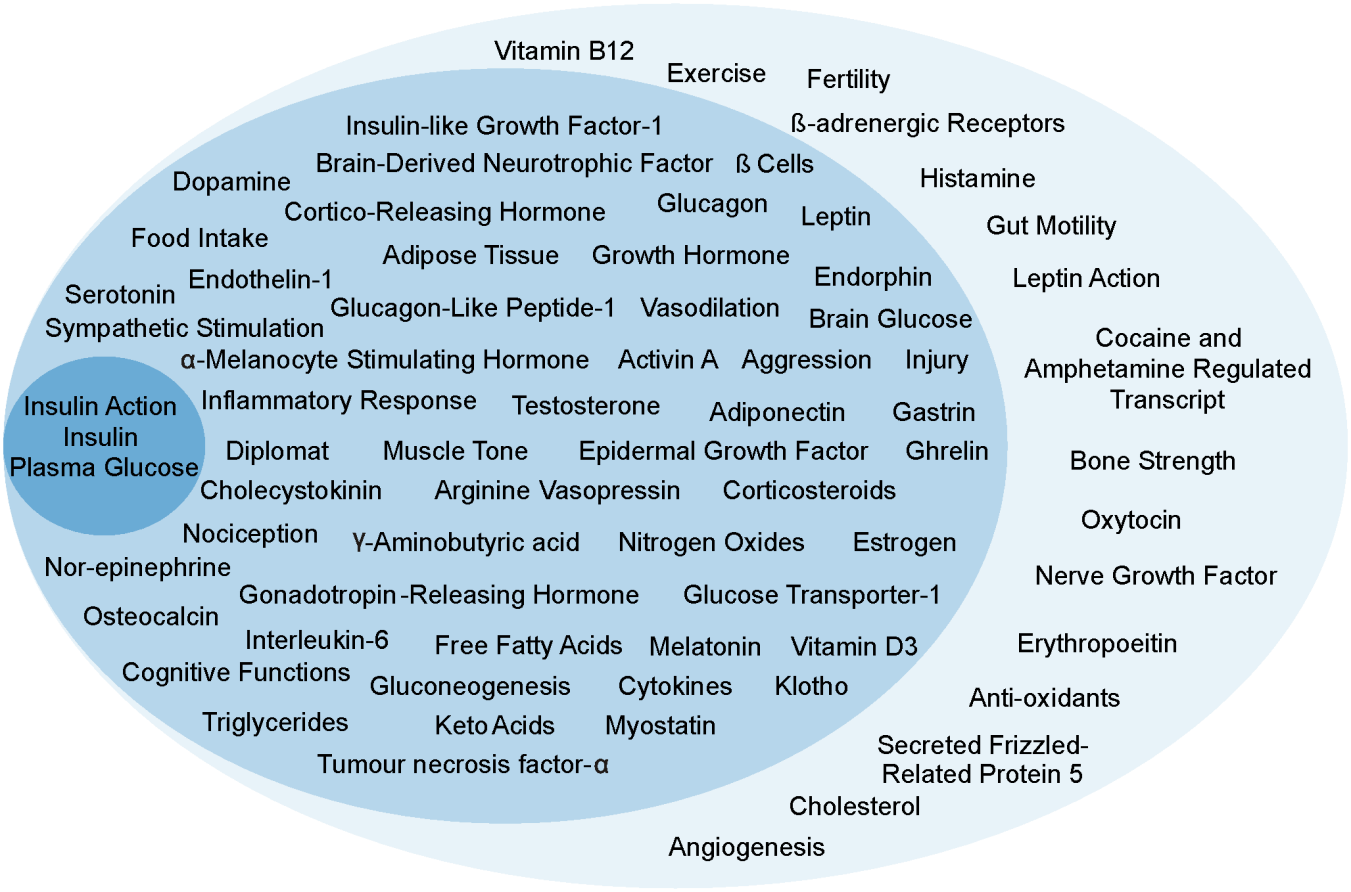
Signals in their respective tiers. First tier (innermost circle) includes players classically believed to be central to T2DM. Second tier (intermediate whorl) includes the players that directly affected or were directly affected by the players in the first tier. The third tier (outermost whorl) included players that affected those in the second tier or were affected by them.

We started with the classical theory of T2DM involving the 3 main variables classically believed to be central to T2DM namely plasma insulin level, insulin resistance and plasma glucose level. We searched literature for signals that affected one or more of the three (direct effectors) and further for signals that affected the direct effector signals (indirect effectors). Since specific behaviours are also known to trigger certain hormones and growth factors among the direct effectors, behaviours were also included in the list of signals. Thus, our definition of signals includes nutrients, metabolites, hormones, growth factors, cell populations, behaviours and neuronal signals (Fig 1). All our signals have a functional meaning. So, a down-regulation means loss or decrease in the signal. Whether it is because of structural change or any other change, is considered irrelevant.

The source data to extract possible interactions amongst the listed signals were publications reporting interventional studies giving causal evidence for a positive effect (up-regulation) or a negative effect (down-regulation) of a given signal on another signal of interest. All searches were made in ‘Google Scholar’ and ‘BioMedNet’ using the name(s) of the target nodes and “regulation of”, “expression of” and “affected by” as key words. Correlations and associations were not considered as evidence for an interaction. All published interactions were treated with equal weighting. No weighting of interactions was done by number of studies/ publications, validation, reliability, impact factor or level of current acceptance. Since, most of the interventional data comes from non-human species; we included all experiments with humans, rodents or other mammalian hosts (see S1 Table for model organisms used in the reference for each link).

After listing a large number of possible interactions, we applied the following inclusion and exclusion criteria and redundancy filters. Since our focus was on signalling between cell types and organs we excluded strictly intracellular pathways. If two or more signals shared the same upstream signal/s and the downstream effect/s, they were merged into one. From a known linear signalling pathway, only one molecule was listed. However, if there was a branching point in a pathway, it was listed as a signal. Only the signals having both upstream and downstream effects from other nodes of the network were included (see S1 Text for details).

**Fig 2:**
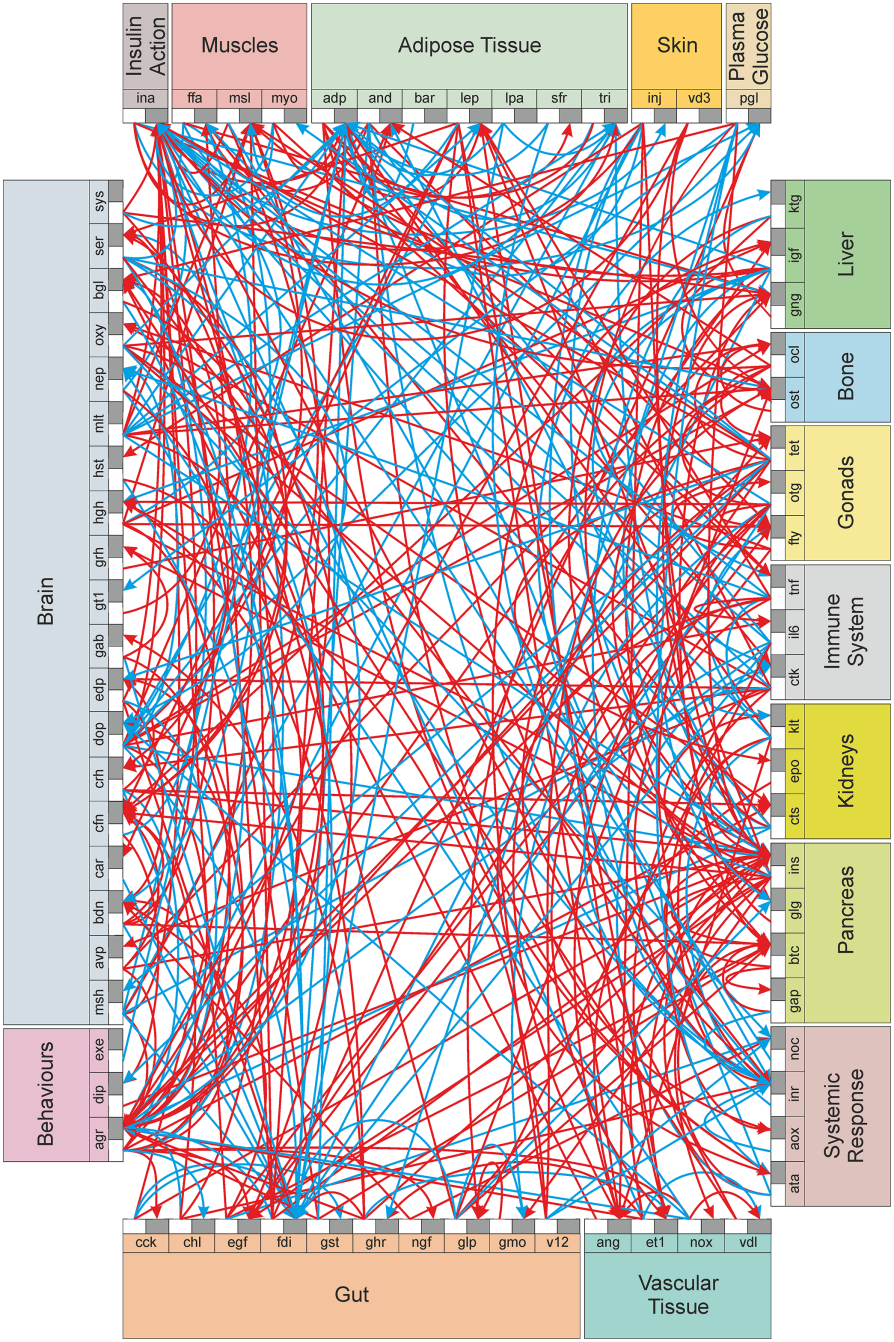
The inter-organ signalling network involved in the pathogenesis of T2DM. Each organ (coloured rectangles) displays the signals it produces. The outbound (white rectangle) and inbound (black rectangle) portals for each signal are shown. Red arrows indicate up-regulation interactions and cyan, down-regulation interactions. (See also S2 Table).

Finally, 330 interactions among 72 signals were identified from 491 publications and incorporated in the model (see S2 Table for details of the nodes and links with references). A network was constructed using these signals and interactions (Fig 2). All signals were treated as organ specific nodes and the interactions formed the directional links (in the network) between these nodes. If a given signal had different actions in different organs they were considered different nodes. For example, glucose in blood and that in the brain were treated as separate nodes. A limitation of the study is that the network model may be currently incomplete due to lack of specific studies (studies yet to be pursued by the scientific community), publication bias or studies that we may have missed during literature survey. Currently, the model is only qualitative, in that it considers normal, up-regulated and down-regulated states as discrete states. Each of the links may have some quantitative dynamics which may be linear or non-linear which was not incorporated in the current model.

### Perturbation simulations

A combination of Microsoft Excel 2007 for data input (addition of links to the network) and output (network perturbation results) and Visual Basic Application for executing the links was used to construct a network perturbation model. The signals were treated as nodes that can have one of three states namely 0 or baseline, +1 or up-regulated and -1 or down-regulated. Also, the directional links were of three different kinds namely up-regulatory or positive (which increased the state of the downstream node by 1), down-regulatory or negative (which decreased the state of the downstream node by 1) and basal level (which did not change the state of the downstream node). A zero signal here does not mean that there is no signal; it rather denotes that there is basal level signalling going on between the two nodes. Although the model considers only discrete states, it does not indicate extreme states. For example, -1 state of beta cell mass does not mean complete destruction of beta cells. In T2DM, a substantial proportion of beta cells survives lifelong [18]. Therefore, even in the -1 state of beta cells, insulin producing capacity is not assumed to be completely lost.

After constructing the network, we studied the effects of different kinds of perturbations in the network. At the beginning all nodes were at a default state of zero. Whenever a node was manually up or down-regulated, the state of that node changed to +1 or -1 respectively. All the directional links starting from that node were activated to change the states of the recipient nodes (first generation nodes). Subsequently directional links from these first generation nodes were activated to change the states of nodes further downstream (second generation nodes). The event of activation of one generation of nodes was termed as a ‘cycle’. Whenever a node received activated signals from more than one other node, the signals were added arithmetically to give a net signal strength. Based on the net positive or negative value of the signal strength, the state of the node was changed by +1 or -1 respectively; but without exceeding the state limits of -1 to +1. If the net signal strength was zero or normal in a given cycle, then the node returned to its normal default state. Thus at any given time the direction of change in the state of a node was solely determined by the net input signal. However, the step length for any change was restricted to unity, i.e. the state -1 could not become +1 in a single step.

Mathematically, the function of each node in every cycle can be explained as follows.

If *Si* ≠ 0, *then si* = Σ*eji*; where ‘S’ is the state of the node ‘i’, ‘s’ is the cumulative signal it received and ‘eji’ is the link from node j to i.

Depending upon the cumulative signal, the node is assigned a state.

If *si* > 0, *Si*(*t*) = *Si*(*t* − 1) + 1

If *si* = 0, *Si*(*t*) = 0

If *si* < 0, *Si*(*t*) = *Si*(*t* − 1)– 1; where ‘t’ is the cycle number

The state is then bound to limits -1 to +1

If *Si*(*t*) ≤ −1,*Si*(*t*) = −1

If *Si*(*t*) ≥ 1,*Si*(*t*) = 1

For example, to simulate the effects of primary hyperinsulinemia, the state of insulin in the starting cycle was made 1 where all the other nodes had a state of zero. In the first cycle, the direct effects of insulin were executed. Hence, only those nodes that were immediate downstream of insulin altered their state to +1 or -1 depending upon whether they received up-regulation or down-regulation link respectively, from the insulin node. In the current example, β-cells, leptin, klotho, EGF, cognitive functions, endothelin-1, gonadotropin—releasing hormone, nitric oxides and gut motility were up-regulated (state changed to +1); and keto acids and adiponectin were down-regulated (state changed to -1). In the second cycle, the immediate effects of these first generation nodes were executed. Thus in every cycle, the effects radiated, and because all the nodes lay in a network, in a few cycles, every node was affected in some way or the other. The recorded output was the state of each node after each cycle.

In the model above, the step length was always unity. As it changed from -1 to 0 in one cycle, the signals changed according to the new states and the next step was decided by the new signals. The step length was altered in two other variations of the model. One allowed a direct leap from -1 to +1 if the net signal was > 0 or vice versa. This variation of the model did not consider change in signals during state transition. In another variation the states as well as steps were fine grained with a resolution of 0.1 so that twenty different states for each node were possible between -1 and +1. Each link when activated led to a change of 0.1 in the downstream effector node. Multiple signals led to a cumulative signal strength which changed the state of the node quantitatively between the limits of -1 and +1. We examined whether the results were sensitive to the step length.

We used two types of perturbations separately or in combinations. (i) Point perturbations, i.e., after the perturbation was made in the starting cycle, the perturbed node came back to basal state after the first cycle; and then its state was allowed to be decided by the links it received eventually from other nodes. (ii) Sustained perturbations, i.e., the state of a starting perturbation node was changed and the changed state was maintained independent of any link it received subsequently.

A stable state of a node was described as a consistent resultant state of the node which remained so throughout further cycles. If a node changed its state with a repeated cyclic pattern of a fixed periodicity throughout the cycles, it was termed as a node in stable oscillation. If a node changed its states with unpredictably altering periodicity, it was termed as a node in a chaotic state. The stable state of the system was defined as a state in which every node was in a stable state or in short term deterministic oscillations. Further for the definition of a stable state it was necessary that if the system was point perturbed starting with that state it returned to the same state. If an apparently stable state obtained after one perturbation did not return to it after any other point perturbation it was called pseudo-stable state. A chaotic state of the system was defined by one or more nodes being in a chaotic state. Whenever there were stable oscillations or chaos the average of the last hundred cycles was taken as the ‘mean final state’ for a node.

### Some debatable links

A surprising finding of the search for links was that some of the classical beliefs were not supported by interventional evidence. For example we found no interventional evidence that muscle insulin resistance was compensated by hyperinsulinemia. Lack of evidence for this widely held assumption is acknowledged [2,19,20] but the assumption continues to be a part of mainstream thinking. Strictly going by the inclusion criteria of the model, we should not have included this link in the model. However since compensatory hyperinsulinemia is a widely held belief, we decided to run (make point perturbations to the network model and observe any changes in the Results) the model independently with and without this link. The difference in the outcomes of the two models could potentially give us the importance of this link. The link between obesity and insulin resistance is also laden with contradictory evidence but the mainstream thinking is that obesity increases insulin resistance. We run the model separately with no link and with to and fro links between the two nodes.

The apparent irreversibility of beta cell damage is debated. Although classically beta cells were believed not to regenerate once lost, experiments over the last two decades have shown that beta cells have good regeneration capacity in vitro and in vivo including de novo regeneration from ductal ascinar cells [21]. We operate the model independently assuming beta cell -1 state to be reversible as well as irreversible. We also encountered eleven other contradicting reports, where some studies had reported up-regulation while others observed down-regulation effect between the same node pair. We treated the contradictory links similar to the insulin resistance—hyperinsulinemia link i.e., the model was run separately assuming positive link or assuming negative link between the node pair.

## Results

### Point perturbations

For all point perturbations, after 20–25 cycles, the system invariably reached a stable state. Further, there were only two observed stable states that the system reached. Chaos or a homeostatic return to the starting state was never observed in the system. The two stable states did not drift further after any point perturbation and were thus true stable states by definition. If instead of zero, starting states of all nodes were randomly assigned, the same two stable system states were obtained. The bi-stability thus obtained is unlikely to be a statistical generality since a null model with the same number of nodes and links but with randomization of link placements rarely gave bi-stability. Out of 1000 null model simulations, 931 ended in a chaotic state. Stability was observed in 69 of them out of which, 12 showed a single stable state; 49 showed bi-stability, 4 showed tri-stability and the remaining 4 showed tetra-stability. The uncommon occurrence of bi-stability (p < 0.05) in the null model implies that the observed bi-stability in the network is unlikely to have arisen by chance alone.

In the two alternative stable system states, the states of all nodes including insulin action were stable, consistent and exactly opposite (in terms of +1 or up-regulated and -1 or down-regulated) to each other. Since insulin resistance is conventionally believed to be central to T2DM we called the two attractors as insulin sensitive and insulin resistant attractors. The former was characterized by low adiposity, cholesterol, glucose levels and inflammatory markers; and high adiponectin. The latter had a diametrically opposite picture (Table 1). The nodes which, when perturbed (up-regulated), led to the insulin sensitive attractor were collectively called the insulin sensitive basin of attraction and those which led to the insulin resistant attractor, when perturbed (up-regulated), were collectively called the insulin resistant basin of attraction.

**Table 1:** Attractors for the point perturbations.

Serial Number	Signals/ Nodes	Three Letter Code	State in the insulin resistant attractor	State in the insulin sensitive attractor
1.	Activin A	ata	1	-1
2.	Adiponectin	and	-1	1
3.	Adipose Tissue	adp	1	-1
4.	Aggression	agr	-1	1
5.	α-Melanocyte Stimulating Hormone (α-MSH)	msh	1	-1
6.	Angiogenesis	ang	-1	1
7.	Anti-oxidants	aox	-1	1
8.	Arginine Vasopressin	avp	0	0
9.	β-Adrenergic Receptors	bar	0	0
10.	β Cells	btc	-1	1
11.	Bone Strength/ Bone Mass	ost	-1	1
12.	Brain-Derived Neurotrophic Factor (BDNF)	bdn	-1	1
13.	Brain Glucose	bgl	-1	1
14.	Cholecystokinin	cck	-1	1
15.	Cholesterol	chl	1	-1
16.	Cocaine and Amphetamine Regulated Transcript (CART)	car	1	-1
17.	Cognitive Functions	cfn	1	-1
18.	Cortico-Releasing Hormone (CRH)	crh	-1	1
19.	Corticosteroids	cts	-1	1
20.	Cytokines	ctk	0	0
21.	Diplomat Behaviour	dip	1	-1
22.	Dopamine	dop	-1	1
23.	Endorphins	edp	-1	1
24.	Endothelin-1	et1	1	-1
25.	Epidermal Growth Factor (EGF)	egf	-1	1
26.	Erythropoeitin	epo	-1	1
27.	Exercise	exe	0	0
28.	Fertility	fty	-1	1
29.	Food Intake	fdi	1	-1
30.	Free Fatty Acids	ffa	1	-1
31.	γ-Aminobutyric acid (GABA) pancreas	gap	-1	1
32.	γ-Aminobutyric acid (GABA) brain	gab	0	0
33.	Gastrin	gst	1	-1
34.	Ghrelin	ghr	0	0
35.	Glucagon	glg	-1	1
36.	Glucagon-Like Peptide-1 (GLP-1)	glp	0	0
37.	Gluconeogenesis	gng	-1	1
38.	Glucose Transporter-1 (GLUT-1)	gt1	-1	1
39.	Gonadotropin-Releasing Hormone (GnRH)	grh	1	-1
40.	Growth Hormone	hgh	0	0
41.	Gut Motility	gmo	1	-1
42.	Histamine	hst	-1	1
43.	Inflammatory Response	inr	1	1
44.	Injury (Growth Factors)	inj	-1	1
45.	Insulin	ins	1	-1
46.	Insulin Action	ina	-1	1
47.	Insulin-like Growth Factor (IGF-1)	igf	-1	1
48.	Interleukin-6	il6	0	0
49.	Keto Acids	ktg	-1	1
50.	Klotho	klt	0	0
51.	Leptin	lep	1	-1
52.	Leptin Action	lpa	0	0
53.	Melatonin	mlt	0	0
54.	Muscle Strength/ Muscle Mass	msl	-1	1
55.	Myostatin	myo	1	-1
56.	Nerve Growth Factor (NGF)	ngf	-1	1
57.	Nitric Oxide	nox	1	-1
58.	Nociception	noc	1	-1
59.	Nor-epinephrine	nep	-1	1
60.	Oestrogen	otg	-1	1
61.	Osteocalcin	ocl	-1	1
62.	Oxytocin	oxy	-1	1
63.	Plasma Glucose	pgl	1	-1
64.	Secreted Frizzled-Related Protein 5 (SFRP-5)	sfr	1	-1
65.	Serotonin	ser	1	-1
66.	Sympathetic Stimulation	sys	0	0
67.	Testosterone	tet	-1	1
68.	Triglycerides	tri	1	-1
69.	Tumour necrosis factor-α (TNF-α)	tnf	1	-1
70.	Vasodilation	vdl	0	0
71.	Vitamin B12	v12	0	0
72.	Vitamin D3	vd3	0	0

The model used three different step lengths. For all the three step lengths, bi-stability was observed and the composition of the two attractors remained identical. There were subtle changes in the basins of attraction though. When the steps were fine grained, although the nodes attained transient fractional values in the initial cycles, they ultimately settled at +1 or -1 and the attractors remained identical. Between unit step and fine grained step the basins of attraction were over 90% similar. When direct leap was allowed bimodality and composition of attractors remained the same and the basins of attraction were similar to unit step model by over 80%. Since bi-stability and attractor composition were not sensitive to the step length, for further analysis we used the unit step model alone which was faster as well as accommodated changes in signals during transition.

### Sensitivity of the model to assumptions and contradictions

#### 1. Assumptions

To test the sensitivity of bi-stability to the underlying assumptions of the model, we relaxed the assumptions one by one and in combinations to see whether bi-stability was an artefact caused by some of them.

When we changed the mode of signal additions from simple arithmetic addition to qualitative addition, i.e. when a given node received both non-zero up-regulation and non-zero down-regulation links, the net signal strength was treated as zero. When a node received only positive signals, the node was up-regulated and when it received only negative signals, it was down-regulated. This invariably resulted in to chaos with every node and no long term tendency towards being up-regulated or down-regulated. A null model with qualitative additions invariably gave chaos. Therefore this result appears to be more of a statistical generality than any specific character of this network. The qualitative addition never allowed a sustained departure from the zero state. In the context of T2DM, this would mean that a stable insulin resistant or diabetic state may never be obtained. In reality, long term stability of insulin resistant or diabetic state is common and reversal is difficult. The qualitative addition mode did not appear to represent a realistic picture. Thus, relaxing some of the assumptions did not affect bi-stability and relaxing certain others gave rise to unrealistic chaotic results. None of the assumptions gave rise to good homeostatic control where the system returned to its ground state on its own. This demonstrated the robustness of bi-stability and the soundness of the set of assumptions used in the model.

#### 2. Contradictions

For all the contradictory interactions, simulations were run using positive or negative links. The interesting and surprising finding was that having or not having the compensatory hyperinsulinemia link did not affect the bi-stability of the network or the signatures of the two attractors. Since some researchers have argued for compensatory insulin resistance in response to primary hyperinsulinemia [19], we reversed the causal arrow between insulin resistance and insulin levels which again did not affect bi-stability. Similarly, reversing between the assumptions that obesity causes insulin resistance or insulin resistance causes obesity, or deleting the obesity-insulin resistance link altogether, did not affect bi-stability or the attractor signatures except for the state of obesity (i.e. the node ‘adipose tissue’) itself. When insulin and insulin action together down-regulated glucose, bi-stability was unaltered but when insulin alone down-regulated glucose independent of insulin action, the system oscillated with large periodicity (up to 32 cycles) and there were multiple resultant states. Therefore inclusion of the insulin sensitivity-resistance axis was one of the critical conditions for the bi-stability of the system.

For 10 out of the 11 up versus down-regulation contradictions examined, the system still retained bi-stability with the up-regulation or down-regulation arrows. Eight out of the 10 contradicting interactions that retained bi-stability showed no effects on the attractor signatures although the basins of attractions altered marginally (< 15%) in some of them. Two of the interactions brought about marginal changes in the attractor signatures. The only up versus down-regulation contradiction that affected bi-stability was when endothelial nitric oxide synthase (e-NOS) and neuronal nitric oxide synthase (n-NOS) action were considered a single node. Different studies have found either up-regulating [22,23] or down-regulating [24–27] action of NOS on aggression. Bi-stability was retained for the down-regulation link but not for the up-regulation link. After segregating the actions of e-NOS and n-NOS, bi-stability was retained. Since different studies report up or down regulating action of n-NOS on aggression, the model was run with either of the links at a time. With both types of links, bi-stability was maintained but the inclusion of n-NOS in the basin of attraction was affected.

Reactive oxygen species (ROS) is considered an important player in the pathophysiology of T2DM. During redundancy filtering, ROS was filtered out since it was tightly linked to inflammation and both shared identical incoming and outgoing links. But since ROS is believed to be an important player, we simulated keeping ROS as a separate node. This change again did not affect bi-stability and up-regulation of ROS led to insulin resistant state.

Glucagon has a direct up-regulation effect on insulin secretion [28], but through the agency of kisspeptin, it has a down-regulation effect [29], making the net effect zero. The signal between glucagon and insulin was therefore filtered out. However, since insulin and glucagon are believed to be central molecules to T2DM we operated the model with and without these links singly and in combination. The bi-stability remained robust to the inclusion or exclusion of these links. The effect of glucose on beta cell mass also has contradictory literature. Glucose is shown to stimulate proliferation of beta cells on the one hand [30] and on the other glucotoxicity is said to affect beta cell function [31]. Nevertheless the bi-stability of the model was not sensitive to either of the assumptions.

### Sustained perturbations

We perturbed each node singularly, in a sustained manner, and observed the downstream effects. Sustained perturbation of the nodes in the network did not affect bi-stability. A fraction of these perturbations led to stable short repetitive oscillations in the states of some nodes. Out of the 72 nodes 49 sustained perturbations gave identical results as respective point perturbations. Remaining 23 sustained perturbations showed some changes in the attractor signatures as compared to their respective point perturbations. Bi-stability was nevertheless maintained in all cases.

### Combining sustained and point perturbations

With each of the sustained perturbations in the background, every other node was point perturbed one at a time and simulations were run for a minimum of 300 cycles. Out of the 72 sustained perturbations, 60 led to bi-stability although the signatures of the attractors changed occasionally. Eleven sustained up-regulations gave rise to a single insulin sensitive attractor and these were aggression, adiponectin, dopamine, ghrelin, growth hormone, insulin action, melatonin, muscle strength, oestrogen, osteocalcin, and testosterone. And sustained up-regulation of serotonin invariably led to the insulin resistant attractor. Sustained up-regulation of the 11 nodes or down-regulation of serotonin never allowed the system to become insulin resistant. Not only that, but aggression, dopamine, ghrelin, insulin action, muscle strength, oestrogen and osteocalcin were able to completely reverse the states leading to the insulin sensitive attractor if the simulations began from the insulin resistant attracter as the starting conditions.

Although with combinations of perturbations the signatures of attractors could change, there were significant associations between the states of several nodes. We clustered the nodes based on the distance between pairs of nodes defined as the number of times the states of the two nodes did not match across all possible combinations of perturbations. The 3 different clusters obtained were (see S1 and S2 Figs for details of cluster analysis):

1. *and*, *agr*, *ang*, *aox*, *bdn*, *btc*, *cck*, *cts*, *crh*, *dop*, *egf*, *edp*, *epo*, *fty*, *gap*, *glg*, *gng*, *gt1*, *hst*, *igf*, *inj*, *ina*, *ktg*, *msl*, *ngf*, *nep*, *otg*, *ost*, *ocl*, *oxy*, *bgl*, *tet*
2. *ata*, *adp*, *msh*, *car*, *chl*, *cfn*, *dip*, *et1*, *ffa*, *fdi*, *gst*, *grh*, *inr*, *ins*, *lep*, *myo*, *nox*, *pgl*, *sfr*, *ser*, *tnf*, *tri*, *gmo*, *noc*
3. *avp*, *bar*, *ctk*, *gab*, *ghr*, *hgh*, *il6*, *klt*, *lpa*, *mlt*, *sys*, *vdl*, *vd3*, *exe*, *glp*, *v12*

### Validation of the network model

These clusters suggested a way of validating the model. We expected all the nodes in a cluster to be positively correlated to each other in real life data. Currently there are no studies that provide quantitative data on all the nodes together. However different studies have looked at different correlations. Of particular value are correlations between nodes that do not have a direct link between them but they lie in the same cluster in the above classification. Demonstrated correlations compatible with this expectations include myostatin to leptin [32], TNF-α to triglycerides, plasma glucose to cholesterol [33], vitamin D3 to vasodilation [34] and growth hormone to klotho [35]. We did not find any correlation in literature contrary to the model expectations.

### A comparison with the classical theory

**Fig 3:**
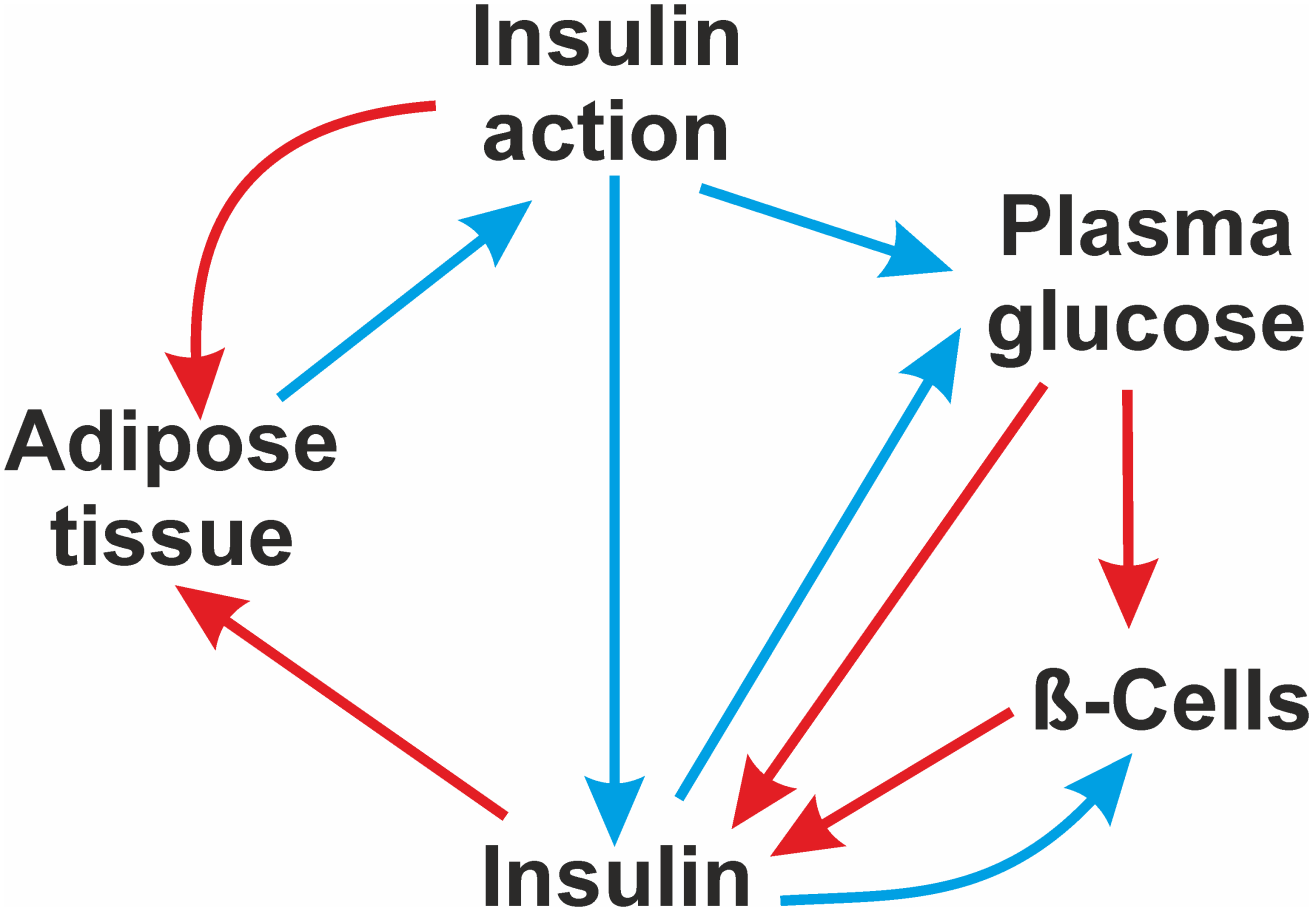
Classical model. Interactions among adipose tissue, insulin action, plasma glucose, plasma insulin and beta cell mass according to the classical theory are shown with red arrows indicating up-regulation links and cyan, down-regulation links.

The classical theory of insulin resistance states that obesity leads to insulin resistance, insulin resistance tends to increase plasma glucose which stimulates increased insulin secretion. This increased insulin secretion brings glucose back to normal leading to an insulin resistant-hyperinsulinemic-normoglycemic stable state. Failure of compensatory hyperinsulinemia owing to beta cell exhaustion or dysfunction results in to hyperglycemia. We included only adipose tissue, insulin, insulin action, beta cell mass and plasma glucose (Fig 3) as nodes in the model and included all known and classically believed links. In this classical model, we failed to see bi-stability under any condition. After any point perturbation in any of the five nodes, the system returned to the initial basal state in not more than 4–5 cycles or showed stable oscillations around the initial basal state. This is a typical behaviour of a homeostatic system. No point perturbation could change the basal state and lead to a stable insulin resistant state. Being a smaller and simpler system it is easier to visualize the reasons. For example, when we up-regulated adipose tissue mass, insulin resistance and subsequently plasma glucose increased. This increased insulin levels and subsequently glucose returned to normal. As glucose returned to normal, insulin could not remain elevated. Thus a normoglycemic-hyperinsulinemic state was not stable. Further in a state of high insulin resistance, the lipogenic action of insulin was suppressed and therefore adipose tissue was reduced. Reduction in adipose tissue normalized insulin resistance and thus the system was back to its starting state. Even if we assume that chronic overproduction of insulin affects beta cell function, beta cell mass remains in a homeostatic state since glucose is known to stimulate beta cell proliferation. Further, owing to the other homeostatic loops, both glucose and insulin return to normal thereby removing beta cell stress. Inclusion of glucotoxicity, that is, considering *pgl* to *btc* a negative regulator, did not drift the system away from homeostasis. Assuming beta cell loss as irreversible, that is, fixing *btc* state to -1 resulted into oscillation of insulin between zero and -1 states but glucose remained normal because of feedback loops operating through *adp* and *ina*. All the links in this small network made effective negative feedback loops and therefore the system failed to give a persistent insulin resistant state under any condition.

### Applications of the network model

#### 1. Is there any key node?

To check the sensitivity of the model to the nodes involved in the network and also to highlight the important nodes which when removed lead to the collapse of bi-stability, we deleted each node one at a time and observed the effect of perturbing every other node. A node under focus was frozen to the zero state all the time. This turned all the incoming as well as outgoing links from the node ineffective and thereby the node was cut-off from the rest of the network. This analysis also suggested whether tight homeostatic control over any node is sufficient for homeostasis of the entire system. We found that in 71 of the 72 deletions, there was no deviation from bi-stability. The system showed a deviation from bi-stability only when the node fertility (*fty*) was deleted. Deletion of *fty* led to multiple stable states; some being insulin sensitive and others being insulin resistant. Most of the correlates of insulin resistance remained similar except that high cholesterol was now associated with insulin sensitivity. To check whether any particular outgoing link of *fty* was responsible for this effect, we deleted each of them individually. None of the links made by *fty* when individually deleted affected the bi-stability. It seems to be a compound effect of the 3 links downstream to *fty* namely up-regulation of EGF, oestrogen and oxytocin. It is interesting to note that freezing glucose to the normal state did not ensure homeostasis of the entire network suggesting that glucose homeostasis is not central and critical to the behaviour of the network.

#### 2. Is there a key node combination?

In addition to single node deletion, we deleted combinations of nodes by randomly freezing to zero 10% of the nodes at a time. Out of 1000 such simulations, bi-stability was conserved 81% of the times. Among the remaining 19%, there was complete loss of stability 1.1% of the times. Among the deleted combinations that led to loss of stability the nodes aggression, dopamine and fertility were overrepresented. Among the other non-bi-stability outcomes 2.2% was contributed by uni-stability where the states of the nodes were at and around the basal zero state indicating that the network was in a robust homeostatic state. Among the combinations of deletions that gave robust homeostasis adiponectin, cholesterol, fertility, histamine, insulin action, leptin and oxytocin were overrepresented suggesting that these nodes in combination are critical for bi-stable behaviour of the system. It is interesting to note that glucose did not appear in this list indicating that ensuring glucose homeostasis along with a few other key nodes does not assure homeostasis of the entire system. See S3 Table for the list of combinations of deletions that led to homeostatic uni-stability and complete loss of stability. In the remaining 15.7% cases tri, tetra or penta-stability was obtained in which some states were insulin sensitive and others resistant.

#### 3. Is there a critical missing link?

We tested the robustness of the bi-stability of the model by random addition of a link between two randomly chosen nodes also. In 1,000 such random addition trials, bi-stability was not altered except for 8 specific link additions. In 6 out of the 8 there were 3 stable states instead of 2 and in only 2 cases there were multiple stable states. None of the additions resulted in chaos or homeostatic return to the starting state. This demonstrates further that the bi-stability is unlikely to be because of some critical missing link.

#### 4. What makes the bi-stability robust?

Since there were only two resultant attractors in the baseline model, the nodes could be classified as the ones whose up-regulation led to the insulin sensitive attractor and the other whose up-regulation led to insulin resistant attractor. Notably, point up-regulation of 40 of the 72 nodes, led to a stable state in which they remained up-regulated. This is a positive feedback effect. Sixteen of the nodes resumed the zero state although they drove the system to one of the two stable states. The remaining 16 showed an overcompensation-like response, i.e. point up-regulation of these 16 nodes led to a state in which they were down-regulated. Overall the network had a preponderance of positive feedback circuits which explains the robust bi-stable behaviour of the system.

**Fig 4:**
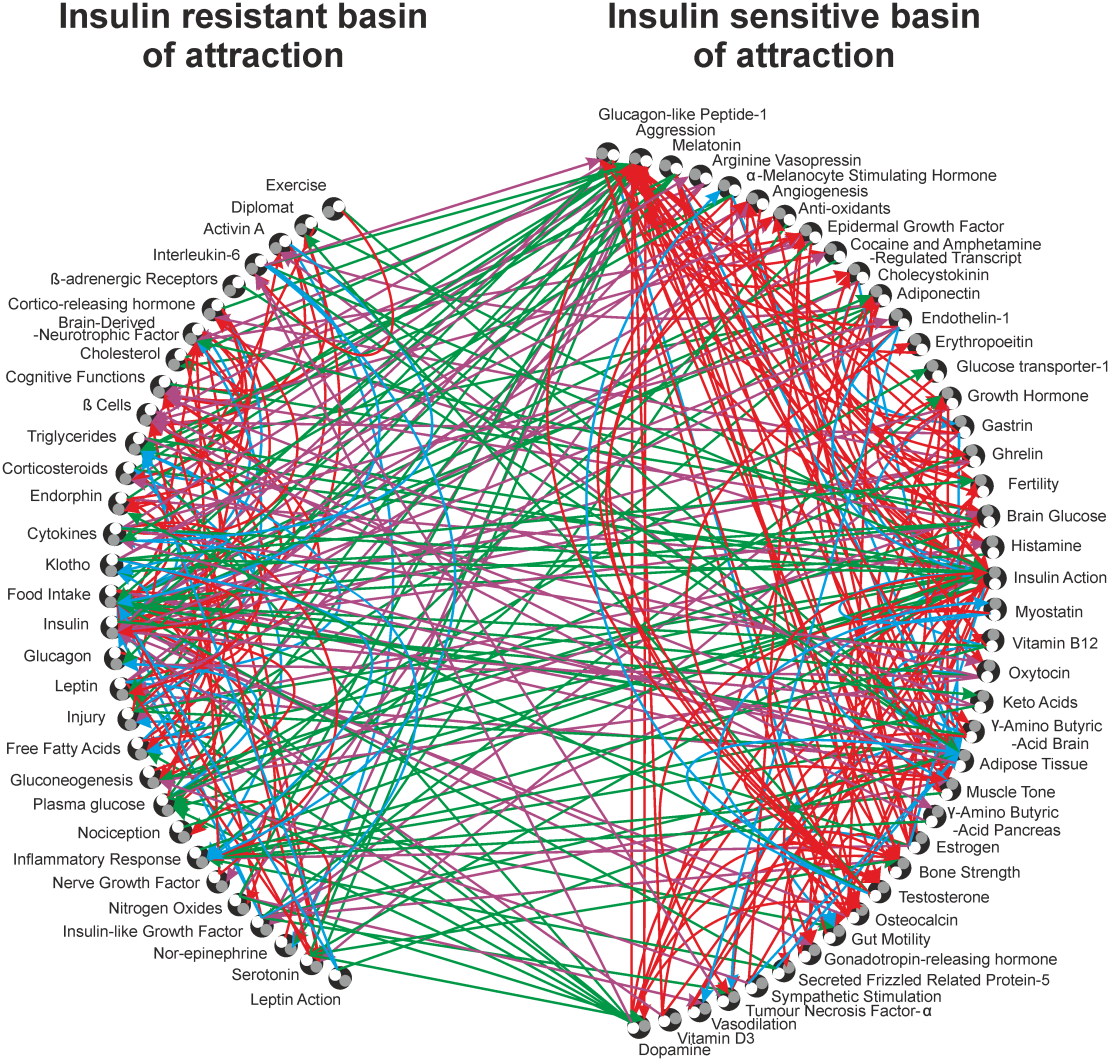
Basins of attraction. T2DM Signalling network segregated according to the point perturbations leading to the two attractors. The outbound (white circle) and inbound (grey circle) portals are shown for each node. Red arrows indicate intra-group up-regulation links; cyan, intra-group down-regulation links; purple, inter-groups up-regulation links; green, inter-groups down-regulation links.

**Fig 5:**
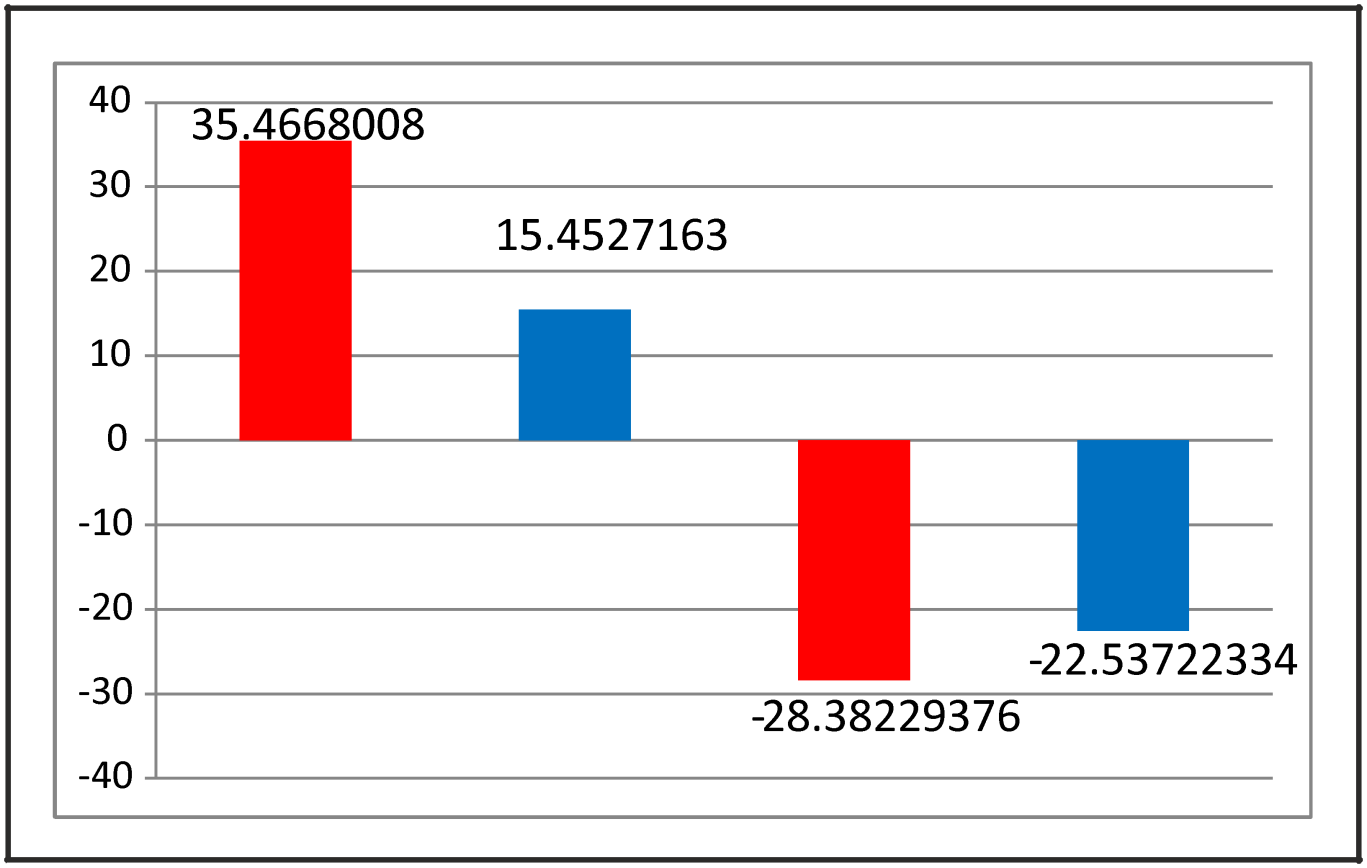
Link statistics. The bars represent the deviation from the expected number of links per cluster, the expected being calculated assuming independence. First two columns show the stabilizing links and the next two columns show the destabilizing links for the two clusters. The red and blue bars represent the insulin sensitive and the insulin resistant basins of attraction, respectively.

If the network is redrawn segregating the two groups of nodes (Fig 4), it can be appreciated that there are significantly more positive links within group as compared to between groups and there are significantly more negative links between groups as compared to within groups (chi square = 37.33619, df = 3, p< 0.0001). This makes the bi-stability and the dichotomous grouping of the nodes very robust. Within group positive and between groups negative links will stabilize and reinforce the attractors; whereas within group negative and between groups positive links will tend to destabilize the attractors. Since there were 216 stabilizing and 114 destabilizing links, there is no wonder that the two attractors were highly stable and not sensitive to changing a few nodes or links (Fig 5).

#### 5. Towards robust targets for treatment of T2DM

The combined perturbation simulation results give us possible new insights into long term effectiveness of a treatment. The critical question here is if a treatment target is sustainably locked into a desired state, how the network behaves in presence and absence of other perturbations. An ideal treatment target could be one which when locked should keep the system in an insulin sensitive state irrespective of any other perturbations. The different approaches currently targeted for treatment are suppression of liver gluconeogenesis, restoration of beta cell mass, incretin action, enhancement of insulin production, insulin supplementation, reduction in obesity, reduction in plasma free fatty acid levels, normalizing plasma glucose, reducing oxidative stress and exercise. None of these treatments was able to ensure an insulin sensitive state by sustained perturbation. The states were rather decided by the accompanying point perturbations. Thus none of these treatments were able to reverse the diabetic state in the long run although transient suppression of plasma glucose could be obtained with many of them. One major line of attempted treatment is to improve the beta cell function or introduce a new population of healthy beta cells. The critical underlying questions are whether beta cell regeneration in T2DM is reversible and whether improving beta cell function can reverse T2DM. When we operated the model assuming beta cell dysfunction to be reversible, in the insulin resistant attractor, the state -1 remained stable and up-regulating the state of beta cells, transiently (point perturbation) or sustainably, did not bring the system back to the insulin sensitive state. This suggests a possible solution to the beta cell paradox, that is, why beta cell dysfunction appears to be irreversible in T2DM when the cells have good regeneration capacity. In the model, other signals coming from the network kept beta cell function down-regulated. Alternatively, we assumed beta cell dysfunction to be irreversible, that is, when beta cells achieved a state of -1, it was retained -1 through all further cycles. Even under this assumption, bi-stability was attained and the composition of the attractors was substantially the same.

In contrast, there were 11 nodes namely aggression (*agr*), testosterone (*tet*), dopamine (*dop*), oestrogen (*otg*), osteocalcin (*ocl*), melatonin (*mlt*), ghrelin (*ghr*), muscle strength (*msl*), adiponectin (*and*), insulin action (*ina*) and growth hormone (*hgh*) which when sustainably up-regulated, ensured insulin sensitivity. All these nodes connect to insulin sensitivity by multiple pathways with positive regulator pathways far outnumbering negative regulatory pathways (Table 2). For example, aggression links directly and indirectly to the first tier players from Fig 1 through EGF [6,36,37], IGF-1 [38,39], dopamine [40,41], muscle mass [42], bone strength [43], adiponectin [44,45], testosterone [46,47] and other intermediates. Similar role is shown to be played by oestrogen in females [47,48]. Osteocalcin, a marker of bone formation [49], also increases insulin sensitivity in humans [50]. Melatonin is also known to enhance insulin sensitivity [51], and also aggression [52]. Thus most of the above mentioned nodes that could ensure insulin sensitive state were closely related to aggression and aggression may hold the key to an insulin sensitive state as suggested by Belsare et al.[53], Watve [2] and Watve and Yajnik [54].

**Table 2:** Number of pathways from the novel target to insulin action.

Novel Target	Total Pathways	Positive / Negative ratio
*and*	49	3.090909091
*agr*	140	1.955555556
*dop*	167	2.1
*ghr*	154	1.375
*hgh*	107	2.225806452
*ina*	49	1.705882353
*mlt*	138	1.976744186
*msl*	41	3
*otg*	110	2.678571429
*ocl*	68	1.56
*tet*	135	1.62
*ser*	99	0.446153846

All pathways that link the 12 promising nodes to insulin sensitivity were mapped and listed. The 11 nodes whose up-regulation increases insulin sensitivity, have a greater proportion of positive regulator pathways. Serotonin, whose down-regulation increases insulin sensitivity, had a greater proportion of negative regulator pathways. The 11 target to insulin action.l target to insulin action. The reference for each link and pathways far outnumbering negative r.

We further examined how much time did each of the potential candidate nodes took for a reversal from insulin resistant to sensitive state. In this race, oestrogen was the fastest actor which made the transition in 3 cycles followed by ghrelin (4), aggression (5), dopamine (7), muscle strength (24) and osteocalcin (59). If serotonin was down-regulated for at least 10 cycles, it also pushed the system from insulin resistant to insulin sensitive state. Applying a combination of interventions could reduce the number of cycles required for transition from insulin resistant to sensitive state. A minimum of 3 nodes were required to be simultaneously up-regulated for bringing up the transition in one or two cycles. Eleven three-membered combinations containing *agr* along with two other from *dop*, *tet*, *ghr*, *mlt*, *msl*, *otg*, and *hgh*; *dop* and *otg* with either *tet* or *hgh* could change the attractor from insulin resistant to the insulin sensitive state in a single cycle. Down-regulation of *ser* in combination with up-regulation of *agr* and either *dop*, *tet* or *ghr* could give the same effect. Once the system attained the insulin sensitive state by any of the above combinations of interventions, it could sustain itself against any point perturbations even when the interventions were withdrawn.

When these interventions were applied assuming beta cell degeneration to be irreversible, up-regulation of *agr*, *dop*, *otg*, *ocl* and *ina*; and down-regulation of ser could still lead to the insulin sensitive state. When these interventions were applied when both beta cell and insulin levels were kept fixed at -1, the results were identical. Thus the question whether beta cell degeneration is reversible or irreversible did not seem to be central to the reversal of an insulin resistant state to a sensitive one.

## Discussion

Despite the limitation of the model owing to its qualitative nature, the results are realistic in multiple ways. Running the model under different sets of assumptions, accommodating contradictory empirical results and the sensitivity analysis demonstrates that the model is robust and the results are not the artefactual outcome of any particular assumption. The model was able to predict the clinically observed correlates of insulin resistance accurately. It also made correct correlational predictions between pairs of variables that did not have a direct causal connection. The classically perceived treatments targeting liver glucose production, insulin sensitivity, insulin secretion including incretin action and beta cell function failed to bring about a transition in the steady state in the model although they could temporarily improve glucose control. This matches with the clinical observations that all these lines of treatments have largely failed to cure diabetes or even control hyperglycemia in the long run [55]. Many large scale clinical trials have revealed that normalizing blood glucose is not effective in avoiding diabetic complications [4]. This finding is compatible with the model. Further, the model demonstrates that it might be impossible, in principle, to prevent diabetic complications by a sole focus on normalizing glucose. The ineffectiveness of aggressive glucose normalization trials may not be because of failure to appropriately regulate glucose. Even if glucose is regulated without hypoglycemic and other undesirable events, the complications may not be arrested since normalization of glucose alone does not reverse the network state.

The model also accounts for foetal programming. If we consider the all zero baseline state of the system as a foetal condition, certain stimuli faced in embryonic or early life can drive the system to one of the two states which are difficult to reverse. This may account for developmental origins of adulthood disease (DOHAD) [56] or predictive adaptive response [57]. Since the model is based entirely on experimental data and it appropriately accounts for many realistic phenomena, the unexpected outcomes of the model need to be considered seriously as new possibilities. Empirical work in this direction is needed to test whether they work in reality.

Limitations of the model mainly come from 3 of its attributes that some of links might yet have to be discovered, the experiments from which data are taken are carried out on different model systems and that the model is discrete. Nevertheless, many predictions of the model matched with observed data suggesting thereby that the network model works reasonably well despite the limitations. This suggests that the novel and unexpected predictions of the model need to be tested empirically.

The model essentially demonstrates that the pathophysiology of type 2 diabetes is orders of magnitude more complex than the classical picture of insulin resistance and relative insulin deficiency causing hyperglycemia. Insulin and glucose have been the two molecules central to classical thinking but apart from the burden of history, there are no other grounds to treat insulin and glucose to be more important in T2DM than any other nodes of the network. The behaviour of the system is decided more by the network structure than by one or a few key molecules. In a network structure, it is possible to reach all nodes by starting from any random node. Therefore, although we started assembling the network from insulin and glucose, it does not mean the network is gluco-insulino-centric.

Because of the anastomoses of the network, the function lost by deleting a link can be compensated by alternative paths. Since the number of links stabilizing the attractors far outnumber the ones destabilizing it, a few missing links are unlikely to alter the behaviour of the network. This may explain why knockouts such as MIRKO, or insulin suppressing agents failed to increase fasting glucose in experiments [13,58]. It is possible in a network that one or a few nodes play a central role, but if this is true, it should have been detected by systematic deletion of nodes that we performed. The system was generally robust in this analysis and the only node whose deletion or freezing made any changes in the behaviour of the system was not related to energy homeostasis but to fertility and behaviour. This might be surprising for the classical theory of T2DM but is expected by some of the upcoming evolutionary hypotheses for the origin of T2DM [54,59]. Unless a single node or single link makes a critical difference, a disorder is unlikely to originate in a single gene defect. Therefore it is no wonder then that genome wide association studies are able to explain not more than 2% of obesity [60] and10% insulin resistance [61] at a population level.

The multiply reinforced alternative stable states suggest that there could have been strong selective forces to stabilize both the states under different contexts. Some of the evolutionary hypotheses argue that insulin resistance is not an inevitable result of obesity but is a contextually adaptive state selected to face certain environments or to support certain coping strategies [2,59]. Bi-stability indicates an adaptive and evolved insulin resistant state rather than a pathological deviation from a homeostatic system [62].

Clinically the first important realization of the study is that a large number of signals can potentially influence insulin sensitivity and the current emphasis on obesity alone is perhaps overplayed and unwarranted. The means of transiting from the insulin resistant attractor to the insulin sensitive one revealed by the model are substantially different from the traditional line of thinking in clinical practice or in drug discovery. The model shows that none of the current lines of treatment are able to make this transit. Instead the model suggests some non-conventional lines of treatment. Of particular interest is the role of exercise. Sustained physical activity alone did not have effects comparable to aggression in the model. Physical activity has been classically considered to affect energy balance and reduce adiposity. Physical aggression on the other hand has many other direct endocrine effects [53] and this effectively assured insulin sensitivity in the model. This raises the possibility that exercises work more effectively through the behavioural neuro-endocrine pathways rather than through calorie consumption. In reality, many types of exercises have some or the other behavioural components and thereby stimulate the neuro-endocrine pathways [63–67] in addition to burning calories. A testable prediction of the model is that different exercises can be expected to have different endobolic effects even if the caloric requirement is matched [53,68].

We can no more view complex disorders by piecemeal and expect to treat the disorder effectively. The behaviour of a network can be substantially different from the behaviour of smaller pieces of the network. The model suggests molecular targets such as adiponectin, growth hormone, melatonin and testosterone for prevention of T2DM; and dopamine, ghrelin, oestrogen and osteocalcin for prevention as well as treatment of T2DM. But since all these molecules are behaviourally regulated, it is likely that behavioural intervention may have a better promise. It is quite likely that a paradigm shift is awaiting round the corner in the field and we need to be open to this possibility.

## Supporting information

S1 Fig Frequency distribution of distances of pairs of nodes. We clustered the nodes based on Simple Matching Coefficient (SMC) between pairs of nodes defined as the number of times the states of the two nodes matched across all possible combinations of perturbations. This led to a SMC matrix of 71 X 70 nodes to which the basic set of 71 point perturbations and 71 singular sustained perturbations were added to make the total 5112. All the scores were normalized by this total number 5112. Hence, every possible pair of nodes had a score from zero to one. To view this scoring as a distance between the two nodes under consideration, we subtracted that number from one. Hence, the pairs of nodes having a score nearer to zero mean that the nodes in the pair are strongly correlated and hence closer to each other and the pairs having a score of one denotes the longest possible distance and thereby no correlation between the nodes in that pair. These scores were used to construct a frequency distribution. Since the histogram shows two distinct peaks, it indicates clear clustering. The two peaks in the frequency distribution of pair-wise distances correspond to the intra-group distance and the inter-group distance respectively. We considered the first dip, i.e. 0.4 in the histogram as a threshold and listed all the pairs which had a distance less than that threshold. Clustering was made by associations starting with the first pair till the list was exhausted. In this way, 3 different clusters were obtained.
(TIF)

S2 Fig Dendrogram generated by DendroUPGMA. To compare the method of clustering with a known method of clustering, we used DendroUPGMA (http://genomes.urv.cat/UPGMA/), open source online software to cluster the nodes in our network and plot a dendrogram. The software uses UPGMA (Unweighted Pair Group Method with Arithmetic mean) for clustering. We used the input data type as similarity matrix and fed in the 71 X 71 matrix with the original scores out of 5112 for each pair of nodes. Clusters identified by both the clustering protocols were identical.
(TIF)

S1 Table Model organism used in each reference. (DOCX)

S2 Table Nodes and links with references. (DOCX)

S3 Table List of deletions of the 10% of the nodes that led to uni-stability and complete loss of stability. (DOCX)

S1 Text Merger and exclusion of links according to criteria defined in the text. (DOCX)
