# Supplementary figures and images for: Bi-stability in type 2 diabetes mellitus multi-organ signalling network
Source: PLoS One. 2017 Aug 2;12(8):e0181536. doi: 10.1371/journal.pone.0181536 (PMC5540287; doi:10.1371/journal.pone.0181536)

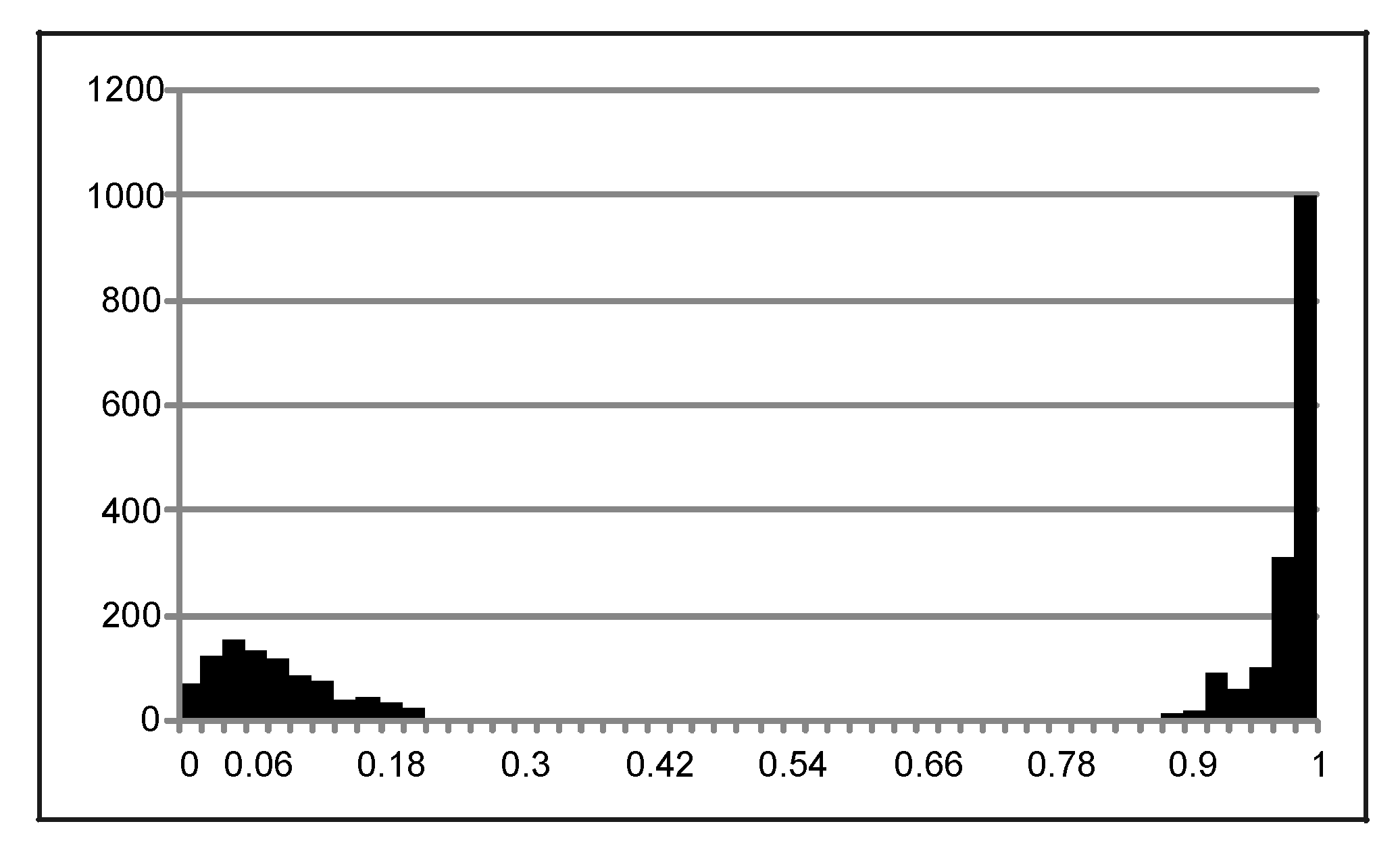

Supplement: S1 Fig — We clustered the nodes based on Simple Matching Coefficient (SMC) between pairs of nodes defined as the number of times the states of the two nodes matched across all possible combinations of perturbations. This led to a SMC matrix of 71 X 70 nodes to which the basic set of 71 point perturbations and 71 singular sustained perturbations were added to make the total 5112. All the scores were normalized by this total number 5112. Hence, every possible pair of nodes had a score from zero to one. To view this scoring as a distance between the two nodes under consideration, we subtracted that number from one. Hence, the pairs of nodes having a score nearer to zero mean that the nodes in the pair are strongly correlated and hence closer to each other and the pairs having a score of one denotes the longest possible distance and thereby no correlation between the nodes in that pair. These scores were used to construct a frequency distribution. Since the histogram shows two distinct peaks, it indicates clear clustering. The two peaks in the frequency distribution of pair-wise distances correspond to the intra-group distance and the inter-group distance respectively. We considered the first dip, i.e. 0.4 in the histogram as a threshold and listed all the pairs which had a distance less than that threshold. Clustering was made by associations starting with the first pair till the list was exhausted. In this way, 3 different clusters were obtained. (TIF) [file pone.0181536.s001.tif]

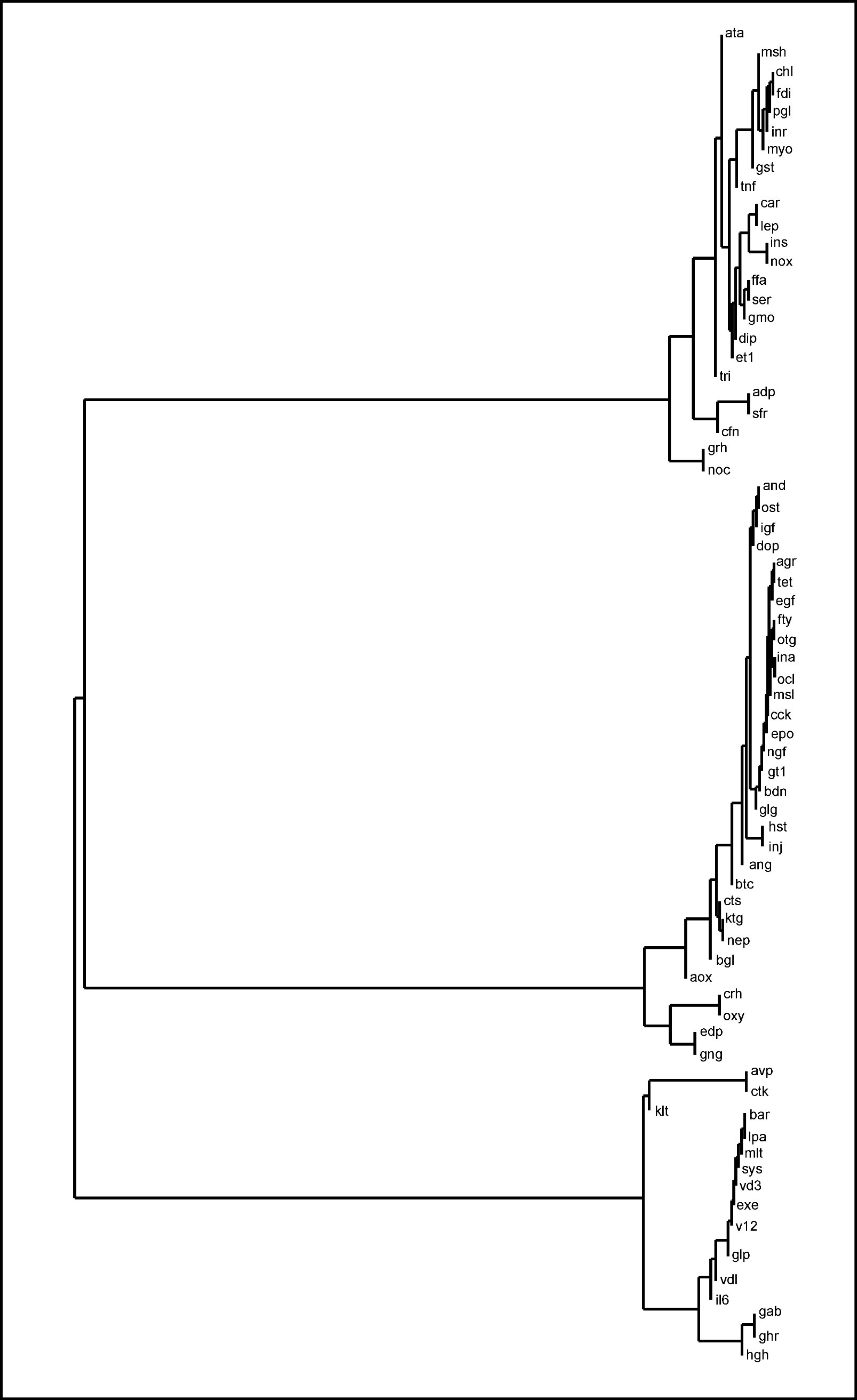

Supplement: S2 Fig — To compare the method of clustering with a known method of clustering, we used DendroUPGMA (http://genomes.urv.cat/UPGMA/), open source online software to cluster the nodes in our network and plot a dendrogram. The software uses UPGMA (Unweighted Pair Group Method with Arithmetic mean) for clustering. We used the input data type as similarity matrix and fed in the 71 X 71 matrix with the original scores out of 5112 for each pair of nodes. Clusters identified by both the clustering protocols were identical. (TIF) [file pone.0181536.s002.TIF]
