# Supplementary material for: Bi-stability in type 2 diabetes mellitus multi-organ signalling network
Source: PLoS One. 2017 Aug 2;12(8):e0181536. doi: 10.1371/journal.pone.0181536 (PMC5540287; doi:10.1371/journal.pone.0181536)
Supplement: S1 Table — (DOCX) [file pone.0181536.s003.docx]

**Supporting information**

**Supporting table**

**S1 Table. Model organism used in each reference.**

| **Factor** | **Abbreviation** | **Effect** |  | **Model organism** |
| --- | --- | --- | --- | --- |
| Activin A | ata | btc1 |  | Sprague-Dawley rat (1) |
| Activin A | ata | ctk0 |  | HUVECs (2) |
| Activin A | ata | ins1 |  | Cultured human pancreatic islets (3) |
| Activin A | ata | adp0 |  | Human adipose progenitors (4) |
| adipose tissue | adp | ata1 |  | Human adipose progenitors (4) |
| adipose tissue | adp | tnf1 |  | Human adipose tissue (5) |
| adipose tissue | adp | ctk1 |  | Human adipose tissue (6) |
| adipose tissue | adp | and1 |  | Human adipose tissue (7) |
| adipose tissue | adp | lep1 |  | Human adipose tissue (8); Rat epididymal fat pad (9); Human adipose tissue (10); C57BL/6J mice (11) |
| adipose tissue | adp | sfr1 |  | C57BL/6 mice (12); Mice (13) |
| adiponectin | and | ina1 |  | Mice (14); Mice (15) |
| adiponectin | and | ata1 |  | Primary human monocytes (16) |
| adiponectin | and | ffa0 |  | C57BL/6J mice (17) |
| adiponectin | and | fdi0 |  | Wistar rats (18) |
| adiponectin | and | inr0 |  | Human aortic endothelial cells (19) |
| adiponectin | and | fty1 |  | Bovine ovarian cells and embryo (20); Mice embryos (21); Human and C57BL/6J mice granulosa and cumulus cells (22) |
| adiponectin | and | tri0 |  | C57BL/6J mice (17) |
| aggression | agr | chl0 |  | Human subjects (23) |
| aggression | agr | egf1 |  | Mice (24); Osteoblast-like cell line (25); Mice (26) |
| aggression | agr | ngf1 |  | Mice (27); Mice (28); Mice (29); Mice (30); Mice (31) |
| aggression | agr | bdn1 |  | Hamsters (32) |
| aggression | agr | dop1 |  | Long-Evans rats (33); Long-Evans rats (34) |
| aggression | agr | ser0 |  | Long-Evans rats (33); Long-Evans rats (34) |
| aggression | agr | edp1 |  | Human subjects (35); Human subjects (36) |
| aggression | agr | tet1 |  | Human subjects (37) |
| aggression | agr | cts1 |  | Osteoblast-like cell line (25); Human subjects (37) |
| aggression | agr | cck1 |  | Long-Evans rats (38) |
| aggression | agr | et11 |  | Mice (39) |
| aggression | agr | igf1 |  | Pudu deer (40); Baboons (41) |
| alpha MSH | msh | tri0 |  | OLETF rats (42) |
| alpha MSH | msh | ina1 |  | OLETF rats (42); Sprague-Dawley rats (43) |
| alpha MSH | msh | agr1 |  | Mice (44); Wistar rats (45); Mice (46) |
| alpha MSH | msh | adp0 |  | Sprague-Dawley rats (43) |
| alpha MSH | msh | msl1 |  | Wistar rats (47) |
| alpha MSH | msh | ctk0 |  | Blood samples (48) |
| alpha MSH | msh | fdi0 |  | Long-Evans rats (49); OLETF rats (42); Sprague-Dawley rats (50) |
| angiogenesis | ang | bgl1 |  | Logic |
| anti-oxidant | aox | inr0 |  | Mice (51) |
| arginine vassopressin | avp | agr1 |  | Hamsters (52) |
| arginine vassopressin | avp | cfn1 |  | Human subject (53); Human subjects (54); Human subjects (55) |
| arginine vasopressin | avp | ins1 |  | Human subjects (56) |
| arginine vassopressin | avp | gng1 |  | Porton-Wistar rats (57) |
| BDNF | bdn | btc1 |  | C57BL/KsJ-db/db mice (58) |
| BDNF | bdn | ina1 |  | Mice (59); Zucker fatty rats (60); C57BL/KsJ-db/db mice (61) |
| BDNF | bdn | cfn1 |  | Human subject (62) |
| BDNF | bdn | ser1 |  | C57BL/6 mice (63) |
| BDNF | bdn | fdi0 |  | Human subject (62); C57BL/6 mice (64); Mice (59) |
| beta adrenergic receptors | bar | adp0 |  | Human adipocytes (65); Beagle dogs (66) |
| beta cells | btc | ins1 |  | Human and rat pancreatic islets (67) |
| beta cells | btc | gap1 |  | Rats and Human insulinoma(68) |
| CART | car | fdi0 |  | Mice (69); Wistar rats (70) |
| cholecystokinin | cck | fdi0 |  | 129/SvEv mice (71); Human subjects (72); OLETF rats (73); Sprague-Dawley rats (74); Rhesus monkey (75); Rats (76); Mice (77) |
| cholecystokinin | cck | ina1 |  | Mice (78) |
| cholecystokinin | cck | gst0 |  | Pigs (79) |
| cholecystokinin | cck | ins1 |  | Human subjects (80) |
| cholesterol | chl | ser1 |  | Macaques (81) |
| cholesterol | chl | inr1 |  | Mice (82) |
| cholesterol | chl | agr0 |  | Monkeys (83); Macaques (81) |
| cholesterol | chl | cfn1 |  | Human subjects (84) |
| cognitive function | cfn | bgl0 |  | Logic |
| cognitive function | cfn | dip1 |  | Logic |
| corticosteroids | cts | tri0 |  | Sprague-Dawleys rats (85); Human subjects (86) |
| corticosteroids | cts | ffa1 |  | Sprague-Dawleys rats (85); Human subjects (86) |
| corticosteroids | cts | gng1 |  | Hepatoma cells (87); H4IIE rat hepatoma cells (88); H4IIE rat hepatoma cells (89) |
| corticosteroids | cts | ina0 |  | Rats (90); Zucker rat (91); Rat muscle tissue (92); Mice (93); Human subjects (94); Human subject (95); Human subjects (96); Human subjects (97) |
| corticosteroids | cts | agr0 |  | Mice (98) |
| corticosteroids | cts | inr0 |  | Human subjects (99); Normal human epithelial cells (100); Human subjects (101); Human subjects (102) |
| corticosteroids | cts | ins0 |  | Hamster HIT-15 Beta cells (103); Mice pancreas (104) |
| cortico releasing hormone | crh | fdi0 |  | Mice (105) |
| cortico releasing hormone | crh | cts1 |  | C57BL/6J mice (106); Rats (107); Human subjects (108); normal human epidermal melanocytes (109); Human fetal adrenal cells (110); Mice (111) |
| cortico releasing hormone | crh | agr0 |  | Harlan-Sprague-Dawley mice (112); Rats (113) |
| cortico releasing hormone | crh | ins1 |  | Mice pancreatic islets (56) |
| cortico releasing hormone | crh | edp1 |  | Pituitary (114); Mouse pituitary cell line (ATt-20) (115) |
| cytokines | ctk | edp1 |  | Mouse pituitary cell line (ATt-20) (115); Mouse pituitary cell line (116) |
| cytokines | ctk | crh1 |  | Baboons (117); Rats (118); Rats (119) |
| cytokines | ctk | lep1 |  | Hamsters (120); C57BL/6 mice (121) |
| cytokines | ctk | inr1 |  | Logic |
| cytokines | ctk | oxy1 |  | Rats (122) |
| cytokines | ctk | avp1 |  | Rats (122) |
| cytokines | ctk | ina0 |  | Human subject (123); Human megakaryotic cell line CHRF-288-11 (124) |
| cytokines | ctk | klt0 |  | C57/BL6 rats (125) |
| cytokines | ctk | et11 |  | Porcine endothelial cells (126) |
| diplomat | dip | ins1 |  | Chimpanzees (127) |
| dopamine | dop | nep0 |  | Sprague-Dawley rats (128) |
| dopamine | dop | agr1 |  | Rats (129); Sprague-Dawley rats (128) |
| dopamine | dop | ost1 |  | Mice (130) |
| dopamine | dop | ocl1 |  | Human subjects (131) |
| dopamine | dop | ina1 |  | C57BL6 mice (132) |
| dopamine | dop | msl1 |  | Sprague-Dawley rats (133) |
| dopamine | dop | ins0 |  | INS-1E cells and pancreatic islets (134); C57BL/5J mice (135) |
| dopamine | dop | lep0 |  | Human adipocytes (136) |
| dopamine | dop | il61 |  | Human adipocytes (136) |
| dopamine | dop | and1 |  | Human adipocytes (136) |
| dopamine | dop | ffa0 |  | C57BL/5J mice (135) |
| dopamine | dop | tri0 |  | C57BL/5J mice (135) |
| dopamine | dop | pgl0 |  | C57BL/5J mice (135) |
| dopamine | dop | fdi0 |  | Zucker rats (137); C57BL/5J mice (135) |
| egf | egf | noc0 |  | Dorsal root ganglia neurons (138) |
| egf | egf | btc1 |  | Canine islets and murine bet cells (139); FVB E1-DN mice (140) |
| egf | egf | bdn1 |  | CD-1 mice (141) |
| egf | egf | ina1 |  | Human adipose tissue (142) |
| egf | egf | fty1 |  | Mice (143); Mice (144); Mice (145); C3H/HeN mice (146) |
| endorphin | edp | bdn1 |  | Sprague-Dawley rats (147) |
| endorphin | edp | ina1 |  | Rats (148) |
| endorphin | edp | agr0 |  | Mice (149) |
| endorphin | edp | fdi1 | ? | Rats (50); Rats (150) |
| endorphin | edp | fdi0 | ? | Zucker rats (151) |
| endothelin | et1 | ghr1 |  | Holstein Steers (152); Holstein Steers (153) |
| endothelin | et1 | ina0 |  | Rat muscles (154); Sprague-Dawley rats (155); Human subjects (156) |
| endothelin | et1 | agr1 |  | Mice (39) |
| endothelin | et1 | ins1 |  | Mice islets of Langerhans (157) |
| endothelin | et1 | lep1 |  | Adipocyte cell lines (158) |
| endothelin | et1 | vdl0 |  | Pigs, Humans and Rats (159); Human epicardial coronary arteries (160); Porcine endothelial cells (161) |
| EPO | epo | ang1 |  | Human glioma (162);Human mesenchymal stem cells (163) |
| fertility | fty | egf1 |  | Mice (164) |
| fertility | fty | oxy1 |  | Rats (165) |
| fertility | fty | otg1 |  | Mice (166) |
| FFA | ffa | bdn0 |  | Human subjects (167) |
| FFA | ffa | gng1 |  | Human subjects (168) |
| FFA | ffa | inj0 |  | Human subjects (169) |
| FFA | ffa | ina0 |  | Human subjects (167); Human subjects (170); Human subjects (171); Human subjects (169) |
| food intake | fdi | adp1 |  | Sprague-Dawley rats (172) |
| food intake | fdi | agr0 |  | Meerkats (173) |
| food intake | fdi | msl1 |  | Logic |
| food intake | fdi | ffa1 |  | Logic |
| food intake | fdi | ins1 |  | Rats (174) |
| food intake | fdi | pgl1 |  | Logic |
| GABA Brain | gab | agr1 | ? | Mice (175) |
| GABA Brain | gab | agr0 | ? | Human subjects (176); Mice (177) |
| GABA Brain | gab | grh1 |  | Mice (178) |
| GABA Brain | gab | fdi0 | ? | Rats (179); Rats (180) |
| GABA Brain | gab | fdi1 | ? | Sprague-Dawley rats (181); Rats (182) |
| GABA pancreas | gap | inr0 |  | Mice(183) |
| GABA pancreas | gap | btc1 |  | Human islets and Rats(184) |
| gastrin | gst | btc1 |  | Rat pancreas (185) |
| gastrin | gst | agr1 |  | Mice (186) |
| gastrin | gst | ina1 |  | Mice (187) |
| gastrin | gst | adp0 |  | Mice (187) |
| ghrelin | ghr | agr1 |  | BALB/c mice (188) |
| ghrelin | ghr | fdi1 |  | Mice (189); Rats (190); Human subjects (191); Wistar rats (192); Mice and Rats (193); Sprague-Dawley rats (194); Sprague-Dawly rats (195) |
| ghrelin | ghr | hgh1 |  | Holstein Steers (153); Rats (196); Rats (197); Wistar rats (192); Human subjects (198); Mouse hypothalamus (199); Human subjects (200); Human subjects (191) |
| ghrelin | ghr | ins1 |  | Sprague-Dawley rats (201) |
| ghrelin | ghr | gab1 |  | Sprague-Dawley rats (202); Rats and mice (203) |
| ghrelin | ghr | et10 |  | Sprague-Dawley rats (204) |
| glucagon | glg | gng1 |  | Rats (205); Rats (206); Bovine (207) |
| gluconeogenesis | gng | pgl1 |  | Rats (205); Rats (206) |
| glut1 | gt1 | bgl1 |  | Rats (208) |
| GnRH | grh | tet1 |  | European ground squirrels (209); Sprague-Dawley rats (210) |
| growth hormone | hgh | msl1 |  | Human subjects (211); Human subjects (212); Human subjects (213) |
| growth hormone | hgh | agr1 |  | Mice (214) |
| growth hormone | hgh | ins1 |  | Human subjects (215); Human subjects (216) |
| growth hormone | hgh | ina0 |  | Human subjects (215); Human subjects (216); 3T3-L1 adipocytes (217); Human subjects (218); Human subjects (219); balb/c mice (220) |
| growth hormone | hgh | igf1 |  | Rats (221); Human subjects (219); Human subjects (215); Human subjects (222); Mice (223) |
| growth hormone | hgh | fty1 |  | Rats (221) |
| histamine | hst | fdi0 |  | H1KO mice (224); Sprague-Dawley rats (225); Wistar King A rats (226); Mice (227) |
| histamine | hst | agr1 |  | Mice (228) |
| IGF 1 | igf | ina1 |  | Human subjects (229); Human subjects (230); Human subjects (231); Sprague-Dawley rats (232); Human subjects (222); Human subjects (233); Mice (234); Mice (235); Wistar rats (236) |
| IGF 1 | igf | btc1 |  | Mice (237) |
| IGF 1 | igf | fdi0 |  | Wistar rats (238); Wistar rats (236) |
| IGF 1 | igf | adp0 |  | Sprague-Dawley rats (232); Wistar rats (236) |
| IGF1 | igf | msl1 |  | C57BL/6 mice (239); Mice (240); Mice (241); Rabbits (242) |
| IGF1 | igf | ins0 |  | Rat pancreatic beta cells (243); Mice (235) |
| IGF1 | igf | hgh0 |  | Primary rat pituitary cells (244); Sheep (245); Mice (234) |
| IGF1 | igf | ost1 |  | Mice (246) |
| IGF1 | igf | ctk0 |  | Sprague-Dawley rats (247) |
| IGF1 | igf | tnf0 |  | Sprague-Dawley rats (247) |
| Il-6 | il6 | agr0 |  | Mice (248) |
| Il-6 | il6 | ina0 |  | Human adipose tissue (249); Mouse hepatocytes (250) |
| Il-6 | il6 | adp0 |  | Mice (251) |
| Il-6 | il6 | inr0 |  | Human subjects(252) |
| Il-6 | il6 | glp1 |  | Mice(253) |
| Il-6 | il6 | fdi0 |  | Mice(254) |
| inflammatory response | inr | ina0 |  | Mice (255) |
| injury (growth factors) | inj | btc1 |  | Human pancreatic islets (256); Mice (257) |
| injury (growth factors) | inj | hst1 |  | Dogs (258) |
| injury (growth factors) | inj | adp0 |  | Mice (259) |
| injury (growth factors) | inj | ins0 |  | Mice (259) |
| injury (growth factors) | inj | ina1 |  | Mice (259) |
| injury (growth factors) | inj | agr0 |  | Mice (260) |
| injury (growth factors) | inj | inr0 |  | Human fibroblast cells (261) |
| injury (growth factors) | inj | ang1 |  | Mice (262) |
| insulin | ins | cfn1 |  | Human subjects (263); Human subjects (264); Human subjects (265); Human subjects (266); Mice (267) |
| insulin | ins | ktg0 |  | Rat adipose tissue (268) |
| insulin | ins | btc1 |  | Canine islets and murine beta cells (139) |
| insulin | ins | lep1 |  | Rat white adipose tissue (9); Human subjects (269) |
| insulin | ins | klt1 |  | COS-7 cells (270) |
| insulin | ins | egf1 |  | C57BL/KsJ mice (271); Mice (145) |
| insulin | ins | et11 |  | Human subjects (272) |
| insulin | ins | grh1 |  | GnRH expressing cell line (273) |
| insulin | ins | and0 |  | Bovine adipocytes (274); Human subjects (275) |
| insulin | ins | nox1 |  | Bovine endothelial cells (276); Human subjects (277) |
| insulin | ins | gmo1 |  | Rats (278) |
| insulin action | ina | pgl0 |  | Rats (279); Rats (238) |
| insulin action | ina | gng0 |  | Bovine (207); Mice (280); Mice (281) |
| insulin action | ina | msl1 |  | Human muscle tissue(282) |
| insulin action | ina | ost1 |  | Mice (283); Mice and cell lines (284) |
| insulin action | ina | adp1 |  | Mouse embryonic fibroblasts (285); Mice (286) |
| insulin action | ina | tri1 |  | Rats (287); Mice (288) |
| keto acids | ktg | cfn1 |  | Rats (289) |
| keto acids | ktg | ins1 |  | Pancreatic beta cells(290); Rat pancreatic islets (291) |
| Keto acids | ktg | inr0 |  | Mammalian cell culture (292) |
| klotho gene | klt | fty0 |  | Mice (293); Mice (294) |
| klotho gene | klt | ina0 |  | Mice (295); Mice (296) |
| klotho gene | klt | aox1 |  | Mice (297) |
| klotho gene | klt | ang1 |  | Mice (298) |
| klotho gene | klt | inr0 |  | KM mice (299); Mice (300) |
| klotho gene | klt | adp1 |  | Mouse 3T3-L1 cells (301); Mice (295) |
| klotho gene | klt | ins1 |  | MIN6 beta cells (302) |
| leptin | lep | inr1 |  | Human adipocytes (10) |
| leptin | lep | car1 |  | Wistar rats (70); Sprague-Dawley rats (303); Rats (304) |
| leptin | lep | ffa0 |  | Rats (305) |
| leptin | lep | ang1 |  | HUVECs and PAECs (306); Normal HUVECs and HCASMCs (307); Rats and human endothelial cells (308); Wistar rats (309) |
| leptin | lep | cfn1 |  | Mice (310) |
| leptin | lep | ser1 | ? | Black Swiss mice (311) |
| leptin | lep | ser0 | ? | Mice (312) |
| leptin | lep | et11 |  | Rat portal vein (313);HUVECs (314) |
| leptin action | lpa | fdi0 |  | Rats (304); Mice (315); Rat pancreatic islets (316); Wistar rats (317); Rats (318) |
| leptin action | lpa | adp0 |  | Mice (315); Mice (319); Wistar rats (317) |
| leptin action | lpa | tri0 |  | Mice (315); Rat pancreatic islets (316) |
| melatonin | mlt | agr1 |  | Syrian hamsters (320) |
| melatonin | mlt | ost1 |  | MC3T3 cells (321); Osteoblast-like cell line (322) |
| melatonin | mlt | ocl1 |  | MC3T3 cells (321) |
| melatonin | mlt | adp0 |  | Rats (323); Sprague-Dawley rats (324); Sprague-Dawley rats (325); osteoblast-like cell line(322) |
| melatonin | mlt | lep0 |  | Rats (326); Rats (327) |
| melatonin | mlt | ins0 |  | Rats (323); Rats (326); Sprague-Dawley rats (324); Rats (327) |
| melatonin | mlt | pgl0 |  | Sprague-Dawley rats (325) |
| melatonin | mlt | tri0 |  | Sprague-Dawley rats (325); Rats (327) |
| melatonin | mlt | ina1 |  | Rats (328); SAMP8/SAMR1 mice (329); Mice (330); Mice (331); Rats (332) |
| melatonin | mlt | msl1 |  | Rats (333); Rats (334) |
| muscle strength | msl | agr1 |  | Human subjects (335) |
| muscle strength | msl | ina1 |  | Human subjects (335) |
| muscle strength | msl | inr0 |  | Human subjects (252) |
| myostatin | myo | msl0 |  | Mice (336); Mice (337); Mice (338); Mice (339); Sprague-Dawley rats (340); Mice (341) |
| myostatin | myo | ina0 |  | C57BL/6 mice (342); Human subjects (343); Mice (339); C57BL/6 (B6) mice (344) |
| myostatin | myo | tnf1 |  | C57BL/6 (B6) mice (344) |
| myostatin | myo | adp1 |  | Mice(339); Mice (341); C57BL/6 (B6) mice (344); C57BL/6 mice (342) |
| NGF | ngf | bdn1 |  | Human and rat pancreatic islets (345); CD-1 mice (141) |
| NGF | ngf | noc1 |  | Dogs (346); Lewis rats (347); Human subjects (348) |
| NO | nox | vdl1 |  | Human subjects (349); Human subjects (277) |
| NO | nox | ang1 |  | Mice (350); Mice (262) |
| NO | nox | nep0 |  | Wistar rats (351) |
| NO | nox | dop0 |  | Wistar rats (351) |
| NO | nox | agr1 | ? | Mice (352); Mice (353) |
| NO | nox | agr0 | ? | Mice (354); Mice (355); Mice (356); Mice (357) |
| norepinephrine | nep | agr0 |  | Sprague-Dawley rats (128) |
| norepinephrine | nep | ina0 |  | Hamsters (358) |
| norepinephrine | nep | crh1 |  | Rats (359) |
| oestrogen | otg | hgh1 |  | Human subjects (360); Rat osteosarcoma cells (UMR 106.01) (361); Human subjects (362) |
| oestrogen | otg | ina1 | ? | Human subjects (363); Rats (364) |
| oestrogen | otg | ina0 | ? | Human subjects (365) |
| oestrogen | otg | fdi0 |  | C57BL/6J and Swiss Webster mice (366) |
| oestrogen | otg | agr1 |  | Rats (367); Rats (368); California mice (369); CD-1 mice (370); C57BL/6J mice (371); C57BL/6J mice (372); C57BL/6J mice (373) |
| oestrogen | otg | ang1 |  | Human endometrial cells and HMMECs (374); BALB/c mice (375); Mouse mammary tumour explants (376); HUVECs and murine model (377) |
| oestrogen | otg | inr0 | ? | In vitro (378) |
| oestrogen | otg | inr1 | ? | BALB/c mice (375) |
| oestrogen | otg | gmo0 |  | Colon muscle cells (379) |
| oestrogen | otg | ocl1 |  | Human subjects (380) |
| oestrogen | otg | fty1 |  | Holtzman strain rats (381) |
| bone strength | ost | ocl1 |  | Normal human bone cells (382) |
| osteocalcin | ocl | ina1 |  | Mice (383); C57BL/6J mice (384) (385) |
| osteocalcin | ocl | ins1 |  | Mice (383); C57BL/6J mice (384) |
| osteocalcin | ocl | and1 |  | Mice (383); C57BL/6J mice (384) |
| osteocalcin | ocl | tet1 |  | 129-Sv mice (386); Mice (387) |
| osteocalcin | ocl | glp1 |  | STC-1 cells and C57BL/6J mice (388) |
| oxytocin | oxy | agr1 | ? | Wistar rats (389); Mice (390) Prairie voles (391) |
| oxytocin | oxy | agr0 | ? | Wild type Groningen rats (392) |
| oxytocin | oxy | cts1 | ? | Wistar rats (393) |
| oxytocin | oxy | cts0 | ? | Sprague-Dawley rats (394); Human subjects (395) |
| oxytocin | oxy | adp0 |  | Mice (396); Mice (397) |
| oxytocin | oxy | fdi0 |  | C57BL6 mice (398) |
| oxytocin | oxy | gng1 |  | Rat hepatocytes (57) |
| oxytocin | oxy | glg1 |  | Dogs (399) |
| oxytocin | oxy | noc0 |  | Sprague-Dawley rats (400) |
| plasma glucose | pgl | ins1 |  | Human subjects (401) |
| plasma glucose | pgl | ata1 |  | Human subjects and HUVECs (2) |
| plasma glucose | pgl | ghr0 |  | Human subjects (402) |
| plasma glucose | pgl | bgl1 |  | Human subjects (403) |
| plasma glucose | pgl | glg0 |  | Mongrel dogs (404) |
| plasma glucose | pgl | gt10 |  | Large White pigs (405); Rats (208) |
| brain glucose | bgl | cfn1 |  | Logic |
| brain glucose | bgl | fdi0 |  | C57BL/6NHsd mice (406); Rats (407) |
| sfrp5 | sfr | adp0 | ? | Mice (408) |
| sfrp5 | sfr | adp1 | ? | Mice (12) |
| sfrp5 | sfr | inr0 |  | Mice (408) |
| serotonin | ser | cfn1 |  | Mice (409); Human subjects (410); Human subjects (411); Rats (412); ICR mice (413); Marmoset monkeys (414); Wistar rats (415) |
| serotonin | ser | ina0 |  | Hamsters (358); C57BL/6 mice (416); Naïve rat hepatoma cells (417); Pigs (418) |
| serotonin | ser | ost0 |  | Mice (419); Human subjects (420); Human subjects (421); Mice (422) |
| serotonin | ser | agr0 |  | Human subjects (423); Rats (424); Mice (425); C57BL/6J mice (354); Human subjects (426); Human subjects (427); Dogs (428); Vervet monkeys (429); Human subjects (430); Dogs (431); Mice (432) |
| serotonin | ser | inr1 |  | C57BL/6 mice (433); Mice (434); HT-29 colon epithelial cells (435); Mice (436); C57BL/6J mice (437) |
| serotonin | ser | fdi0 |  | Wistar rats (438); Mice (439); Mice (440); Mice (441); Human subjects (442) |
| serotonin | ser | dop0 |  | Rats (443) |
| symapathetic stimulation | sys | gng1 |  | Rats (206) |
| symapathetic stimulation | sys | ina1 |  | Human subjects (444) |
| symapathetic stimulation | sys | adp0 |  | Sprague-Dawley rat (445); Human subjects (446) |
| sympathetic stimulation | sys | egf1 |  | Human subjects (447) |
| testosterone | tet | edp0 |  | Rats (448) |
| testosterone | tet | msh0 |  | Rats (448) |
| testosterone | tet | egf1 |  | Mice (449); Mice (145) |
| testosterone | tet | myo0 |  | C57BL6J mice (450) |
| testosterone | tet | msl1 |  | Human subjects (451); Human subjects (452) |
| testosterone | tet | ocl1 |  | Human subjects (380) |
| testosterone | tet | adp0 |  | Human subjects (453); Human subjects (454); Rats (455); Human subjects (456); Human subjects (457) |
| testosterone | tet | dip0 |  | Logic |
| testosterone | tet | aox1 |  | Cerebellar granule cells (458) |
| testosterone | tet | epo1 |  | Human subjects (459) |
| testosterone | tet | agr1 |  | Mice (355); Rats (367); Rats (368); CD-1 mice (370) |
| testosterone | tet | ina1 | ? | Human subjects (457); Human subjects (453) |
| testosterone | tet | ina0 | ? | Rat skeletal muscle culture (460) |
| testosterone | tet | ang1 |  | Sprague-Dawley rats (461) |
| TNF alpha | tnf | inr1 |  | Mice (462) |
| TNF alpha | tnf | ina0 |  | Murine 3T3-L1 or 3T3-F442A cells (463); Human adipose tissue (249) |
| TNF alpha | tnf | ata1 |  | Human leucocytes (464); C57BL6/J mice (465) |
| TNF alpha | tnf | il61 |  | SCID-HuRAg mice (466); LS14 cell culture (467) |
| TNF alpha | tnf | lep1 |  | C57BL/6J mice (468); Human subjects (469); C3H/HeOuJ mice (470); Syrian hamsters (120); C57BL/6 mice (121) |
| TNF alpha | tnf | klt0 |  | C57/BL6 mice (125); Mice and mouse embryonic adipocytes (471) |
| TNF alpha | tnf | et11 |  | Bovine aortic endothelial cells (472) |
| TNF alpha | tnf | nep0 |  | Rats (473) |
| triglycerides | tri | adp1 |  | C57BL6 mice(474) |
| triglycerides | tri | ina0 |  | C57BL6 mice(474) |
| vasodilation | vdl | bgl1 |  | Logic |
| vasodilation | vdl | ina1 |  | Sprague-Dawley rats (475) |
| vitamin D3 | vd3 | ina1 |  | Human subjects (476); Rats (477) |
| vitamin D3 | vd3 | ost1 |  | Human subjects (478) |
| vitamin D3 | vd3 | agr1 |  | Mice (479) |
| vitamin D3 | vd3 | ins1 |  | Rat pancreas (480); Rats (477) |
| Vitamin B12 | v12 | igf1 |  | Mice (223) |
| Vitamin B12 | v12 | inr0 |  | Mice (481) |
| Vitamin B12 | v12 | hgh1 |  | Mice (223) |
| Vitamin B12 | v12 | ost1 |  | Mice (223) |
| Vitamin B12 | v12 | noc0 |  | Mice (481) |
| Exercise | exe | adp0 |  | OM and S5B/P1 rats (482) |
| Exercise | exe | il61 |  | Humans(483) |
| GLP-1 | glp | ins1 |  | Rat pancreatic ductal cells (484); Human subjects (485) |
| GLP-1 | glp | btc1 |  | Human islets (486); Rat pancreatic ductal cells (484) |
| GLP-1 | glp | glg0 |  | Wistar rats (487) |
| GLP-1 | glp | gmo0 |  | Human subjects (488) |
| GLP-1 | glp | fdi0 |  | Human subjects (488) |
| GLP-1 | glp | ina1 |  | Human subjects (488) |
| GLP-1 | glp | ost1 |  | Human subjects (489); Sprague-Dawley rats (490) |
| Gut Motility | gmo | glp1 |  | Logic |
| Nociception | noc | ina0 |  | Human subjects (491) |

**Supporting references**

1. Li L, Yi Z, Seno M, Kojima I. Activin A and Betacellulin. Effect on Regeneration of Pancreatic Beta-Cells in Neonatal Streptozotocin-Treated Rats. Diabetes [Internet]. 2004;53(March):608–15. Available from: http://diabetes.diabetesjournals.org/content/53/3/608.full.pdf

2. Andersen GØ, Ueland T, Knudsen EC, Scholz H, Yndestad A, Sahraoui A, et al. Activin A levels are associated with abnormal glucose regulation in patients with myocardial infarction: potential counteracting effects of activin A on inflammation. Diabetes [Internet]. 2011 May [cited 2013 Jan 10];60(5):1544–51. Available from: http://www.pubmedcentral.nih.gov/articlerender.fcgi?artid=3292329&tool=pmcentrez&rendertype=abstract

3. Florio P, Luisi S, Marchetti P, Lupi R, Cobellis L, Falaschi C, et al. Activin A stimulates insulin secretion in cultured human pancreatic islets. J Endrocrinological Investig [Internet]. 2000;23(4):231–4. Available from: http://www.ncbi.nlm.nih.gov/pubmed/10853708

4. Zaragosi L, Wdziekonski B, Villageois P, Keophiphath M, Maumus M, Tchkonia T, et al. Activin A Plays a Critical Role in Proliferation and Differentiation of Human Adipose Progenitors. Diabetes [Internet]. 2010;59(October):2513–21. Available from: http://diabetes.diabetesjournals.org/content/59/10/2513.full.pdf

5. Hotamisligil GS, Arner P, Caro JF, Atkinson RL, Spiegelman BM. Increased Adipose Tissue Expression of Tumor Necrosis Factor-alpha in Human Obesity and Insulin Resistance. J Clin Invest [Internet]. 1995;95(January):2409–15. Available from: http://www.ncbi.nlm.nih.gov/pmc/articles/PMC295872/pdf/jcinvest00026-0467.pdf

6. Eriksson P, Reynisdottir S, Lönnqvist F, Stemme V, Hamsten A, Arner P. Adipose tissue secretion of plasminogen activator inhibitor-1 in non-obese and obese individuals. Diabetologia [Internet]. 1998;41:65–71. Available from: http://link.springer.com/content/pdf

7. Maeda K, Okubo K, Shimomura I, Funahashi T, Matsuzawa Y, Matsubara K. cDNA cloning and expression of a novel adipose specific collagen-like factor, apM1 (adipose most abundant gene transcript 1). 1996. Biochem Biophys Res Commun [Internet]. 1996 Aug 31;221(2):286–9. Available from: http://www.ncbi.nlm.nih.gov/pubmed/22925673

8. Van Harmelen V, Reynisdottir S, Eriksson P, Thörne A, Hoffstedt J, Lönnqvist F, et al. Leptin secretion from subcutaneous and visceral adipose tissue in women. Diabetes [Internet]. 1998 Jun;47(6):913–7. Available from: http://www.ncbi.nlm.nih.gov/pubmed/12161541

9. Barr VA, Malide D, Zarnowski MJ, Taylor SI, Cushman SW. Insulin stimulates both leptin secretion and production by rat white adipose tissue. Endocrinology [Internet]. 1997 Oct;138(10):4463–72. Available from: http://www.ncbi.nlm.nih.gov/pubmed/9322964

10. Curat CA, Miranville A, Sengene C, Diehl M, Tonus C, Busse R, et al. From Blood Monocytes to Adipose Tissue–Resident Macrophages. Diabetes [Internet]. 2004;53(May):1285–92. Available from: http://diabetes.diabetesjournals.org/content/53/5/1285.full.pdf

11. Evans BA, Agar L, Summers RJ. The role of the sympathetic nervous system in the regulation of leptin synthesis in C57BL/6 mice. FEBS Lett [Internet]. 1999 Feb 12;444(2–3):149–54. Available from: http://www.ncbi.nlm.nih.gov/pubmed/10050748

12. Mori H, Prestwich TC, Reid MA, Longo KA, Gerin I, Cawthorn WP, et al. Secreted frizzled-related protein 5 suppresses adipocyte mitochondrial metabolism through WNT inhibition. J Clin Invest [Internet]. 2012;122(7):2405–16. Available from: http://www.jci.org/articles/view/63604/pdf

13. Ouchi N, Higuchi A, Ohashi K, Oshima Y, Gokce N, Shibata R, et al. Sfrp5 is an anti-inflammatory adipokine that modulates metabolic dysfunction in obesity. Science (80- ) [Internet]. 2011;329(5990):454–7. Available from: http://www.ncbi.nlm.nih.gov/pmc/articles/PMC3132938/pdf/nihms-308580.pdf

14. Yamauchi T, Kamon J, Waki H, Terauchi Y, Kubota N, Hara K, et al. The fat-derived hormone adiponectin reverses insulin resistance associated with both lipoatrophy and obesity. Nat Med [Internet]. 2001;7(8):941–6. Available from: http://www.ncbi.nlm.nih.gov/pubmed/11479627

15. Kubota N, Terauchi Y, Yamauchi T, Kubota T, Moroi M, Matsui J, et al. Disruption of adiponectin causes insulin resistance and neointimal formation. J Biol Chem [Internet]. 2002 Jul 19 [cited 2012 Nov 19];277(29):25863–6. Available from: http://www.ncbi.nlm.nih.gov/pubmed/12032136

16. Weigert J, Neumeier M, Wanninger J, Schober F, Sporrer D, Weber M, et al. Adiponectin upregulates monocytic activin A but systemic levels are not altered in obesity or type 2 diabetes. Diabetes [Internet]. Elsevier Ltd; 2009 Feb [cited 2013 Jan 10];45(2):86–91. Available from: http://www.ncbi.nlm.nih.gov/pubmed/19128983

17. Fruebis J, Tsao T-S, Javorschi S, Ebbets-Reed D, Erickson MRS, Yen FT, et al. Proteolytic cleavage product of 30-kDa adipocyte complement-related protein increases fatty acid oxidation in muscle and causes weight loss in mice. Proc Natl Acad Sci U S A [Internet]. 2001 Feb 13;98(4):2005–10. Available from: http://www.pubmedcentral.nih.gov/articlerender.fcgi?artid=29372&tool=pmcentrez&rendertype=abstract

18. Tambascia M, Coope A, Milanski M, Arau EP, Saad JA, Geloneze B. AdipoR1 mediates the anorexigenic and insulin / leptin-like actions of adiponectin in the hypothalamus. Fed Eur Biochem Soc Lett [Internet]. 2008;582(10):1471–6. Available from: http://www.ncbi.nlm.nih.gov/pubmed/18394428

19. Ouchi N, Kihara S, Arita Y, Okamoto Y, Maeda K, Kuriyama H, et al. Adiponectin, an Adipocyte-Derived Plasma Protein, Inhibits Endothelial NF- B Signaling Through a cAMP-Dependent Pathway. Circulation [Internet]. 2000 Sep 12 [cited 2013 Jan 10];102(11):1296–301. Available from: http://circ.ahajournals.org/cgi/doi/10.1161/01.CIR.102.11.1296

20. Maillard V, Uzbekova S, Guignot F, Perreau C, Ramé C, Coyral-Castel S, et al. Effect of adiponectin on bovine granulosa cell steroidogenesis, oocyte maturation and embryo development. Reprod Biol Endocrinol [Internet]. 2010 Jan;8:23. Available from: http://www.pubmedcentral.nih.gov/articlerender.fcgi?artid=2845137&tool=pmcentrez&rendertype=abstract

21. Čikoš Š, Burkuš J, Bukovská A, Fabian D, Rehák P, Koppel J. Expression of adiponectin receptors and effects of adiponectin isoforms in mouse preimplantation embryos. Hum Reprod [Internet]. 2010 Sep [cited 2013 Feb 21];25(9):2247–55. Available from: http://www.ncbi.nlm.nih.gov/pubmed/20663797

22. Richards JS, Liu Z, Kawai T, Tabata K, Watanabe H, Suresh D, et al. Adiponectin and its receptors modulate granulosa cell and cumulus cell functions, fertility, and early embryo development in the mouse and human. Fertil Steril [Internet]. 2012;98(2):471–9. Available from: http://www.ncbi.nlm.nih.gov/pubmed/22633650

23. Chatterjee P, Banerjee A, Majumdar P. Study of plasma lipid and lipoprotein profile in elite women boxers during a six weeks’ training progamme. J Nepal Med Assoc [Internet]. 2007;46(165):25–30. Available from: http://www.ncbi.nlm.nih.gov/pubmed/17721559

24. Nexø E, Hollenberg M, Bing J. Aggressive behavior in mice provokes a marked increase in both plasma epidermal growth factor and renin. Acta Physiol Scand [Internet]. 1981;111(3):367–71. Available from: http://www.ncbi.nlm.nih.gov/pubmed/7032211

25. Sánchez O, Viladrich M, Ramírez I, Soley M. Liver injury after an aggressive encounter in male mice. Am J Physiol Regul Integr Comp Physiol [Internet]. 2007 Nov [cited 2013 Feb 5];293(5):R1908-16. Available from: http://www.ncbi.nlm.nih.gov/pubmed/17761516

26. Nexø E, Olsen P, Poulsen K. Exocrine and endocrine secretion of renin and epidermal growth factor from the mouse submandibular glands. Regul Pept [Internet]. 1984;8(4):327–34. Available from: http://www.ncbi.nlm.nih.gov/pubmed/6387816

27. Spillantini MG, Aloe L, Alleva E, De Simone R, Goedert M, Levi-Montalcini R. Nerve growth factor mRNA and protein increase in hypothalamus in a mouse model of aggression. Proc Natl Acad Sci U S A [Internet]. 1989 Nov;86(21):8555–9. Available from: http://www.pubmedcentral.nih.gov/articlerender.fcgi?artid=298321&tool=pmcentrez&rendertype=abstract

28. Maestripieri D, De Simone R, Aloe L, Alleva E. Social status and nerve growth factor serum levels after agonistic encounters in mice. Physiol Behav [Internet]. 1990;47(1):161–4. Available from: http://www.ncbi.nlm.nih.gov/pubmed/2326332

29. Lakshmanan J. Aggressive behavior in adult male mice elevates serum nerve growth factor levels. Am J Physiol [Internet]. 1986;250(4.1):E386-92. Available from: http://www.ncbi.nlm.nih.gov/pubmed/3515963

30. Lakshmanan J. Beta-nerve growth factor measurements in mouse serum. J Neurochem [Internet]. 1986;46(3):882–91. Available from: http://www.ncbi.nlm.nih.gov/pubmed/3950612

31. Lakshmanan J. Nerve growth factor levels in mouse serum: variations due to stress. Neurochem Res [Internet]. 1987;12(4):393–7. Available from: http://www.ncbi.nlm.nih.gov/pubmed/3600964

32. Stacie LT, Stanek LM, Ressler KJ, Huhman KL. Differential BDNF expression in limbic brain regions following social defeat or territorial aggression. Behav Neurosci [Internet]. 2012;125(6):911–20. Available from: http://www.ncbi.nlm.nih.gov/pmc/articles/PMC3232038/

33. Ferrari P, van Erp A, Tornatzky W, Miczek K. Accumbal dopamine and serotonin in anticipation of the next aggressive episode in rats. Eur J Neurosci [Internet]. 2003;17(2):371–8. Available from: http://www.ncbi.nlm.nih.gov/pubmed/12542674

34. Erp AMM Van, Miczek KA. Aggressive Behavior, Increased Accumbal Dopamine, and Decreased Cortical Serotonin in Rats. J Neurosci [Internet]. 2000;20(24):9320–5. Available from: http://www.jneurosci.org/content/20/24/9320.full.pdf

35. Farrell P, Kjaer M, Bach F, Galbo H. Beta-endorphin and adrenocorticotropin response to supramaximal treadmill exercise in trained and untrained males. Acta Physiol Scand [Internet]. 1987;130(4):619–25. Available from: http://www.ncbi.nlm.nih.gov/pubmed/2820200

36. Schwarz L, Kindermann W. Beta-endorphin, catecholamines, and cortisol during exhaustive endurance exercise. Int J Sports Med [Internet]. 1989;10(5):324–8. Available from: http://www.ncbi.nlm.nih.gov/pubmed/2532179

37. Elias M. Serum cortisol, testosterone, and testosterone-binding globulin responses to competitive fighting in human males. Aggress Behav [Internet]. 1981;7(3):215–24. Available from: http://onlinelibrary.wiley.com/doi/10.1002/1098-2337(1981)7:3%3C215::AID-AB2480070305%3E3.0.CO;2-M/abstract

38. Burgdorf J, Panksepp J, Beinfeld MC, Kroes RA, Moskal JR. Regional brain cholecystokinin changes as a function of rough-and-tumble play behavior in adolescent rats. Peptides [Internet]. 2006;27(1):172–7. Available from: http://www.sciencedirect.com/science/article/pii/S0196978105003475

39. Kurihara Y, Kurihara H, Morita H, Cao W-H, Ling G-Y, Kumada M, et al. Role of endothelin-1 in stress response in the central nervous system. Am J Physiol - Regul Integr Comp Physiol [Internet]. 2000;279(2):R515-21. Available from: http://ajpregu.physiology.org/content/279/2/R515.full.pdf

40. Bartos L, Reyes E, Schams D, Bubenik G, Lobos A. Rank dependent seasonal levels of IGF-1 , cortisol and reproductive hormones in male pudu ( Pudu puda ). Comp Biochem Physiol A Mol Integr Physiol [Internet]. 1998;120(2):373–8. Available from: http://www.ncbi.nlm.nih.gov/pubmed/9773515

41. Sapolsky RM, Spencer EM. Insulin-like growth factor I is suppressed in socially subordinate male baboons. Am J Physiol [Internet]. 1997;273(4.2):1346–51. Available from: http://www.ncbi.nlm.nih.gov/pubmed/9362298

42. Banno R, Arima H, Sato I, Hayashi M, Goto M, Sugimura Y, et al. The melanocortin agonist melanotan II increases insulin sensitivity in OLETF rats. Peptides [Internet]. 2004 Aug [cited 2013 Jan 15];25(8):1279–86. Available from: http://www.ncbi.nlm.nih.gov/pubmed/15350695

43. Obici S, Feng Z, Tan J, Liu L, Karkanias G, Rossetti L. Central melanocortin receptors regulate insulin action. J Clin Invest [Internet]. 2001;108(7):1079–85. Available from: http://static.jci.org/content_assets/manuscripts/12000/12954/JCI0112954.pdf

44. Nowell N, Thody A, Woodley R. The source of an aggression-promoting olfactory cue, released by alpha-melanocyte stimulating hormone, in the male mouse. Peptides [Internet]. 1980;1(1):69–72. Available from: http://www.ncbi.nlm.nih.gov/pubmed/7195571

45. Gonzalez MI, Vaziri S, Wilson CA. Behavioral effects of alpha-MSH and MCH after central administration in the female rat. Peptides [Internet]. 1996 Jan;17(1):171–7. Available from: http://www.ncbi.nlm.nih.gov/pubmed/8822527

46. Morgan C, Thomas RE, Cone RD. Melanocortin-5 receptor deficiency promotes defensive behavior in male mice. Horm Behav [Internet]. 2004 Jan [cited 2013 Feb 21];45(1):58–63. Available from: http://linkinghub.elsevier.com/retrieve/pii/S0018506X03001776

47. Gómez-sanMiguel AB, Martín AI, Nieto-bona MP, Fernández-galaz C, López-menduiña M, Villanúa MÁ, et al. Systemic alpha-melanocyte-stimulating hormone administration decreases arthritis-induced anorexia and muscle wasting. Am J Physiol - Regul Integr Comp Physiol [Internet]. 2013;304:R877–86. Available from: http://ajpregu.physiology.org/content/early/2013/03/15/ajpregu.00447.2012

48. Catania A, Cutuli M, Garofalo L, Airaghi L, Valenza F, Lipton J, et al. Plasma concentrations and anti-L-cytokine effects of alpha-melanocyte stimulating hormone in septic patients. Crit Care Med [Internet]. 2000;28(5):1403–7. Available from: http://www.ncbi.nlm.nih.gov/pubmed/10834686

49. McMinn JE, Wilkinson CW, Havel P, Woods SC, Schwartz MW. Effect of intracerebroventricular alpha-MSH on food intake, adiposity, c-Fos induction, and neuropeptide expression. Am J Physiol Regul Integr Comp Physiol [Internet]. 2000 Aug;279(2):R695-703. Available from: http://www.ncbi.nlm.nih.gov/pubmed/10938261

50. Dutia R, Meece K, Dighe S, Kim AJ, Wardlaw SL. β-Endorphin antagonizes the effects of α-MSH on food intake and body weight. Endocrinology [Internet]. 2012;153(9):4246–55. Available from: http://www.ncbi.nlm.nih.gov/pubmed/22778225

51. Furukawa S, Fujita T, Shimabukuro M, Iwaki M, Yamada Y, Nakajima Y, et al. Increased oxidative stress in obesity and its impact on metabolic syndrome. J Clin Invest [Internet]. 2004;114(12):1752–61. Available from: http://www.ncbi.nlm.nih.gov/pmc/articles/PMC535065/pdf/JCI0421625.pdf

52. Ferris C, Delville Y. Vasopressin and serotonin interactions in the control of agonistic behavior. Psychoneuroendocrinology [Internet]. 1994;19(5–7):593–601. Available from: http://www.ncbi.nlm.nih.gov/pubmed/7938357

53. Dons R, House J, Hood D, Krehbiel M. Assessment of desmopressin-enhanced cognitive function in a neurosurgical patient. Mil Med [Internet]. 1989;154(2):83–5. Available from: http://www.ncbi.nlm.nih.gov/pubmed/2494586

54. Beckwith BE, Till RE, Schneider V. Vasopressin analog (DDAVP) improves memory in human males. Peptides [Internet]. 1984;5(4):819–22. Available from: http://www.sciencedirect.com/science/article/pii/0196978184900287

55. Gold P, Ballenger JC, Weingartner H, Goodwin F, Post RM. Effects Of 1-Desamo-8-D-Arginine Vasopressin on behaviour and cognition in primary affective disorder. Lancet [Internet]. 1979;314(8150):992–4. Available from: http://www.sciencedirect.com/science/article/pii/S0140673679925637

56. O’Carroll A-M, Howell GM, Roberts EM, Lolait SJ. Vasopressin potentiates corticotropin-releasing hormone-induced insulin release from mouse pancreatic beta-cells. J Endocrinol [Internet]. 2008 May [cited 2013 Feb 27];197(2):231–9. Available from: http://www.pubmedcentral.nih.gov/articlerender.fcgi?artid=2386661&tool=pmcentrez&rendertype=abstract

57. Whitton PD, Rodrigues LM, Hems DA. Stimulation by vasopressin, angiotensin and oxytocin of Gluconeogenesis in Hepatocyte Suspensions. Biochem J [Internet]. 1978;176(3):893–8. Available from: http://www.ncbi.nlm.nih.gov/pmc/articles/PMC1186313/pdf/biochemj00472-0255.pdf

58. Yamanaka M, Itakura Y, Inoue T, Tsuchida A, Nakagawa T, Noguchi H, et al. Protective effect of brain-derived neurotrophic factor on pancreatic islets in obese diabetic mice. Metabolism [Internet]. 2006 Oct [cited 2012 Nov 23];55(10):1286–92. Available from: http://www.ncbi.nlm.nih.gov/pubmed/16979397

59. Duan W, Guo Z, Jiang H, Ware M, Mattson MP. Reversal of Behavioral and Metabolic Abnormalities, and Insulin Resistance Syndrome, by Dietary Restriction in Mice Deficient in Brain-Derived Neurotrophic Factor. Endocrinology [Internet]. 2003 Jun 1 [cited 2012 Nov 16];144(6):2446–53. Available from: http://endo.endojournals.org/cgi/doi/10.1210/en.2002-0113

60. Kuroda A, Yamasaki Y, Matsuhisa M, Kubota M, Nakahara I, Nakatani Y, et al. Brain-derived neurotrophic factor ameliorates hepatic insulin resistance in Zucker fatty rats. Metabolism [Internet]. 2003 Feb [cited 2013 Jan 18];52(2):203–8. Available from: http://www.ncbi.nlm.nih.gov/pubmed/12601633

61. Nakagawa T, Tsuchida A, Itakura Y, Nonomura T, Ono M, Hirota F, et al. Brain-derived neurotrophic factor regulates glucose metabolism by modulating energy balance in diabetic mice. Diabetes [Internet]. 2000 Mar;49(3):436–44. Available from: http://www.ncbi.nlm.nih.gov/pubmed/10868966

62. Gray J, Yeo GSH, Cox JJ, Morton J, Adlam A-LR, Keogh JM, et al. Hyperphagia, severe obesity, impaired cognitive function, and hyperactivity associated with functional loss of one copy of the brain-derived neurotrophic factor (BDNF) gene. Diabetes [Internet]. 2006 Dec [cited 2013 Feb 20];55(12):3366–71. Available from: http://www.pubmedcentral.nih.gov/articlerender.fcgi?artid=2413291&tool=pmcentrez&rendertype=abstract

63. Lyons WE, Mamounas LA, Ricaurte GA, Coppola V, Reid SW, Bora SH, et al. Brain-derived neurotrophic factor-deficient mice develop aggressiveness and hyperphagia in conjunction with brain serotonergic abnormalities. Proc Natl Acad Sci U S A [Internet]. 1999 Dec 21;96(26):15239–44. Available from: http://www.pubmedcentral.nih.gov/articlerender.fcgi?artid=24804&tool=pmcentrez&rendertype=abstract

64. Cao L, Lin E-JD, Cahill MC, Wang C, Liu X, During MJ. Molecular therapy of obesity and diabetes by a physiological autoregulatory approach. Nat Med [Internet]. 2009 Apr [cited 2013 Sep 25];15(4):447–54. Available from: http://www.ncbi.nlm.nih.gov/pubmed/19270710

65. Zilberfarb V, Piétri-Rouxel F, Jockers R, Krief S, Delouis C, Issad T, et al. Human immortalized brown adipocytes express functional β 3 -adrenoceptor coupled to lipolysis. J Cell Sci [Internet]. 1997 Apr;110:801–7. Available from: http://www.ncbi.nlm.nih.gov/pubmed/9133667

66. Champigny O, Ricquier D, Blondel O, Mayers RM, Briscoe MG, Holloway BR. Beta 3-Adrenergic receptor stimulation restores message and expression of brown-fat mitochondrial uncoupling protein in adult dogs. Proc Natl Acad Sci U S A [Internet]. 1991;88(December):10774–7. Available from: http://www.pnas.org/content/88/23/10774.long

67. Michael DJ, Ritzel RA, Haataja L, Chow RH. Pancreatic Beta-Cells Secrete Insulin in Fast- and Slow-Release Forms. Diabetes [Internet]. 2006;55(March):600–7. Available from: http://diabetes.diabetesjournals.org/content/55/3/600.full.pdf

68. Taniguchi H, Okada Y, Seguchi H, Shimada C, Seki M, Tsutou A, et al. High concentration of gamma-aminobutyric acid in pancreatic beta cells. Diabetes [Internet]. 1979;28(7):629–33. Available from: http://diabetes.diabetesjournals.org/content/28/7/629.full-text.pdf

69. Asnicar MA, Smith DP, Yang DD, Heiman ML, Fox N, Chen Y, et al. Absence of Cocaine- and Amphetamine-Regulated Transcript Results in Obesity in Mice Fed a High Caloric Diet. Endocrinology [Internet]. 2001;142(10):4394–400. Available from: http://endo.endojournals.org/content/142/10/4394.full.pdf

70. Kristensen P, Judge ME, Thim L, Ribel U, Christjansen KN, Wulff BS, et al. Hypothalamic CART is a new anorectic peptide regulated by leptin. Nature [Internet]. 1998;393(May):72–6. Available from: http://www.nature.com/nature/journal/v393/n6680/pdf/393072a0.pdf

71. Kopin AS, Mathes WF, McBride EW, Nguyen M, Al-Haider W, Schmitz F, et al. The cholecystokinin-A receptor mediates inhibition of food intake yet is not essential for the maintenance of body weight. J Clin Invest [Internet]. 1999 Feb;103(3):383–91. Available from: http://www.pubmedcentral.nih.gov/articlerender.fcgi?artid=407901&tool=pmcentrez&rendertype=abstract

72. Kissileff HR, Pi-Sunyer FX, Thornton J, Smith GP. C-terminal octapeptide of cholecystokinin decreases food intake in man. Am J Clin Nutr [Internet]. 1981;34(February):154–60. Available from: http://ajcn.nutrition.org/content/34/2/154.full.pdf

73. Moran TH, Katz LF, Plata-salaman CR, Schwartz GJ. Disordered food intake and obesity in rats lacking cholecystokinin A receptors. Am J Physiol - Regul Integr Comp Physiol [Internet]. 1998;274:R618-25. Available from: http://ajpregu.physiology.org/content/274/3/R618.full.pdf

74. Gibbs J, Young RC, Smith GP. Cholecystokinin decreases food intake in rats. Obes Res [Internet]. 1997 May;5(3):284–90. Available from: http://www.ncbi.nlm.nih.gov/pubmed/9192405

75. Gibbs J, Falasco L, McHugh P. Cholecystokinin-decreased food intake in rhesus monkeys. Am J Physiol [Internet]. 1976;230(1):15–8. Available from: http://www.ncbi.nlm.nih.gov/pubmed/814821

76. Stein LJ, Woods SC. Cholecystokinin and bombesin act independently to decrease food intake in the rat. Peptides [Internet]. 1981;2(4):431–6. Available from: http://www.sciencedirect.com/science/article/pii/S0196978181801003

77. Cannon CM, Palmiter RD. Peptides that regulate food intake: norepinephrine is not required for reduction of feeding induced by cholecystokinin. Am J Physiol Regul Integr Comp Physiol [Internet]. 2003 Jun [cited 2013 Feb 13];284(6):R1384-8. Available from: http://www.ncbi.nlm.nih.gov/pubmed/12736175

78. Irwin N, Frizelle P, Montgomery IA, Moffett RC, O’Harte FPM, Flatt PR. Beneficial effects of the novel cholecystokinin agonist ( pGlu-Gln ) -CCK-8 in mouse models of obesity / diabetes. Diabetologia [Internet]. 2012;55(10):2747–58. Available from: http://www.ncbi.nlm.nih.gov/pubmed/22814764

79. Schmidt P, Hansen L, Hilsted L, Holst J. Cholecystokinin inhibits gastrin secretion independently of paracrine somatostatin secretion in the pig. Scand J Gastroenterol [Internet]. 2004;39(3):217–21. Available from: http://www.ncbi.nlm.nih.gov/pubmed/15074389

80. Ahrén B, Holst JJ, Efendic S. Antidiabetogenic action of cholecystokinin-8 in type 2 diabetes. J Clin Endocrinol Metab [Internet]. 2000 Mar;85(3):1043–8. Available from: http://www.ncbi.nlm.nih.gov/pubmed/10720037

81. Kaplan JR, Shively CA, Fontenot MB, Morgan TM, Howell SM, Manuck SB, et al. Demonstration of an Association Among Dietary Cholesterol , Central Serotonergic Activity , and Social Behavior in Monkeys. Psychosom Med [Internet]. 1994;56(6):479–84. Available from: http://www.psychosomaticmedicine.org/content/56/6/479.long

82. Subramanian S, Han CY, Chiba T, McMillen TS, Wang S a, Haw A, et al. Dietary cholesterol worsens adipose tissue macrophage accumulation and atherosclerosis in obese LDL receptor-deficient mice. Arterioscler Thromb Vasc Biol [Internet]. 2008 Apr [cited 2013 Jan 10];28(4):685–91. Available from: http://www.pubmedcentral.nih.gov/articlerender.fcgi?artid=2767166&tool=pmcentrez&rendertype=abstract

83. Kaplan JR, Manuck SB, Shively C. The Effects of Fat and Cholesterol on Social Behavior in Monkeys. Psychosom Med [Internet]. 1991;53(6):634–42. Available from: http://www.psychosomaticmedicine.org/content/53/6/634.long

84. Muldoon M, Ryan C, Sereika S, Flory J, Manuck S. Randomized trial of the effects of simvastatin on cognitive functioning in hypercholesterolemic adults. Am J Med [Internet]. 2004;117(11):823–9. Available from: http://www.ncbi.nlm.nih.gov/pubmed/15589485?dopt=Abstract

85. Campbell JE, Peckett AJ, D’souza AM, Hawke TJ, Riddell MC. Adipogenic and lipolytic effects of chronic glucocorticoid exposure. Am J Physiol Cell Physiol [Internet]. 2011 Jan [cited 2013 Jan 30];300(1):C198-209. Available from: http://www.ncbi.nlm.nih.gov/pubmed/20943959

86. Djurhuus CB, Gravholt CH, Nielsen S, Mengel a, Christiansen JS, Schmitz OE, et al. Effects of cortisol on lipolysis and regional interstitial glycerol levels in humans. Am J Physiol Endocrinol Metab [Internet]. 2002 Jul [cited 2013 Feb 9];283(1):E172-7. Available from: http://www.ncbi.nlm.nih.gov/pubmed/12067858

87. Schmoll D, Wasner C, Hinds CJ, Allan BB, Walther R, Burchell A. Identiﬁcation of a cAMP response element within the glucose-6-phosphatase hydrolytic subunit gene promoter which is involved in the transcriptional regulation by cAMP and glucocorticoids in H4IIE hepatoma cells. Biochem J [Internet]. 1999;338:457–63. Available from: http://www.biochemj.org/bj/338/0457/3380457.pdf

88. Petersen DD, Magnuson MA, Granner DK. Location and characterization of two widely separated glucocorticoid response elements in the phosphoenolpyruvate carboxykinase gene. Mol Cell Biol [Internet]. 1988 Jan;8(1):96–104. Available from: http://www.pubmedcentral.nih.gov/articlerender.fcgi?artid=363086&tool=pmcentrez&rendertype=abstract

89. Yamada K, Duong DT, Scott DK, Wang JC, Granner DK. CCAAT/enhancer-binding protein beta is an accessory factor for the glucocorticoid response from the cAMP response element in the rat phosphoenolpyruvate carboxykinase gene promoter. J Biol Chem [Internet]. 1999 Feb 26;274(9):5880–7. Available from: http://www.ncbi.nlm.nih.gov/pubmed/10026211

90. Kusunoki M, Cooney G, Hara T, Storlien L. Amelioration of high-fat feeding-induced insulin resistance in skeletal muscle with the antiglucocorticoid RU486. Diabetes [Internet]. 1995;44(6):718–20. Available from: http://www.ncbi.nlm.nih.gov/pubmed/7789638

91. Freedman M, Stern J, Reaven G, Mondon C. Effect of adrenalectomy on in vivo glucose metabolism in insulin resistant Zucker obese rats. Horm Metab Res [Internet]. 1986;18(5):296–8. Available from: http://www.ncbi.nlm.nih.gov/pubmed/3522389

92. Ruzzin J, Wagman a S, Jensen J. Glucocorticoid-induced insulin resistance in skeletal muscles: defects in insulin signalling and the effects of a selective glycogen synthase kinase-3 inhibitor. Diabetologia [Internet]. 2005 Oct [cited 2012 Nov 28];48(10):2119–30. Available from: http://www.ncbi.nlm.nih.gov/pubmed/16078016

93. Masuzaki H, Paterson J, Shinyama H, Morton NM, Mullins JJ, Seckl JR, et al. A transgenic model of visceral obesity and the metabolic syndrome. Science (80- ) [Internet]. 2001 Dec 7 [cited 2012 Oct 27];294(5549):2166–70. Available from: http://www.ncbi.nlm.nih.gov/pubmed/11739957

94. Midtvedt K, Hjelmesaeth J, Hartmann A, Lund K, Paulsen D, Egeland T, et al. Insulin resistance after renal transplantation: The effect of steroid dose reduction and withdrawal. J Am Soc Nephrol [Internet]. 2004 Dec [cited 2012 Dec 9];15(12):3233–9. Available from: http://www.ncbi.nlm.nih.gov/pubmed/15579527

95. Ogura M, Kusaka I, Nagasaka S, Yatagai T, Shinozaki S, Itabashi N, et al. Unilateral adrenalectomy improves insulin resistance and diabetes mellitus in a patient with ACTH-independent macronodular adrenal hyperplasia. Endocr J [Internet]. 2003 Dec;50(6):715–21. Available from: http://www.ncbi.nlm.nih.gov/pubmed/14709842

96. Rizza R, Mandarino L, Gerich J. Cortisol-induced insulin resistance in man: impaired suppression of glucose production and stimulation of glucose utilization due to a postreceptor detect of insulin action. J Clin Endocrinol Metab [Internet]. 1982;54(1):131–8. Available from: http://www.ncbi.nlm.nih.gov/pubmed/7033265

97. Christiansen JJ, Djurhuus CB, Gravholt CH, Iversen P, Christiansen JS, Schmitz O, et al. Effects of cortisol on carbohydrate, lipid, and protein metabolism: Studies of acute cortisol withdrawal in adrenocortical failure. J Clin Endocrinol Metab [Internet]. 2007 Sep [cited 2013 Mar 1];92(9):3553–9. Available from: http://www.ncbi.nlm.nih.gov/pubmed/17609300

98. Leshner AI, Korn SJ, Mixon JF, Rosenthal C, Besser AK. Effects of Corticosterone on Submissiveness in Mice : Some Temporal and Theoretical. Physiol Behav [Internet]. 1980;24:283–8. Available from: http://www.sciencedirect.com/science/article/pii/0031938480900876

99. Yildiz F, Kaur AC, A. I, Çelikogˇlu M, Özkara SK, Paksoy N, et al. Inhaled Corticosteroids May Reduce Neutrophilic Inflammation in Patients with Stable Chronic Obstructive Pulmonary Disease. Respiration [Internet]. 2000;67(1):71–6. Available from: http://content.karger.com/ProdukteDB/produkte.asp?Aktion=ShowPDF&ArtikelNr=29466&Ausgabe=226558&ProduktNr=224278&filename=29466.pdf

100. Patel IS, Roberts NJ, Lloyd-Owen SJ, Sapsford RJ, Wedzicha J a. Airway epithelial inflammatory responses and clinical parameters in COPD. Eur Respir J [Internet]. 2003 Jul 1 [cited 2013 Jan 22];22(1):94–9. Available from: http://erj.ersjournals.com/cgi/doi/10.1183/09031936.03.00093703

101. Pinto-Plata VM, Müllerova H, Toso JF, Feudjo-Tepie M, Soriano JB, Vessey RS, et al. C-reactive protein in patients with COPD, control smokers and non-smokers. Thorax [Internet]. 2006 Jan [cited 2013 Jan 22];61(1):23–8. Available from: http://www.pubmedcentral.nih.gov/articlerender.fcgi?artid=2080714&tool=pmcentrez&rendertype=abstract

102. Confalonieri M, Mainardi E, Della Porta R, Bernorio S, Gandola L, Beghè B, et al. Inhaled corticosteroids reduce neutrophilic bronchial inflammation in patients with chronic obstructive pulmonary disease. Thorax [Internet]. 1998 Jul;53(7):583–5. Available from: http://www.pubmedcentral.nih.gov/articlerender.fcgi?artid=1745263&tool=pmcentrez&rendertype=abstract

103. Delaunay F, Khan A, Cintra A, Davani B, Ling Z-C, Andersson A, et al. Pancreatic beta cells are important targets for the diabetogenic effects of glucocorticoids. J Clin Invest [Internet]. 1997 Oct 15;100(8):2094–8. Available from: http://www.pubmedcentral.nih.gov/articlerender.fcgi?artid=508401&tool=pmcentrez&rendertype=abstract

104. Lambillotte C, Gilon P, Henquin J-C. Direct glucocorticoid inhibition of insulin secretion. An in vitro study of dexamethasone effects in mouse islets. J Clin Invest [Internet]. 1997 Feb 1;99(3):414–23. Available from: http://www.pubmedcentral.nih.gov/articlerender.fcgi?artid=507814&tool=pmcentrez&rendertype=abstract

105. Drescher VS, Chen H, Romsos DR. Corticotropin-Releasing Hormone Decreases Feeding , Oxygen Consumption and Activity of Genetically Obese ( ob / ob ) and Lean Mice. J Nutr [Internet]. 1994;124(November 1993):524–30. Available from: http://jn.nutrition.org/content/124/4/524.long

106. Lutfy K, Aimiuwu O, Mangubat M, Shin C, Nerio N, Gomez R, et al. Nicotine stimulates secretion of corticosterone via both CRH and AVP receptors. J Neurochem [Internet]. 2012;120(6):1108–16. Available from: http://www.ncbi.nlm.nih.gov/pubmed/22191943

107. Andreis P, Neri G, Nussdorfer G. Corticotropin-releasing hormone (CRH) directly stimulates corticosterone secretion by the rat adrenal gland. Endocrinology [Internet]. 1991;128(2):1198–200. Available from: http://www.ncbi.nlm.nih.gov/pubmed/1846581

108. George SA, Khan S, Briggs H, Abelson JL. CRH-stimulated cortisol release and food intake in healthy, nonobese adults. Psychoneuroendocrinology [Internet]. 2010;35(4):607–12. Available from: http://www.ncbi.nlm.nih.gov/pmc/articles/PMC2843773/pdf/nihms148326.pdf

109. Slominski A, Zbytek B, Szczesniewski A, Semak I, Kaminski J, Sweatman T, et al. CRH stimulation of corticosteroids production in melanocytes is mediated by ACTH. Am J Physiol Endocrinol Metab [Internet]. 2005 Apr [cited 2012 Nov 26];288(4):E701-6. Available from: http://www.ncbi.nlm.nih.gov/pubmed/15572653

110. Sirianni R, Rehman KS, Carr BR, Parker CR, Rainey WE. Corticotropin-releasing hormone directly stimulates cortisol and the cortisol biosynthetic pathway in human fetal adrenal cells. J Clin Endocrinol Metab [Internet]. 2005 Jan [cited 2012 Dec 4];90(1):279–85. Available from: http://www.ncbi.nlm.nih.gov/pubmed/15494460

111. Jacobson L. Lower weight loss and food intake in protein-deprived, corticotropin releasing hormone-deficient mice correlate with glucocorticoid insufficiency. Endocrinology [Internet]. 1999 Aug;140(8):3543–51. Available from: http://www.ncbi.nlm.nih.gov/pubmed/10433210

112. Gammie SC, Negron A, Newman SM, Rhodes JS. Corticotropin-releasing factor inhibits maternal aggression in mice. Behav Neurosci [Internet]. 2004 Aug [cited 2013 Feb 27];118(4):805–14. Available from: http://www.ncbi.nlm.nih.gov/pubmed/15301606

113. Klampfl S, Neumann I, Bosch O. Reduced brain corticotropin-releasing factor receptor activation is required for adequate maternal care and maternal aggression in lactating rats. Eur J Neoroscince [Internet]. 2013; Available from: http://www.ncbi.nlm.nih.gov/pubmed/23742269

114. Young EA, Lewis J, Akil H. The preferential release of beta-endorphin from the anterior pituitary lobe by Corticotropin Releasing Factor (CRF). Peptides [Internet]. 1986;7(4):603–7. Available from: http://www.sciencedirect.com/science/article/pii/0196978186900343

115. Făgăraşan MO, Eskay R, Axelrod J. Interleukin 1 potentiates the secretion of beta-endorphin induced by secretagogues in a mouse pituitary cell line (AtT-20). Proc Natl Acad Sci U S A [Internet]. 1989 Mar;86(6):2070–3. Available from: http://www.pubmedcentral.nih.gov/articlerender.fcgi?artid=286849&tool=pmcentrez&rendertype=abstract

116. Făgărăşan MO, Bishop JF, Rinaudo MS, Axelrod J. Interleukin 1 induces early protein phosphorylation and requires only a short exposure for late induced secretion of beta-endorphin in a mouse pituitary cell line. Proc Natl Acad Sci U S A [Internet]. 1990 Apr;87(7):2555–9. Available from: http://www.pubmedcentral.nih.gov/articlerender.fcgi?artid=53728&tool=pmcentrez&rendertype=abstract

117. Sapolsky R, Rivier C, Yamamoto G, Plotsky P, Vale W. Interleukin-1 stimulates the secretion of hypothalamic corticotropin-releasing factor. Science (80- ) [Internet]. 1987;238(4826):522–4. Available from: http://www.ncbi.nlm.nih.gov/pubmed/2821621

118. Berkenbosch F, van Oers J, del Rey A, Tilders F, Besedovsky H. Corticotropin-releasing factor-producing neurons in the rat activated by interleukin-1. Science (80- ) [Internet]. 1987;238(4826):524–6. Available from: http://www.ncbi.nlm.nih.gov/pubmed/2443979

119. Tsagarakis S, Gillies G, Rees L, Besser M, Grossman A. Interleukin-1 directly stimulates the release of corticotrophin releasing factor from rat hypothalamus. Neuroendocrinology [Internet]. 1989;49(1):98–101. Available from: http://www.ncbi.nlm.nih.gov/pubmed/2785660

120. Grunfeld C, Zhao C, Fuller J, Pollock A, Moser A, Friedman J, et al. Endotoxin and Cytokines Induce Expression of Leptin, the ob Gene Product, in Hamsters: A Role for Leptin in the Anorexia of Infection. J Clin Invest [Internet]. 1996;997(9):2152–7. Available from: http://www.ncbi.nlm.nih.gov/pmc/articles/PMC507291/pdf/972152.pdf

121. Sarraf P, Frederich RC, Turner EM, Ma G, Jaskowiak NT, Rivet III DJ, et al. Multiple cytokines and acute inflammation raise mouse leptin levels: Potential role in inflammatory anorexia. J Exp Med [Internet]. 1997 Jan 6;185(1):171–5. Available from: http://www.pubmedcentral.nih.gov/articlerender.fcgi?artid=2196098&tool=pmcentrez&rendertype=abstract

122. Landgraf R, Neumann I, Holsboer F, Pittman Q. Interleukin-1 beta stimulates both central and peripheral release of vasopressin and oxytocin in the rat. Eur J Neurosci [Internet]. 1995;7(4):592–8. Available from: http://www.ncbi.nlm.nih.gov/pubmed/7620610

123. Daniel AL, Houlihan JL, Janice S B, Walsh JP. Type B Insulin Resistance Developing during Interferon alpha Therapy. Endocr Pract [Internet]. 2010;15(2):153–7. Available from: http://www.ncbi.nlm.nih.gov/pubmed/19289328

124. Gerrits AJ, Gitz E, Koekman C a, Visseren FL, van Haeften TW, Akkerman JWN. Induction of insulin resistance by the adipokines resistin , leptin , plasminogen activator inhibitor-1 and retinol binding protein 4 in human megakaryocytes. Haematologica [Internet]. 2012 Aug [cited 2013 Feb 9];97(8):1149–57. Available from: http://www.pubmedcentral.nih.gov/articlerender.fcgi?artid=3409811&tool=pmcentrez&rendertype=abstract

125. Moreno JA, Izquierdo MC, Sanchez-Niño MD, Suárez-Alvarez B, Lopez-Larrea C, Jakubowski A, et al. The inflammatory cytokines TWEAK and TNFα reduce renal klotho expression through NFκB. J Am Soc Nephrol [Internet]. 2011 Jul [cited 2013 Mar 2];22(7):1315–25. Available from: http://www.pubmedcentral.nih.gov/articlerender.fcgi?artid=3137579&tool=pmcentrez&rendertype=abstract

126. Yoshizumi M, Kurihara H, Morita T, Yamashita T, Oh-hashi Y, Sugiyama T, et al. Interleukin 1 increases the production of endothelin-1 by cultured endothelial cells. Biochem Biophys Res Commun [Internet]. 1990;166(1):324–9. Available from: http://www.ncbi.nlm.nih.gov/pubmed/2405848

127. Emery M, Muller MN, Wrangham RW, Lwanga JS, Potts KB. Urinary C-peptide tracks seasonal and individual variation in energy balance in wild chimpanzees. Horm Behav [Internet]. Elsevier Inc.; 2009;55(2):299–305. Available from: http://dx.doi.org/10.1016/j.yhbeh.2008.11.005

128. Thoa N, Eichelman B, Richardson J, Jacobowitz D. 6-Hydroxydopa depletion of brain norepinephrine and the function of aggressive behavior. Science (80- ) [Internet]. 1972;178(4056):75–7. Available from: http://www.ncbi.nlm.nih.gov/pubmed/4672239

129. Ossowska G, Klenk-Majewska B, Zebrowska-Lupina I. Acute effect of dopamine agonists and some antidepressants in stress-induced deficit of fighting behavior. Pol J Pharmacol [Internet]. 1996;48(4):403–8. Available from: http://www.ncbi.nlm.nih.gov/pubmed/9112680/

130. Bliziotes M, McLoughlin S, Gunness M, Fumagalli F, Jones SR, Caron MG. Bone histomorphometric and biomechanical abnormalities in mice homozygous for deletion of the dopamine transporter gene. Bone [Internet]. 2000 Jan;26(1):15–9. Available from: http://www.ncbi.nlm.nih.gov/pubmed/10617152

131. Sartorio A, Conti A, Ambrosi B, Muratori M, Morabito F, Faglia G. Osteocalcin levels in patients with microprolactinoma before and during medical treatment. J Endocrinol Invest [Internet]. 1990;13(5):419–22. Available from: http://www.ncbi.nlm.nih.gov/pubmed/1974270

132. de Leeuw van Weenen JE, Parlevliet ET, Schröder-van der Elst JP, van den Berg SA, Willems van Dijk K, Romijn JA, et al. Pharmacological modulation of dopamine receptor D2-mediated transmission alters the metabolic phenotype of diet induced obese and diet resistant C57Bl6 mice. Exp Diabetes Res [Internet]. 2011 Jan [cited 2013 Jan 23];2011:928523. Available from: http://www.pubmedcentral.nih.gov/articlerender.fcgi?artid=3096057&tool=pmcentrez&rendertype=abstract

133. Schwarz PB, Peever JH. Dopamine triggers skeletal muscle tone by activating D1-like receptors on somatic motoneurons. J Neurophysiol [Internet]. 2011 Sep [cited 2013 Feb 5];106(3):1299–309. Available from: http://www.ncbi.nlm.nih.gov/pubmed/21653722

134. Rubí B, Ljubicic S, Pournourmohammadi S, Carobbio S, Armanet M, Bartley C, et al. Dopamine D2-like receptors are expressed in pancreatic beta cells and mediate inhibition of insulin secretion. J Biol Chem [Internet]. 2005 Nov 4 [cited 2012 Oct 28];280(44):36824–32. Available from: http://www.ncbi.nlm.nih.gov/pubmed/16129680

135. Cincotta A, Tozzo E, Scislowski P. Bromocriptine/SKF38393 treatment ameliorates obesity and associated metabolic dysfunctions in obese (ob/ob) mice. Life Sci [Internet]. 1997;61(10):951–6. Available from: http://www.ncbi.nlm.nih.gov/pubmed/9296333

136. Borcherding DC, Hugo ER, Idelman G, De Silva A, Richtand NW, Loftus J, et al. Dopamine receptors in human adipocytes: expression and functions. PLoS One [Internet]. 2011 Jan [cited 2013 Jan 23];6(9):e25537. Available from: http://www.pubmedcentral.nih.gov/articlerender.fcgi?artid=3180449&tool=pmcentrez&rendertype=abstract

137. Davis LM, Michaelides M, Cheskin LJ, Moran TH, Aja S, Watkins PA, et al. Bromocriptine Administration Reduces Hyperphagia and Adiposity and Differentially Affects Dopamine D2 Receptor and Transporter Binding in Leptin-Receptor-Deficient Zucker Rats and Rats with Diet-Induced Obesity. Neuroendocrinology [Internet]. 2009;89(2):152–62. Available from: http://www.ncbi.nlm.nih.gov/pmc/articles/PMC2681080/pdf/nihms101851.pdf

138. Andres C, Meyer S, Dina OA, Levine JD, Hucho T. Quantitative automated microscopy ( QuAM ) elucidates growth factor specific signalling in pain sensitization. Mol Pain [Internet]. BioMed Central Ltd; 2010;6(1):98. Available from: http://www.molecularpain.com/content/6/1/98

139. Maeda H, Gopalrao KR, Suzuki R, Sasaguri S. Epidermal growth factor and insulin inhibit cell death in pancreatic beta cells by activation of PI3-kinase/AKT signaling pathway under oxidative stress. Transplant Proc [Internet]. 2004;36(4):1163–5. Available from: http://www.sciencedirect.com/science/article/pii/S0041134504004269

140. Hakonen E, Ustinov J, Mathijs I, Palgi J, Bouwens L, Miettinen PJ, et al. Epidermal growth factor (EGF)-receptor signalling is needed for murine beta cell mass expansion in response to high-fat diet and pregnancy but not after pancreatic duct ligation. Diabetologia [Internet]. 2011 Jul [cited 2013 Jan 24];54(7):1735–43. Available from: http://www.ncbi.nlm.nih.gov/pubmed/21509441

141. Tirassa P, Triaca V, Amendola T, Fiore M, Aloe L. EGF and NGF injected into the brain of old mice enhance BDNF and ChAT in proliferating subventricular zone. J Neurosci Res [Internet]. 2003;72(5):557–64. Available from: http://www.ncbi.nlm.nih.gov/pubmed/12749020

142. Gogg S, Smith U. Epidermal growth factor and transforming growth factor alpha mimic the effects of insulin in human fat cells and augment downstream signaling in insulin resistance. J Biol Chem [Internet]. 2002 Sep 27 [cited 2013 Feb 27];277(39):36045–51. Available from: http://www.ncbi.nlm.nih.gov/pubmed/12138086

143. Tsutsumi O, Taketani Y, Oka T. Evidence for the involvement of epidermal growth factor in fertility decline in aging female mice. Horm Res [Internet]. 1993;39:32–6. Available from: http://www.ncbi.nlm.nih.gov/pubmed/8365708

144. Tsutsumi O, Taketani Y, Oka. The uterine growth-promoting action of epidermal growth factor and its function in the fertility of mice. J Endocrinol [Internet]. 1993;138(3):437–44. Available from: http://www.ncbi.nlm.nih.gov/pubmed/8277218

145. Noguchi S, Ohba Y, Oka T. Involvement of epidermal growth factor deficiency in pathogenesis of oligozoospermia in streptozotocin-induced diabetic mice. Endocrinology [Internet]. 1990;127(5):2136–40. Available from: http://www.ncbi.nlm.nih.gov/pubmed/2146109

146. Okamoto S, Oka T. Evidence for physiological function of epidermal growth factor: pregestational sialoadenectomy of mice decreases milk production and increases offspring mortality during lactation period. Proc Natl Acad Sci U S A [Internet]. 1984 Oct;81(19):6059–63. Available from: http://www.pubmedcentral.nih.gov/articlerender.fcgi?artid=391858&tool=pmcentrez&rendertype=abstract

147. Zhang H, Torregrossa MM, Jutkiewicz EM, Shi Y, Rice KC, Woods JH, et al. Endogenous opioids upregulate brain-derived neurotrophic factor mRNA through δ- and μ-opioid receptors independent of antidepressant-like effects. Eur J Neurosci [Internet]. 2006;23(4):984–94. Available from: http://www.ncbi.nlm.nih.gov/pmc/articles/PMC1462954/pdf/nihms-9647.pdf

148. Su C, Chang Y, Pai H, Liu I, Lo C, Cheng J. Infusion of beta-endorphin improves insulin resistance in fructose-fed rats. Horm Metab Res [Internet]. 2004;36(8):571–7. Available from: http://www.ncbi.nlm.nih.gov/pubmed/15326568

149. Becker A, Schröder H, Brosz M, Grecksch G, Schneider-Stock R. Differences Between Two Substrains of AB Mice in the Opioid System. Pharmacol Biochem Behav [Internet]. 1997;58(3):763–6. Available from: http://www.ncbi.nlm.nih.gov/pubmed/9329070

150. McKay L, Kenney N, Edens N, Williams R, Woods S. Intracerebroventricular beta-endorphin increases food intake of rats. Life Sci [Internet]. 1981;29(14):1429–34. Available from: http://www.sciencedirect.com/science/article/pii/0024320581900060

151. McLaughlin CL, Baile CA. Autoimmunization against β-endorphin increases food intakes and body weights of obese rats. Physiol Behav [Internet]. 1985;35(3):365–70. Available from: http://www.sciencedirect.com/science/article/pii/0031938485903105

152. Zhao H, Thanthan S, Yannaing S, Kuwayama H. Involvement of endothelin B receptors in the endothelin-3-induced increase of ghrelin and growth hormone in Holstein steers. Peptides [Internet]. Elsevier Inc.; 2010 May [cited 2013 Jan 24];31(5):938–43. Available from: http://www.ncbi.nlm.nih.gov/pubmed/20156503

153. ThanThan S, Mekaru C, Seki N, Hidaka K, A Ueno, ThidarMyint H, et al. Endogenous ghrelin released in response to endothelin stimulates growth hormone secretion in cattle. Domest Anim Endocrinol [Internet]. 2010 Jan [cited 2013 Jan 24];38(1):1–12. Available from: http://www.ncbi.nlm.nih.gov/pubmed/19733462

154. Wilkes JJ, Hevener A, Olefsky J. Chronic endothelin-1 treatment leads to insulin resistance in vivo. Diabetes [Internet]. 2003;52(8):1904–9. Available from: http://diabetes.diabetesjournals.org/content/52/8/1904.full.pdf

155. Juan C-C, Fang VS, Huang Y-J, Kwok C-F, Hsu Y-P, Ho L-T. Endothelin-1 induces insulin resistance in conscious rats. Biochem Biophys Res Commun [Internet]. 1996 Oct 23;227(3):694–9. Available from: http://www.ncbi.nlm.nih.gov/pubmed/8885996

156. Ottosson-Seeberger A, Lundberg J, Alvestrand A, Ahlborg G. Exogenous endothelin-1 causes peripheral insulin resistance in healthy humans. Acta Physiol Scand [Internet]. 1997;161(2):211–20. Available from: http://www.ncbi.nlm.nih.gov/pubmed/9366964

157. Gregersen S, Thomsen J, Brock B, Hermansen K. Endothelin-1 stimulates insulin secretion by direct action on the islets of Langerhans in mice. Diabetologia [Internet]. 1996;39(9):1030–5. Available from: http://www.ncbi.nlm.nih.gov/pubmed/8877285

158. Xiong Y, Tanaka H, Richardson JA, Williams SC, Slaughter CA, Nakamura M, et al. Endothelin-1 stimulates leptin production in adipocytes. J Biol Chem [Internet]. 2001 Jul 27 [cited 2013 Mar 2];276(30):28471–7. Available from: http://www.ncbi.nlm.nih.gov/pubmed/11359784

159. Davenport A, Nunez D, Hall J, Kaumann A, Brown M. Autoradiographical localization of binding sites for porcine [125I]endothelin-1 in humans, pigs, and rats: functional relevance in humans. J Cardiovasc Pharmacol [Internet]. 1989;13:S166-70. Available from: http://www.ncbi.nlm.nih.gov/pubmed/2473298

160. Franco-Cereceda A. Endothelin- and neuropeptide Y-induced vasoconstriction of human epicardial coronary arteries in vitro. Br J Pharmacol [Internet]. 1989 Jul;97(3):968–72. Available from: http://www.pubmedcentral.nih.gov/articlerender.fcgi?artid=1854593&tool=pmcentrez&rendertype=abstract

161. Yanagisawa M, Kurihara H, Kimura S, Tomobe Y, Kobayashi M, Mitsui Y, et al. A novel potent vasoconstrictor peptide produced by vascular endothelial cells. Nature [Internet]. 1988;332(6163):411–5. Available from: http://www.nature.com/nature/journal/v332/n6163/abs/332411a0.html

162. Beatrice N, Tiziana A, Diego G, Nicoletta F, Enrico C, Domenico R. Epo is involved in angiogenesis in human glioma. J Neurooncol [Internet]. 2011;102(1):51–8. Available from: http://www.ingentaconnect.com/content/klu/neon/2011/00000102/00000001/00000294

163. Zwezdaryk KJ, Coffelt SB, Figueroa YG, Liu J, Phinney DG, LaMarca HL, et al. Erythropoietin, a hypoxia-regulated factor, elicits a pro-angiogenic program in human mesenchymal stem cells. Exp Hematol [Internet]. 2007 Apr [cited 2013 Jan 25];35(4):640–52. Available from: http://www.ncbi.nlm.nih.gov/pubmed/17379074

164. Kurachi H, Oka T. Changes in epidermal growth factor concentrations of submandibular gland, plasma and urine of normal and sialoadenectomized female mice during various reproductive stages. J Endocrinol [Internet]. 1985;106(2):197–202. Available from: http://www.ncbi.nlm.nih.gov/pubmed/3874924

165. Neumann I, Russell JA, Landgraf R. Oxytocin and vasopressin release within the supraoptic and paraventricular nuclei of pregnant, parturient and lactating rats: A microdialysis study. Neuroscience [Internet]. 1993;53(1):65–75. Available from: http://www.sciencedirect.com/science/article/pii/030645229390285N?np=y

166. Barkley MS, Geschwind II, Bradford GE. The Gestational and Progesterone Pattern Secretion of Estradiol , in Selected Testosterone Strains of Mice. Biol Reprod [Internet]. 1979;20:733–8. Available from: http://www.biolreprod.org/content/20/4/733.full.pdf

167. Karczewska-Kupczewska M, Kowalska I, Nikołajuk A, Adamska A, Zielińska M, Kamińska N, et al. Circulating brain-derived neurotrophic factor concentration is downregulated by intralipid/heparin infusion or high-fat meal in young healthy male subjects. Diabetes Care [Internet]. 2012 Feb [cited 2013 Jan 18];35(2):358–62. Available from: http://www.ncbi.nlm.nih.gov/pubmed/22210566

168. Boden G, Chen X, Capulong E, Mozzoli M. Effects of free fatty acids on gluconeogenesis and autoregulation of glucose production in type 2 diabetes. Diabetes [Internet]. 2001 Apr;50(4):810–6. Available from: http://www.ncbi.nlm.nih.gov/pubmed/11289046

169. Bajaj M, Medina-Navarro R, Suraamornkul S, Meyer C, DeFronzo RA, Mandarino LJ. Paradoxical changes in muscle gene expression in insulin-resistant subjects after sustained reduction in plasma free fatty acid concentration. Diabetes [Internet]. 2007 Mar [cited 2013 Feb 25];56(3):743–52. Available from: http://www.ncbi.nlm.nih.gov/pubmed/17327445

170. Dresner A, Laurent D, Marcucci M, Griffin ME, Dufour S, Cline GW, et al. Effects of free fatty acids on glucose transport and IRS-1-associated phosphatidylinositol 3-kinase activity. J Clin Invest [Internet]. 1999 Jan;103(2):253–9. Available from: http://www.pubmedcentral.nih.gov/articlerender.fcgi?artid=407880&tool=pmcentrez&rendertype=abstract

171. Roden M, Price TB, Perseghin G, Petersen KF, Rothman DL, Cline GW, et al. Mechanism of Free Fatty Acid – induced Insulin Resistance in Humans. J Clin Invest [Internet]. 1996;97(12):2859–65. Available from: http://www.ncbi.nlm.nih.gov/pmc/articles/PMC507380/pdf/972859.pdf

172. Chowdhury P, Rayford PL. Effect of food restriction on plasma cholecystokinin levels and exocrine pancreatic function in rats. Ann Clin Lab Sci [Internet]. 2001 Oct;31(4):376–82. Available from: http://www.ncbi.nlm.nih.gov/pubmed/11688849

173. Hodge SJ, Thornton A, Flower TP, Clutton-Brock TH. Food limitation increases aggression in juvenile meerkats. Behav Ecol [Internet]. 2009 Jul 17 [cited 2013 Mar 1];20(5):930–5. Available from: http://www.beheco.oxfordjournals.org/cgi/doi/10.1093/beheco/arp071

174. Strubbe J. Parasympathetic involvement in rapid meal-associated conditioned insulin secretion in the rat. Am J Physiol [Internet]. 1992;262(3.2):R615-8. Available from: http://www.ncbi.nlm.nih.gov/pubmed/1357982

175. Stork O, Ji F-Y, Kaneko K, Stork S, Yoshinobu Y, Moriya T, et al. Postnatal development of a GABA deficit and disturbance of neural functions in mice lacking GAD65. Brain Res [Internet]. 2000;865(1):45–58. Available from: http://www.ncbi.nlm.nih.gov/pubmed/10814732

176. Kaufman KR, Kugler SL, Sachdeo RC. Tiagabine in the Management of Postencephalitic Epilepsy and Impulse Control Disorder. Epilepsy Behav [Internet]. 2002;3(2):190–4. Available from: http://www.ncbi.nlm.nih.gov/pubmed/12609422

177. Liu G-X, Liu S, Cai G-Q, Sheng Z-J, Cai Y-Q, Jiang J, et al. Reduced aggression in mice lacking GABA transporter subtype 1. J Neurosci Res [Internet]. 2007;85(3):649–55. Available from: http://www.ncbi.nlm.nih.gov/pubmed/17183588

178. Bhattarai JP, Park SA, Park JB, Lee SY, Herbison AE, Ryu PD, et al. Tonic extrasynaptic GABA(A) receptor currents control gonadotropin-releasing hormone neuron excitability in the mouse. Endocrinology [Internet]. 2011 Apr [cited 2013 Feb 28];152(4):1551–61. Available from: http://www.ncbi.nlm.nih.gov/pubmed/21285326

179. Coscina D, Nobrega J. Anorectic potency of inhibiting GABA transaminase in brain: studies of hypothalamic, dietary and genetic obesities. Int J Obes [Internet]. 1984;8:191–200. Available from: http://www.ncbi.nlm.nih.gov/pubmed/6534893

180. Coscina D V., Lloyd KG. Medial hypothalamic obesity: Association with impaired hypothalamic GABA synthesis. Brain Res Bull [Internet]. 1980;5(2):793–6. Available from: http://www.sciencedirect.com/science/article/pii/036192308090132X

181. Pu S, Jain MR, Horvath TL, Diano S, Kalra PS, Kalra SP. Interactions between Neuropeptide Y and Gamma-Aminobutyric Acid in Stimulation of Feeding : A Morphological and Pharmacological Analysis. Endocrinology [Internet]. 1999;140(2):933–40. Available from: http://www.ncbi.nlm.nih.gov/pubmed/9927326

182. Bannai M, Ichikawa M, Nishihara M, Takahashi M. Effect of injection of antisense oligodeoxynucleotides of GAD isozymes into rat ventromedial hypothalamus on food intake and locomotor activity. Brain Res [Internet]. 1998;784(1–2):305–15. Available from: http://www.ncbi.nlm.nih.gov/pubmed/9518663

183. Tian J, Lu Y, Zhang H, Chau CH, Dang HN, Kaufman DL. Gamma-aminobutyric acid inhibits T cell autoimmunity and the development of inflammatory responses in a mouse type 1 diabetes model. J Immunol [Internet]. 2004 Oct 15;173(8):5298–304. Available from: http://www.ncbi.nlm.nih.gov/pubmed/15470076

184. Purwana I, Zheng J, Li X, Deurloo M, Son DO, Zhang Z, et al. GABA promotes human ??-cell proliferation and modulates glucose homeostasis. Diabetes [Internet]. 2014;63(12):4197–205. Available from: http://diabetes.diabetesjournals.org/content/63/12/4197.long

185. Rooman I, Lardon J, Bouwens L. Gastrin stimulates beta-cell neogenesis and increases islet mass from transdifferentiated but not from normal exocrine pancreas tissue. Diabetes [Internet]. 2002;51:686–90. Available from: http://www.ncbi.nlm.nih.gov/pubmed/11872667

186. Li Q, Deng X, Singh P. Significant increase in the aggressive behavior of transgenic mice overexpressing peripheral progastrin peptides: associated changes in CCK2 and serotonin receptors in the CNS. Neuropsychopharmacology [Internet]. 2007 Aug [cited 2013 Feb 16];32(8):1813–21. Available from: http://www.ncbi.nlm.nih.gov/pubmed/17228339

187. Cowey SL, Quast M, Belalcazar LM, Wei J, Deng X, Given R, et al. Abdominal obesity, insulin resistance, and colon carcinogenesis are increased in mutant mice lacking gastrin gene expression. Cancer [Internet]. 2005 Jun 15 [cited 2013 Feb 16];103(12):2643–53. Available from: http://www.ncbi.nlm.nih.gov/pubmed/15864814

188. Chen P, Gao Y, Geng L, Ping V, Gao Y, Geng L, et al. Plasma butyrylcholinesterase regulates ghrelin to control aggression. Proc Natl Acad Sci U S A [Internet]. 2015;112(12):2251–6. Available from: http://www.pnas.org/content/112/7/2251.full.pdf?with-ds=yes

189. Asakawa A, Inui A, Kaga T, Katsuura G, Fujimiya M, Fujino MA, et al. Antagonism of ghrelin receptor reduces food intake and body weight gain in mice. Gut [Internet]. 2003 Jul;52(7):947–52. Available from: http://www.pubmedcentral.nih.gov/articlerender.fcgi?artid=1773718&tool=pmcentrez&rendertype=abstract

190. Nakazato M, Murakami N, Date Y, Kojima M, Matsuo H, Kangawa K, et al. A role for ghrelin in the central regulation of feeding. Nature [Internet]. 2001 Jan 11;409(6817):194–8. Available from: http://www.ncbi.nlm.nih.gov/pubmed/11196643

191. Wren A, Seal L, Cohen M, Brynes A, Frost G, Murphy K, et al. Ghrelin enhances appetite and increases food intake in humans. J Clin Endocrinol Metab [Internet]. 2001;86(12):5992–5. Available from: http://jcem.endojournals.org/content/86/12/5992.full.pdf

192. Wren A, Small C, Ward H, Murphy K, Dakin C, Taheri S, et al. The novel hypothalamic peptide ghrelin stimulates food intake and growth hormone secretion. Endocrinology [Internet]. 2000;141(11):4325–8. Available from: http://endo.endojournals.org/content/141/11/4325.full.pdf

193. Tschöp M, Smiley DL, Heiman ML. Ghrelin induces adiposity in rodents. Nature [Internet]. 2000 Oct 19;407(6806):908–13. Available from: http://www.ncbi.nlm.nih.gov/pubmed/11057670

194. Naleid AM, Grace MK, Cummings DE, Levine AS. Ghrelin induces feeding in the mesolimbic reward pathway between the ventral tegmental area and the nucleus accumbens. Peptides [Internet]. 2005 Nov [cited 2013 Nov 14];26(11):2274–9. Available from: http://www.ncbi.nlm.nih.gov/pubmed/16137788

195. Abizaid A, Liu Z, Andrews ZB, Shanabrough M, Borok E, Elsworth JD, et al. Ghrelin modulates the activity and synaptic input organization of midbrain dopamine neurons while promoting appetite. J Clin Invest [Internet]. 2006;116(12):3229–39. Available from: http://www.ncbi.nlm.nih.gov/pmc/articles/PMC1618869/pdf/JCI0629867.pdf

196. Date Y, Murakami N, Kojima M, Kuroiwa T, Matsukura S, Kangawa K, et al. Central effects of a novel acylated peptide, ghrelin, on growth hormone release in rats. Biochem Biophys Res Commun [Internet]. 2000 Aug 28 [cited 2012 Nov 11];275(2):477–80. Available from: http://www.ncbi.nlm.nih.gov/pubmed/10964690

197. Kojima M, Hosoda H, Date Y, Nakazato M, Matsuo H, Kangawa K. Ghrelin is a growth-hormone-releasing acylated peptide from stomach. Nature [Internet]. 1999 Dec 9;402(6762):656–60. Available from: http://www.ncbi.nlm.nih.gov/pubmed/15533773

198. Arvat E, Maccario M, Di Vito L, Broglio F, Benso A, Gottero C, et al. Endocrine activities of ghrelin, a natural growth hormone secretagogue (GHS), in humans: comparison and interactions with hexarelin, a nonnatural peptidyl GHS, and GH-releasing hormone. J Clin Endocrinol Metab [Internet]. 2001 Mar;86(3):1169–74. Available from: http://www.ncbi.nlm.nih.gov/pubmed/11238504

199. Feng DD, Yang S-K, Loudes C, Simon A, Al-Sarraf T, Culler M, et al. Ghrelin and obestatin modulate growth hormone-releasing hormone release and synaptic inputs onto growth hormone-releasing hormone neurons. Eur J Neurosci [Internet]. 2011 Sep [cited 2013 Feb 13];34(5):732–44. Available from: http://www.ncbi.nlm.nih.gov/pubmed/21777303

200. Arvat E, Di Vito L, Broglio F, Papotti M, Muccioli G, Dieguez C, et al. Preliminary evidence that Ghrelin, the natural GH secretagogue (GHS)-receptor ligand, strongly stimulates GH secretion in humans. J Endocrinol Invest [Internet]. 2000;23(8):493–5. Available from: http://www.ncbi.nlm.nih.gov/pubmed/11021763

201. Lee H-M, Wang G, Englander EW, Kojima M, Greeley GH. Ghrelin, a new gastrointestinal endocrine peptide that stimulates insulin secretion: enteric distribution, ontogeny, influence of endocrine, and dietary manipulations. Endocrinology [Internet]. 2002 Jan;143(1):185–90. Available from: http://www.ncbi.nlm.nih.gov/pubmed/11751608

202. Cruz MT, Herman MA, Cote DM, Ryabinin AE, Roberto M. Ghrelin Increases GABAergic Transmission and Interacts with Ethanol Actions in the Rat Central Nucleus of the Amygdala. Neuropsychopharmacology [Internet]. Nature Publishing Group; 2012;38(2):364–75. Available from: http://dx.doi.org/10.1038/npp.2012.190

203. Cowley MA, Smith RG, Diano S, Tschöp M, Pronchuk N, Grove KL, et al. The distribution and mechanism of action of ghrelin in the CNS demonstrates a novel hypothalamic circuit regulating energy homeostasis. Neuron [Internet]. 2003 Feb 20;37(4):649–61. Available from: http://www.ncbi.nlm.nih.gov/pubmed/12597862

204. Wu R, Dong W, Zhou M, Cui X, Hank Simms H, Wang P. Ghrelin improves tissue perfusion in severe sepsis via downregulation of endothelin-1. Cardiovasc Res [Internet]. 2005 Nov 1 [cited 2013 Mar 2];68(2):318–26. Available from: http://www.ncbi.nlm.nih.gov/pubmed/16018991

205. Ikezawa Y, Yamatani K, Ogawa A, Ohnuma H, Igarashi M, Daimon M, et al. Effects of glucagon on glycogenolysis and gluconeogenesis are region-specific in periportal and perivenous hepatocytes. J Lab Clin Med [Internet]. 1998;132(6):547–55. Available from: http://www.ncbi.nlm.nih.gov/pubmed/9851746

206. Beuers U, Jungermann K. Relative contribution of glycogenolysis and gluconeogenesis to basal, glucagon- and nerve stimulation-dependent glucose output in the perfused liver from fed and fasted rats. Biochem Int [Internet]. 1990;21(3):405–13. Available from: http://www.ncbi.nlm.nih.gov/pubmed/2222488

207. Donkin S, Armentano L. Regulation of gluconeogenesis by insulin and glucagon in the neonatal bovine. Am J Physiol [Internet]. 1994;266(4.2):R1229–37. Available from: http://www.ncbi.nlm.nih.gov/pubmed/8184966

208. Chari M, Yang CS, Lam CKL, Lee K, Mighiu P, Kokorovic A, et al. Glucose transporter-1 in the hypothalamic glial cells mediates glucose sensing to regulate glucose production in vivo. Diabetes [Internet]. 2011 Jul [cited 2013 Feb 4];60(7):1901–6. Available from: http://www.pubmedcentral.nih.gov/articlerender.fcgi?artid=3121426&tool=pmcentrez&rendertype=abstract

209. Millesi E, Hoffmann IE, Steurer S, Metwaly M, Dittami JP. Vernal changes in the behavioral and endocrine responses to GnRH application in male European ground squirrels. Horm Behav [Internet]. 2002 Feb [cited 2013 Mar 1];41(1):51–8. Available from: http://www.ncbi.nlm.nih.gov/pubmed/11863383

210. Chandrashekar V, Bartke A. The Role of Growth Hormone in the Control of Gonadotropin Secretion in Adult Male Rats. Endocrinology [Internet]. 1998;139(3):1067–74. Available from: http://www.ncbi.nlm.nih.gov/pubmed/9492039

211. Wallymahmed M, Foy P, Shaw D, Hutcheon R, Edwards R, MacFarlane I. Quality of life, body composition and muscle strength in adult growth hormone deficiency: the influence of growth hormone replacement therapy for up to 3 years. Clin Endocrinol (Oxf) [Internet]. 1997;47(4):439–46. Available from: http://www.ncbi.nlm.nih.gov/pubmed/9404442

212. Cuneo R, Salomon F, Wiles C, Hesp R, Sönksen P. Growth hormone treatment in growth hormone-deficient adults. I. Effects on muscle mass and strength. J Appl Physiol [Internet]. 1991;70(2):688–94. Available from: http://www.ncbi.nlm.nih.gov/pubmed/2022560

213. Johannsson G, Grimby G, Sunnerhagen KS, Bengtsson B-Å. Two years of growth hormone (GH) treatment increase isometric and isokinetic muscle strength in GH-deficient adults. J Clin Endocrinol Metab [Internet]. 1997 Sep;82(9):2877–84. Available from: http://www.ncbi.nlm.nih.gov/pubmed/9284713

214. Matte A. Growth hormone and isolation-induced aggression in wild male mice. Pharmacol Biochem Behav [Internet]. 1981;14:85–7. Available from: http://www.ncbi.nlm.nih.gov/pubmed/7195589

215. Krag MB, Gormsen LC, Guo Z, Christiansen JS, Jensen MD, Nielsen S, et al. Growth hormone-induced insulin resistance is associated with increased intramyocellular triglyceride content but unaltered VLDL-triglyceride kinetics. Am J Physiol Endocrinol Metab [Internet]. 2007 Mar [cited 2013 Feb 16];292(3):E920-7. Available from: http://www.ncbi.nlm.nih.gov/pubmed/17132823

216. Rizza R, Mandarino L, Gerich J. Effects of growth hormone on insulin action in man. Mechanisms of insulin resistance, impaired suppression of glucose production, and impaired stimulation of glucose utilization. Diabetes [Internet]. 1982;31(8.1):663–9. Available from: http://www.ncbi.nlm.nih.gov/pubmed/6761205

217. Takano A, Haruta T, Iwata M, Usui I, Uno T, Kawahara J, et al. Growth hormone induces cellular insulin resistance by Uncoupling Phosphatidylinositol 3-Kinase and Its Downstream Signals in 3T3-L1 Adipocytes. Diabetes [Internet]. 2001;50(8):1891–900. Available from: http://diabetes.diabetesjournals.org/content/50/8/1891.full.pdf

218. Bramnert M, Segerlantz M, Laurila E, Daugaard JR, Manhem P, Groop L. Growth Hormone Replacement Therapy Induces Insulin Resistance by Activating the Glucose-Fatty Acid Cycle. J Clin Endocrinol Metab [Internet]. 2003 Apr 1 [cited 2013 Feb 21];88(4):1455–63. Available from: http://jcem.endojournals.org/cgi/doi/10.1210/jc.2002-020542

219. Vestergaard ET, Krag MB, Poulsen MM, Pedersen SB, Moller N, Jorgensen JOL, et al. Ghrelin- and GH-induced insulin resistance: no association with retinol-binding protein-4. Endocr Connect [Internet]. 2013 Jun 1 [cited 2013 Jun 20];2(2):96–103. Available from: http://www.ncbi.nlm.nih.gov/pubmed/23781325

220. Gao Y, Su P, Wang C, Zhu K, Chen X, Liu S, et al. The Role of PTEN in Chronic Growth Hormone-Induced Hepatic Insulin Resistance. PLoS One [Internet]. 2013 Jan [cited 2013 Jul 12];8(6):e68105. Available from: http://www.ncbi.nlm.nih.gov/pubmed/23840818

221. Breier B, Vickers M, Gravance C, Casey P. Growth hormone (GH) therapy markedly increases the motility of spermatozoa and the concentration of insulin-like growth factor-I in seminal vesicle fluid in the male GH-deficient dwarf rat. Endocrinology [Internet]. 1996;137(9):4061–4. Available from: http://www.ncbi.nlm.nih.gov/pubmed/8756586

222. O’Connell T, Clemmons DR. IGF-I/IGF-Binding Protein-3 Combination Improves Insulin Resistance By GH-Dependent and Independent Mechanisms. J Clin Endocrinol Metab [Internet]. 2002 Sep 1 [cited 2013 Feb 7];87(9):4356–60. Available from: http://jcem.endojournals.org/cgi/doi/10.1210/jc.2002-020343

223. Roman-garcia P, Quiros-gonzalez I, Mottram L, Lieben L, Sharan K, Wangwiwatsin A, et al. Vitamin B 12 – dependent taurine synthesis regulates growth and bone mass. J Clin Invest [Internet]. 2014;124(7):2988–3002. Available from: http://www.jci.org/articles/view/72606/version/2/pdf/render

224. Masaki T, Chiba S, Yasuda T, Noguchi H, Kakuma T, Watanabe T, et al. Involvement of hypothalamic histamine H1 receptor in the regulation of feeding rhythm and obesity. Diabetes [Internet]. 2004 Sep;53(9):2250–60. Available from: http://www.ncbi.nlm.nih.gov/pubmed/15331534

225. Malmlöf K, Zaragoza F, Golozoubova V, Refsgaard HHF, Cremers T, Raun K, et al. Influence of a selective histamine H3 receptor antagonist on hypothalamic neural activity, food intake and body weight. Int J Obes [Internet]. 2005 Dec [cited 2013 Mar 2];29(12):1402–12. Available from: http://www.ncbi.nlm.nih.gov/pubmed/16151415

226. Yoshimatsu H, Chiba S, Tajima D, Akehi Y, Sakata T. Histidine suppresses food intake through its conversion into neuronal histamine. Exp Biol Med (Maywood) [Internet]. 2002 Jan;227(1):63–8. Available from: http://www.ncbi.nlm.nih.gov/pubmed/11788786

227. Masaki T, Chiba S, Yoshimichi G, Yasuda T, Noguchi H, Kakuma T, et al. Neuronal Histamine Regulates Food Intake, Adiposity, and Uncoupling Protein Expression in Agouti Yellow (Ay/a) Obese Mice. Endocrinology [Internet]. 2003 Jun 1 [cited 2013 Mar 2];144(6):2741–8. Available from: http://endo.endojournals.org/cgi/doi/10.1210/en.2003-0031

228. Yanai K, Son LZ, Endou M, Sakurai E, Nakagawasai O, Tadano T, et al. Behavioural characterization and amounts of brain monoamines and their metabolites in mice lacking histamine H1 receptors. Neuroscience [Internet]. 1998 Nov;87(2):479–87. Available from: http://www.ncbi.nlm.nih.gov/pubmed/9740406

229. Moses A, Young S, Morrow L, O’Brien M, Clemmons D. Recombinant human insulin-like growth factor I increases insulin sensitivity and improves glycemic control in type II diabetes. Diabetes [Internet]. 1996;45(1):91–100. Available from: http://www.ncbi.nlm.nih.gov/pubmed/8522066

230. Morrow L, O’Brien M, Moller D, Flier J, Moses A. Recombinant human insulin-like growth factor-I therapy improves glycemic control and insulin action in the type A syndrome of severe insulin resistance. J Clin Endocrinol Metab [Internet]. 1994;79(1):205–10. Available from: http://www.ncbi.nlm.nih.gov/pubmed/8027228

231. Laron Z, Avitzur Y, Klinger B. Carbohydrate metabolism in primary growth hormone resistance (Laron syndrome) before and during insulin-like growth factor-I treatment. Metabolism [Internet]. 1995;44(10):113–8. Available from: http://www.ncbi.nlm.nih.gov/pubmed/7476303

232. Frick F, Oscarsson J, Vikman-Adolfsson K, Ottosson M, Yoshida N, Eden S. Different effects of IGF-I on insulin-stimulated glucose uptake in adipose tissue and skeletal muscle. Am J Physiol Endocrinol Metab [Internet]. 2000;278:E729–37. Available from: http://ajpendo.physiology.org/content/278/4/E729.full.pdf

233. Vestergaard H, Rossen M, Urhammer S, Müller J, Pedersen O. Short- and long-term metabolic effects of recombinant human IGF-I treatment in patients with severe insulin resistance and diabetes mellitus. Eur J Endocrinol [Internet]. 1997;136(5):475–82. Available from: http://www.ncbi.nlm.nih.gov/pubmed/9186267

234. Yakar S, Liu J, Fernandez AM, Wu Y, Schally A V, Frystyk J, et al. Liver-specific igf-1 Gene Deletion Leads to Muscle Insulin Insensitivity. Diabetes [Internet]. 2001;50(May):1110–8. Available from: http://diabetes.diabetesjournals.org/content/50/5/1110.full.pdf

235. Kulkarni RN, Holzenberger M, Shih DQ, Ozcan U, Stoffel M, Magnuson MA, et al. beta-cell-specific deletion of the Igf1 receptor leads to hyperinsulinemia and glucose intolerance but does not alter beta-cell mass. Nat Genet [Internet]. 2002 May [cited 2014 Jun 16];31(1):111–5. Available from: http://www.ncbi.nlm.nih.gov/pubmed/11923875

236. Vickers MH, Ikenasio BA, Breier BH. IGF-I Treatment Reduces Hyperphagia, Obesity, and Hypertension in Metabolic Disorders Induced by Fetal Programming. Endocrinology [Internet]. 2001;142(9):3964–73. Available from: http://endo.endojournals.org/content/142/9/3964.full.pdf

237. George M, Ayuso E, Casellas A, Costa C, Devedjian JC, Bosch F. β cell expression of IGF-I leads to recovery from type 1 diabetes. J Clin Invest [Internet]. 2002;109(9):1153–63. Available from: http://www.ncbi.nlm.nih.gov/pmc/articles/PMC150958/pdf/JCI0212969.pdf

238. Lu H, Martinez-Nieves B, Lapanowski K, Dunbar J. Intracerebroventricular insulin-like growth factor-1 decreases feeding in diabetic rats. Endocrine [Internet]. 2001 Apr;14(3):349–52. Available from: http://www.ncbi.nlm.nih.gov/pubmed/11444432

239. Barton-Davis ER, Shoturma DI, Musaro A, Rosenthal N, Sweeney HL. Viral mediated expression of insulin-like growth factor I blocks the aging-related loss of skeletal muscle function. Proc Natl Acad Sci U S A [Internet]. 1998 Dec 22;95(26):15603–7. Available from: http://www.pubmedcentral.nih.gov/articlerender.fcgi?artid=28090&tool=pmcentrez&rendertype=abstract

240. Musarò A, McCullagh K, Paul A, Houghton L, Dobrowolny G, Molinaro M, et al. Localized Igf-1 transgene expression sustains hypertrophy and regeneration in senescent skeletal muscle. Nat Genet [Internet]. 2001;27:195–200. Available from: http://www.nature.com/ng/journal/v27/n2/pdf/ng0201_195.pdf

241. Shavlakadze T, White J, Davies M, Hoh J, Grounds M. Insulin-like growth factor I slows the rate of denervation induced skeletal muscle atrophy. Neuromuscul Disord [Internet]. 2005;15(2):139–46. Available from: http://www.ncbi.nlm.nih.gov/pubmed/15694135

242. Anderson BC, Christiansen SP, Grandt S, Grange RW, Mcloon LK. Increased Extraocular Muscle Strength with Direct Injection of Insulin-like Growth Factor-I. Invest Ophthalmol Vis Sci [Internet]. 2006;47(6):2461–7. Available from: http://www.ncbi.nlm.nih.gov/pmc/articles/PMC3039316/pdf/nihms269083.pdf

243. Van Schravendijk C, Heylen L, Van den Brande J, Pipeleers D. Direct effect of insulin and insulin-like growth factor-I on the secretory activity of rat pancreatic beta cells. Diabetologia [Internet]. 1990;33(11):649–53. Available from: http://www.ncbi.nlm.nih.gov/pubmed/2076797

244. Yamashita S, Melmed S. Insulinlike Growth Factor I Regulation of Growth Hormone Gene Transcription in Primary Rat Pituitary Cells. J Clin Invest [Internet]. 1987;79(February):449–52. Available from: http://www.ncbi.nlm.nih.gov/pmc/articles/PMC424098/pdf/jcinvest00113-0139.pdf

245. Min S, MacKenzie D, Breier B, McCutcheon S, Gluckman P. Responses of young energy-restricted sheep to chronically administered insulin-like growth factor I (IGF-I): evidence that IGF-I suppresses the hepatic growth hormone receptor. Endocrinology [Internet]. 1996;137(3):1129–37. Available from: http://www.ncbi.nlm.nih.gov/pubmed/8603584

246. Yakar S, Rosen CJ, Beamer WG, Ackert-bicknell CL, Wu Y, Liu J-L, et al. Circulating levels of IGF-1 directly regulate bone growth and density. J Clin Invest [Internet]. 2002;110(6):771–81. Available from: http://www.jci.org/articles/view/15463/pdf

247. Spies M, Nesic O, Barrow RE, Perez-Polo JR, Herndon DN. Liposomal IGF-1 gene transfer modulates pro- and anti-inflammatory cytokine mRNA expression in the burn wound. Gene Ther [Internet]. 2001 Sep;8(18):1409–15. Available from: http://www.ncbi.nlm.nih.gov/pubmed/11571581

248. Alleva E, Cirulli F, Bianchi M, Bondiolotti G Pietro, Chiarotti F, Acetis L De, et al. Behavioural characterization of interleukin-6 overexpressing or deficient mice during agonistic encounters. Eur J Neurosci [Internet]. 1998;10(12):3664–72. Available from: http://www.ncbi.nlm.nih.gov/pubmed/9875345

249. Rotter V, Nagaev I, Smith U. Interleukin-6 (IL-6) induces insulin resistance in 3T3-L1 adipocytes and is, like IL-8 and tumor necrosis factor-alpha, overexpressed in human fat cells from insulin-resistant subjects. J Biol Chem [Internet]. 2003 Nov 14 [cited 2013 Mar 1];278(46):45777–84. Available from: http://www.ncbi.nlm.nih.gov/pubmed/12952969

250. Senn JJ, Klover PJ, Nowak IA, Mooney RA. Interleukin-6 Induces Cellular Insulin Resistance in Hepatocytes. Diabetes [Internet]. 2002;51(12):3391–9. Available from: http://diabetes.diabetesjournals.org/content/51/12/3391.full.pdf

251. Wallenius V, Wallenius K, Ahrén B, Rudling M, Carlsten H, Dickson SL, et al. Interleukin-6-deficient mice develop mature-onset obesity. Nat Med [Internet]. 2002;8(1):75–9. Available from: http://www.ncbi.nlm.nih.gov/pubmed/11786910

252. Starkie R, Ostrowski SR, Jauffred S, Febbraio M, Pedersen BK. Exercise and IL-6 infusion inhibit endotoxin-induced TNF- α production in humans. FASEB J [Internet]. 2003;17(8):884–6. Available from: http://www.ncbi.nlm.nih.gov/pubmed/12626436

253. Kahles F, Meyer C, Möllmann J, Diebold S, Findeisen HM, Lebherz C, et al. GLP-1 Secretion Is Increased by Inflammatory Stimuli in an IL-6-Dependent Manner, Leading to Hyperinsulinemia and Blood Glucose Lowering. Diabetes [Internet]. 2014;63(10):3221–9. Available from: https://www.ncbi.nlm.nih.gov/pubmed/24947356

254. Chida D, Osaka T, Hashimoto O, Iwakura Y. Combined Interleukin-6 and Interleukin-1 Deficienc Causes Obesity in Young Mice. Diabetes [Internet]. 2006;55(April):971–7. Available from: https://www.ncbi.nlm.nih.gov/pubmed/16567518

255. Arkan MC, Hevener AL, Greten FR, Maeda S, Li Z-W, Long JM, et al. IKK-β links inflammation to obesity-induced insulin resistance. Nat Med [Internet]. 2005;11:191–8. Available from: http://www.nature.com/nm/journal/v11/n2/full/nm1185.html

256. Hayek A, Beattie G, Cirulli V, Lopez A, Ricordi C, Rubin J. Growth factor/matrix-induced proliferation of human adult beta-cells. Diabetes [Internet]. 1995;44(12):1458–60. Available from: http://www.ncbi.nlm.nih.gov/pubmed/7589854

257. Riley KG, Pasek RC, Maulis MF, Peek J, Brigstock DR, Herrera PL, et al. CTGF modulates adult β-cell maturity and proliferation to promote β-cell regeneration in mice. Diabetes [Internet]. 2014;1–43. Available from: http://diabetes.diabetesjournals.org/content/early/2014/11/05/db14-1195.abstract

258. Johnson LR, Overholt BF. Release of Histamine into Gastric Venous Blood Following Injury by Acetic or Salicylic Acid. Gastroenterology [Internet]. The Williams & Wilkins Co.; 1967;52(3):505–9. Available from: http://dx.doi.org/10.1016/S0016-5085(67)80177-X

259. Coskun T, Bina HA, Schneider M a, Dunbar JD, Hu CC, Chen Y, et al. Fibroblast growth factor 21 corrects obesity in mice. Endocrinology [Internet]. 2008 Dec [cited 2013 Feb 28];149(12):6018–27. Available from: http://www.ncbi.nlm.nih.gov/pubmed/18687777

260. Bigi S, Maestripieri D, Aloe L, Alleva E. NGF decreases isolation-induced aggressive behavior, while increasing adrenal volume, in adult male mice. Physiol Behav [Internet]. 1992;51(2):337–43. Available from: http://www.sciencedirect.com/science/article/pii/003193849290150Z

261. Circolo A, Pierce GF, Katz Y, Strunk RC. Antiinflammatory Effects of Polypeptide Growth Factors. J Biol Chem Chem [Internet]. 1990;265(9):5066–71. Available from: http://www.jbc.org/content/265/9/5066.long

262. Fukumura D, Gohongi T, Kadambi A, Izumi Y, Ang J, Yun C, et al. Predominant role of endothelial nitric oxide synthase in vascular endothelial growth factor-induced angiogenesis and vascular permeability. Proc Natl Acad Sci U S A [Internet]. 2001;98(5):2604–9. Available from: http://www.pnas.org/content/98/5/2604.full.pdf

263. Craft S, Newcomer L, Kanne S, Dagogo-Jack S, Cryer P, Sheline Y, et al. Memory Improvement Following Induced Hyperinsulinemia in Alzheimer â€^TM^ s Disease. Neurobiol Aging [Internet]. 1996;17(1):123–30. Available from: http://ac.els-cdn.com/0197458095020020/1-s2.0-0197458095020020-main.pdf?_tid=10c76306-63b3-11e2-bdd1-00000aab0f6b&acdnat=1358763332_1af746f9749bd1b7c6b93a1d26d1c746

264. Kern W, Peters A, Fruehwald-Schultes B, Deininger E, Born J, Fehm H. Improving influence of insulin on cognitive functions in humans. Neuroendocrinology [Internet]. 2011;74(4):270–80. Available from: http://www.ncbi.nlm.nih.gov/pubmed/11598383

265. Schmidt H, Kern W, Giese R, Hallschmid M, Enders A. Intranasal insulin to improve developmental delay in children with 22q13 deletion syndrome: an exploratory clinical trial. J Med Genet [Internet]. 2009;46(4):217–22. Available from: http://www.ncbi.nlm.nih.gov/pubmed/18948358

266. Benedict C, Hallschmid M, Schmitz K, Schultes B, Ratter F, Fehm HL, et al. Intranasal insulin improves memory in humans: superiority of insulin aspart. Neuropsychopharmacology [Internet]. 2007 Jan [cited 2013 Feb 26];32(1):239–43. Available from: http://www.ncbi.nlm.nih.gov/pubmed/16936707

267. Francis GJ, Martinez JA, Liu WQ, Xu K, Ayer A, Fine J, et al. Intranasal insulin prevents cognitive decline, cerebral atrophy and white matter changes in murine type I diabetic encephalopathy. Brain [Internet]. 2008 Dec [cited 2013 Apr 9];131(Pt 12):3311–34. Available from: http://www.ncbi.nlm.nih.gov/pubmed/19015157

268. Frick GP, Goodman HM. Insulin regulation of branched chain alpha-keto acid dehydrogenase in adipose tissue. J Biol Chem [Internet]. 1980 Jul 10;255(13):6186–92. Available from: http://www.pubmedcentral.nih.gov/articlerender.fcgi?artid=1138345&tool=pmcentrez&rendertype=abstract

269. Widjaja A, Stratton IM, Horn R, Holman RR, Turner R, Brabant G. Plasma leptin, obesity, and plasma insulin in type 2 diabetic subjects. J Clin Endocrinol Metab [Internet]. 1997 Feb;82(2):654–7. Available from: http://www.ncbi.nlm.nih.gov/pubmed/9024271

270. Chen C-D, Podvin S, Gillespie E, Leeman SE, Abraham CR. Insulin stimulates the cleavage and release of the extracellular domain of Klotho by ADAM10 and ADAM17. Proc Natl Acad Sci U S A [Internet]. 2007 Dec 11;104(50):19796–801. Available from: http://www.pubmedcentral.nih.gov/articlerender.fcgi?artid=2148378&tool=pmcentrez&rendertype=abstract

271. Kasayama S, Ohba Y, Oka T. Epidermal growth factor deficiency associated with diabetes mellitus. Proc Natl Acad Sci U S A [Internet]. 1989;86(19):7644–8. Available from: http://www.ncbi.nlm.nih.gov/pmc/articles/PMC298123/pdf/pnas00286-0354.pdf

272. Wolpert H, Steen S, Istfan N, Simonson D. Insulin modulates circulating endothelin-1 levels in humans. Metabolism [Internet]. 1993;42(8):1027–30. Available from: http://www.ncbi.nlm.nih.gov/pubmed/8345807

273. Kim HH, DiVall S a, Deneau RM, Wolfe A. Insulin regulation of GnRH gene expression through MAP kinase signaling pathways. Mol Cell Endocrinol [Internet]. 2005 Oct 20 [cited 2013 Mar 1];242(1–2):42–9. Available from: http://www.ncbi.nlm.nih.gov/pubmed/16144737

274. Sun YG, Zan LS, Wang HB, Guo HF, Yang DP, Zhao XL, et al. Insulin Inhibits the Expression of Adiponectin and AdipoR2 mRNA in Cultured Bovine Adipocytes. Asian - Australas J Anim Sci [Internet]. 2009;22(10):1429–36. Available from: http://www.ajas.info/Editor/manuscript/upload/22-187.pdf

275. Brame L a, Considine R V, Yamauchi M, Baron AD, Mather KJ. Insulin and endothelin in the acute regulation of adiponectin in vivo in humans. Obes Res [Internet]. 2005 Mar;13(3):582–8. Available from: http://www.ncbi.nlm.nih.gov/pubmed/15833944

276. Kuboki K, Jiang ZY, Takahara N, Ha SW, Igarashi M, Yamauchi T, et al. Regulation of Endothelial Constitutive Nitric Oxide Synthase Gene Expression in Endothelial Cells and In Vivo : A Specific Vascular Action of Insulin. Circulation [Internet]. 2000 Feb 15 [cited 2013 Jan 21];101(6):676–81. Available from: http://circ.ahajournals.org/cgi/doi/10.1161/01.CIR.101.6.676

277. Steinberg HO, Brechtel G, Johnson a, Fineberg N, Baron a D. Insulin-mediated skeletal muscle vasodilation is nitric oxide dependent. A novel action of insulin to increase nitric oxide release. J Clin Invest [Internet]. 1994 Sep;94(3):1172–9. Available from: http://www.pubmedcentral.nih.gov/articlerender.fcgi?artid=295191&tool=pmcentrez&rendertype=abstract

278. Pascaud X, Ferre J, Genton M, Roger A, Ruckebusch M, Bueno L. Intestinal motility responses to insulin and glucagon in streptozotocin diabetic rats. Can J Physiol Pharmacol [Internet]. 1982;60(7):960–7. Available from: http://www.ncbi.nlm.nih.gov/pubmed/6215115

279. Najafzadeh H, Kooshapur H, Kianidehkordi F. Evaluation of an oral insulin formulation in normal and diabetic rats. Indian J Pharmacol [Internet]. 2012 Jan [cited 2013 Jan 29];44(1):103–5. Available from: http://www.pubmedcentral.nih.gov/articlerender.fcgi?artid=3271511&tool=pmcentrez&rendertype=abstract

280. Dentin R, Liu Y, Koo S-H, Hedrick S, Vargas T, Heredia J, et al. Insulin modulates gluconeogenesis by inhibition of the coactivator TORC2. Nature [Internet]. 2007 Sep 20 [cited 2013 Jan 29];449(7160):366–9. Available from: http://www.ncbi.nlm.nih.gov/pubmed/17805301

281. Pocai A, Lam TKT, Gutierrez-Juarez R, Obici S, Schwartz GJ, Bryan J, et al. Hypothalamic K(ATP) channels control hepatic glucose production. Nature [Internet]. 2005 Apr 21;434(7036):1026–31. Available from: http://www.ncbi.nlm.nih.gov/pubmed/15846348

282. Stump CS, Short KR, Bigelow ML, Schimke JM, Nair KS. Effect of insulin on human skeletal muscle mitochondrial ATP production, protein synthesis, and mRNA transcripts. Proc Natl Acad Sci U S A [Internet]. 2003 Jun 24;100(13):7996–8001. Available from: http://www.pubmedcentral.nih.gov/articlerender.fcgi?artid=164701&tool=pmcentrez&rendertype=abstract

283. Cornish J, Callon K, Reid I. Insulin increases histomorphometric indices of bone formation In vivo. Calcif Tissue Int [Internet]. 1996;59(6):492–5. Available from: http://www.ncbi.nlm.nih.gov/pubmed/8939777

284. Ferron M, Wei J, Yoshizawa T, Fattore A Del, DePinho RA, Teti A, et al. Insulin signaling in osteoblasts integrates bone remodeling and energy metabolism. Cell [Internet]. 2010;142(2):296–308. Available from: http://www.ncbi.nlm.nih.gov/pmc/articles/PMC2910411/pdf/nihms213523.pdf

285. Zhang HH, Huang J, Düvel K, Boback B, Wu S, Squillace RM, et al. Insulin stimulates adipogenesis through the Akt-TSC2-mTORC1 pathway. PLoS One [Internet]. 2009 Jan [cited 2013 Jan 29];4(7):e6189. Available from: http://www.pubmedcentral.nih.gov/articlerender.fcgi?artid=2703782&tool=pmcentrez&rendertype=abstract

286. Coomans CP, Geerling JJ, Guigas B, van den Hoek AM, Parlevliet ET, Ouwens DM, et al. Circulating insulin stimulates fatty acid retention in white adipose tissue via K-ATP channel activation in the central nervous system only in insulin-sensitive mice. J Lipid Res [Internet]. 2011 Sep [cited 2013 Jan 29];52(9):1712–22. Available from: http://www.pubmedcentral.nih.gov/articlerender.fcgi?artid=3151691&tool=pmcentrez&rendertype=abstract

287. Iwai M, Yoshino G, T K, Matsuba K, Matsushita M, Iwatani I, et al. The role of insulin in triglyceride turnover in rats. Diabetes Res Clin Pract [Internet]. 1989;7:S115-8. Available from: http://www.ncbi.nlm.nih.gov/pubmed/2806054

288. Marbani SL, Roth J. Transgenic hyperinsulinemia : A mouse model of insulin resistance and glucose intolerance without obesity. In: Shafrir E, editor. Lessons from Animal Diabetes VI. VI. Birkhäuser Basel; 1996. p. 420.

289. Ruderman BNB, Ross PS, Berger M, Goodman MN. Regulation of Glucose and Ketone-Body Metabolism in Brain of Anaesthetized Rats. Biochem J [Internet]. 1974;138(1):1–10. Available from: http://www.ncbi.nlm.nih.gov/pubmed/4275704

290. Lenzen S, Schmidt W, Rustenbeck I, Panten U. 3-Ketoglutarate generation in pancreatic B-cell mitochondria regulates insulin secretory action of amino acids and 2-keto acids. Biosci Rep [Internet]. 1986;6(2):163–9. Available from: http://link.springer.com/article/10.1007/BF01115002?LI=true

291. Hutton JC, Sener A, Malaisse WJ. Interaction of branched chain amino acids and keto acids upon pancreatic islet metabolism and insulin secretion. J Biol Chem [Internet]. 1980 Aug 10;255(15):7340–6. Available from: http://www.ncbi.nlm.nih.gov/pubmed/6993486

292. Andrae U, Singh J, Ziegler-Skylakakis K. Pyruvate and related alpha-ketoacids protect mammalian cells in culture against hydrogen peroxide-induced cytotoxicity. Toxicol Lett [Internet]. 1985;28(2–3):93–8. Available from: http://www.ncbi.nlm.nih.gov/pubmed/4071565

293. Ohnishi M, Razzaque MS. Dietary and genetic evidence for phosphate toxicity accelerating mammalian aging. FASEB J [Internet]. 2010 Sep [cited 2013 Feb 21];24(9):3562–71. Available from: http://www.pubmedcentral.nih.gov/articlerender.fcgi?artid=2923352&tool=pmcentrez&rendertype=abstract

294. Kuro-o M, Matsumura Y, Aizawa H, Kawaguchi H, Suga T, Utsugi T, et al. Mutation of the mouse klotho gene leads to a syndrome resembling ageing. Nature [Internet]. 1997 Nov 6;390(6655):45–51. Available from: http://www.ncbi.nlm.nih.gov/pubmed/9363890

295. Ohnishi M, Kato S, Akiyoshi J, Atfi A, Razzaque MS. Dietary and genetic evidence for enhancing glucose metabolism and reducing obesity by inhibiting klotho functions. FASEB J [Internet]. 2011 Jun [cited 2013 Jan 30];25(6):2031–9. Available from: http://www.pubmedcentral.nih.gov/articlerender.fcgi?artid=3101030&tool=pmcentrez&rendertype=abstract

296. Kurosu H, Yamamoto M, Clark JD, Pastor J V, Nandi A, Gurnani P, et al. Suppression of Aging in Mice by the hormone Klotho. Science (80- ) [Internet]. 2005;309(5742):1829–33. Available from: http://www.ncbi.nlm.nih.gov/pmc/articles/PMC2536606/pdf/nihms-58249.pdf

297. Yamamoto M, Clark JD, Pastor J V, Gurnani P, Nandi A, Kurosu H, et al. Regulation of oxidative stress by the anti-aging hormone klotho. J Biol Chem [Internet]. 2005 Nov 11 [cited 2013 Jan 30];280(45):38029–34. Available from: http://www.pubmedcentral.nih.gov/articlerender.fcgi?artid=2515369&tool=pmcentrez&rendertype=abstract

298. Shimada T, Takeshita Y, Murohara T, Sasaki K, Egami K, Shintani S, et al. Angiogenesis and vasculogenesis are impaired in the precocious-aging klotho mouse. Circulation [Internet]. 2004 Aug 31 [cited 2013 Jan 30];110(9):1148–55. Available from: http://www.ncbi.nlm.nih.gov/pubmed/15302783

299. Liu F, Wu S, Ren H, Gu J. Klotho suppresses RIG-I-mediated senescence-associated inflammation. Nat Cell Biol [Internet]. 2011 Mar [cited 2013 Jan 30];13(3):254–62. Available from: http://www.ncbi.nlm.nih.gov/pubmed/21336305

300. Zhao Y, Banerjee S, Dey N, LeJeune WS, Sarkar PS, Brobey R, et al. Klotho depletion contributes to increased inflammation in kidney of the db/db mouse model of diabetes via RelA (serine)536 phosphorylation. Diabetes [Internet]. 2011 Jul [cited 2013 Jan 30];60(7):1907–16. Available from: http://www.pubmedcentral.nih.gov/articlerender.fcgi?artid=3121423&tool=pmcentrez&rendertype=abstract

301. Chihara Y, Rakugi H, Ishikawa K, Ikushima M, Maekawa Y, Ohta J, et al. Klotho protein promotes adipocyte differentiation. Endocrinology [Internet]. 2006 Aug [cited 2013 Jan 30];147(8):3835–42. Available from: http://www.ncbi.nlm.nih.gov/pubmed/16709611

302. Lin Y, Sun Z. Antiaging gene Klotho enhances glucose-induced insulin secretion by up-regulating plasma membrane levels of TRPV2 in MIN6 β-cells. Endocrinology [Internet]. 2012;153(7):3029–39. Available from: http://www.ncbi.nlm.nih.gov/pubmed/22597535

303. Elias CF, Lee C, Kelly J, Aschkenasi C, Ahima RS, Couceyro PR, et al. Leptin Activates Hypothalamic CART Neurons Projecting to the Spinal Cord. Neuron [Internet]. 1998;21(6):1375–85. Available from: http://www.sciencedirect.com/science/article/pii/S089662730080656X#

304. Schulz C, Paulus K, Jöhren O, Lehnert H. Intranasal leptin reduces appetite and induces weight loss in rats with diet-induced obesity (DIO). Endocrinology [Internet]. 2012 Jan [cited 2013 Jan 17];153(1):143–53. Available from: http://www.ncbi.nlm.nih.gov/pubmed/22128019

305. Lee Y, Wang M-Y, Kakuma T, Wang Z-W, Babcock E, McCorkle K, et al. Liporegulation in diet-induced obesity. The antisteatotic role of hyperleptinemia. J Biol Chem [Internet]. 2001 Mar 23 [cited 2012 Oct 31];276(8):5629–35. Available from: http://www.ncbi.nlm.nih.gov/pubmed/11096093

306. Bouloumie A, Drexler HC a., Lafontan M, Busse R. Leptin, the Product of Ob Gene, Promotes Angiogenesis. Circ Res [Internet]. 1998 Nov 16 [cited 2013 Feb 9];83(10):1059–66. Available from: http://circres.ahajournals.org/cgi/doi/10.1161/01.RES.83.10.1059

307. Park H-Y, Kwon HM, Lim HJ, Hong BK, Lee JY, Park BE, et al. Potential role of leptin in angiogenesis: leptin induces endothelial cell proliferation and expression of matrix metalloproteinases in vivo and in vitro. Exp Mol Med [Internet]. 2001 Jun 30;33(2):95–102. Available from: http://www.ncbi.nlm.nih.gov/pubmed/11460888

308. Sierra-Honigmann MR, Nath AK, Murakami C, Garcõ«a-Carden÷a G, Papapetropoulos A, Sessa WC, et al. Biological Action of Leptin as an Angiogenic Factor. Science (80- ) [Internet]. 1998 Sep 11 [cited 2013 Feb 9];281(5383):1683–6. Available from: http://www.sciencemag.org/cgi/doi/10.1126/science.281.5383.1683

309. Anagnostoulis S, Karayiannakis A, Lambropoulou M, Efthimiadou A, Polychronidis A, Simopoulos C. Human leptin induces angiogenesis in vivo. Cytokine [Internet]. 2008;42(3):353–7. Available from: http://www.ncbi.nlm.nih.gov/pubmed/18448353

310. Farr SA, Banks WA, Morley JE. Effects of leptin on memory processing. Peptides [Internet]. 2006;27(6):1420–5. Available from: http://www.sciencedirect.com/science/article/pii/S0196978105004754

311. Calapai G, Corica F, Corsonello A, Sautebin L, Rosa M Di, Campo GM, et al. Leptin increases serotonin turnover by inhibition of brain nitric oxide synthesis. J Clin Invest [Internet]. 1999;104(7):975–82. Available from: http://www.ncbi.nlm.nih.gov/pubmed/10510338

312. Yadav VK, Oury F, Suda N, Liu Z, Gao X, Klemenhagen KC, et al. Leptin regulation of bone mass, appetite and energy expenditure relies on its ability to inhibit serotonin synthesis in the brainstem. Cell [Internet]. 2010;138(5):976–89. Available from: http://www.ncbi.nlm.nih.gov/pmc/articles/PMC2768582/

313. Zeidan A, Purdham DM, Rajapurohitam V, Javadov S, Chakrabarti S, Karmazyn M. Leptin Induces Vascular Smooth Muscle Cell Hypertrophy through Angiotensin II- and Endothelin-1-Dependent Mechanisms and Mediates Stretch-Induced Hypertrophy. J Pharmacol Exp Ther [Internet]. 2005;315(3):1075–84. Available from: http://jpet.aspetjournals.org/content/315/3/1075.full.pdf

314. Quehenberger P, Exner M, Sunder-Plassmann R, Ruzicka K, Bieglmayer C, Endler G, et al. Leptin Induces Endothelin-1 in Endothelial Cells In Vitro. Circ Res [Internet]. 2002 Feb 28 [cited 2013 Jan 17];90(6):711–8. Available from: http://circres.ahajournals.org/cgi/doi/10.1161/01.RES.0000014226.74709.90

315. Jiang L, Wang Q, Yu Y, Zhao F, Huang P, Zeng R, et al. Leptin contributes to the adaptive responses of mice to high-fat diet intake through suppressing the lipogenic pathway. PLoS One [Internet]. 2009 Jan [cited 2013 Jan 4];4(9):e6884. Available from: http://www.pubmedcentral.nih.gov/articlerender.fcgi?artid=2731220&tool=pmcentrez&rendertype=abstract

316. Shimabukuro M, Koyama K, Chen G, Wang M-Y, Trieu F, Lee Y, et al. Direct antidiabetic effect of leptin through triglyceride depletion of tissues. Proc Natl Acad Sci U S A [Internet]. 1997 Apr 29;94(9):4637–41. Available from: http://www.pubmedcentral.nih.gov/articlerender.fcgi?artid=20776&tool=pmcentrez&rendertype=abstract

317. German JP, Wisse BE, Thaler JP, Oh-i S, Sarruf DA, Ogimoto K, et al. Leptin Deficiency Causes Insulin Resistance Induced by Uncontrolled Diabetes. Diabetes [Internet]. 2010;59(July):1626–34. Available from: http://www.ncbi.nlm.nih.gov/pmc/articles/PMC2889761/pdf/zdb1626.pdf

318. Lin C-Y, Higginbotham DA, Judd RL, White BD. Central leptin increases insulin sensitivity in streptozotocin-induced diabetic rats. Am J Physiol Endocrinol Metab [Internet]. 2002;282(5):E1084–91. Available from: http://www.ncbi.nlm.nih.gov/pubmed/11934674

319. Covey SD, Wideman RD, McDonald C, Unniappan S, Huynh F, Asadi A, et al. The pancreatic beta cell is a key site for mediating the effects of leptin on glucose homeostasis. Cell Metab [Internet]. 2006 Oct [cited 2014 May 27];4(4):291–302. Available from: http://www.ncbi.nlm.nih.gov/pubmed/17011502

320. Jasnow AM, Huhman KL, Bartness TJ, Demas GE. Short Days and Exogenous Melatonin Increase Aggression of Male Syrian Hamsters (Mesocricetus auratus). Horm Behav [Internet]. 2002 Aug [cited 2013 Feb 5];42(1):13–20. Available from: http://linkinghub.elsevier.com/retrieve/pii/S0018506X02917976

321. Roth JA, Kim B-G, Lin W-L, Cho M-I. Melatonin promotes osteoblast differentiation and bone formation. J Biol Chem [Internet]. 1999 Jul 30;274(31):22041–7. Available from: http://www.ncbi.nlm.nih.gov/pubmed/10419530

322. Sanchez-Hidalgo M, Lu Z, Tan D-X, Maldonado MD, Reiter RJ, Gregerman RI. Melatonin inhibits fatty acid-induced triglyceride accumulation in ROS17/2.8 cells: implications for osteoblast differentiation and osteoporosis. Am J Physiol Regul Integr Comp Physiol [Internet]. 2007 Jun [cited 2013 Feb 5];292(6):R2208-15. Available from: http://www.ncbi.nlm.nih.gov/pubmed/17379847

323. Puchalski SS, Green JN, Rasmussen DD. Melatonin effect on rat body weight regulation in response to high-fat diet at middle age. Endocrine [Internet]. 2003 Jul;21(2):163–7. Available from: http://www.ncbi.nlm.nih.gov/pubmed/12897381

324. Rasmussen DD, Boldt BM, Wilkinson CW, Yellon SM, Matsumoto AM. Daily melatonin administration at middle age suppresses male rat visceral fat, plasma leptin and plasma insulin to youthful levls. Endocrinology [Internet]. 1999;140(2):1009–12. Available from: http://endo.endojournals.org/content/140/2/1009.long

325. Prunet-Marcassus B, Desbazeille M, Bros A, Louche K, Delagrange P, Renard P, et al. Melatonin reduces body weight gain in Sprague Dawley rats with diet-induced obesity. Endocrinology [Internet]. 2003 Dec [cited 2013 Feb 5];144(12):5347–52. Available from: http://www.ncbi.nlm.nih.gov/pubmed/12970162

326. Wolden-Hanson T, Mitton DR, McCants RL, Yellon SM, Wilkinson CW, Matsumoto a M, et al. Daily melatonin administration to middle-aged male rats suppresses body weight, intraabdominal adiposity, and plasma leptin and insulin independent of food intake and total body fat. Endocrinology [Internet]. 2000 Feb;141(2):487–97. Available from: http://www.ncbi.nlm.nih.gov/pubmed/10650927

327. Nishida S, Segawa T, Murai I, Nakagawa S. Long-term melatonin administration reduces hyperinsulinemia and improves the altered fatty-acid compositions in type 2 diabetic rats via the restoration of Delta-5 desaturase activity. J Pineal Res [Internet]. 2002;32(1):26–33. Available from: http://www.ncbi.nlm.nih.gov/pubmed/11841597

328. de Oliveira AC, Andreotti S, Farias T da SM, Torres-Leal FL, Proença ARG de, Campaña AB, et al. Metabolic Disorders and Adipose Tissue Insulin Responsiveness in Neonatally STZ-Induced Diabetic Rats Are Improved by Long-Term Melatonin Treatment. Endocrinology [Internet]. 2012;153(5):2178–88. Available from: http://endo.endojournals.org/content/153/5/2178

329. Cuesta S, Kireev R, García C, Rancan L, Vara E, Tresguerres JAF. Melatonin can improve insulin resistance and aging-induced pancreas alterations in senescence-accelerated prone male mice (SAMP8). Age (Dordr) [Internet]. 2012 Mar 13 [cited 2013 Jan 17];1–13. Available from: http://www.ncbi.nlm.nih.gov/pubmed/22411259

330. Sartori C, Dessen P, Mathieu C, Monney A, Bloch J, Nicod P, et al. Melatonin improves glucose homeostasis and endothelial vascular function in high-fat diet-fed insulin-resistant mice. Endocrinology [Internet]. 2009 Dec [cited 2013 Jan 17];150(12):5311–7. Available from: http://www.ncbi.nlm.nih.gov/pubmed/19819971

331. Contreras-Alcantara S, Baba K, Tosini G. Removal of melatonin receptor type 1 induces insulin resistance in the mouse. Obesity (Silver Spring) [Internet]. Nature Publishing Group; 2010 Sep [cited 2013 Jan 17];18(9):1861–3. Available from: http://www.pubmedcentral.nih.gov/articlerender.fcgi?artid=2929321&tool=pmcentrez&rendertype=abstract

332. Zanuto R, Siqueira-Filho MA, Caperuto LC, Bacurau RFP, Hirata E, Peliciari-Garcia RA, et al. Melatonin Improves Insulin Sensitivity Independently Of Weight Loss In Old Obese Rats. J Pineal Res [Internet]. 2013; Available from: http://onlinelibrary.wiley.com/doi/10.1111/jpi.12056/abstract

333. Seo H, Park K, Park S, Javaregowda PK, Hong Y. Melatonin prevents muscle atrophy and the alteration of myosin heavy chain in spinal cord injured rats. J Fed Am Soc Exp Biol [Internet]. 2010;IB668. Available from: http://www.fasebj.org/cgi/content/meeting_abstract/24/1_MeetingAbstracts/lb668

334. Lee S, Shin J, Hong Y, Lee M, Kim K, Lee S-R, et al. Beneficial effects of melatonin on stroke-induced muscle atrophy in focal cerebral ischemic rats. Lab Anim Res [Internet]. 2012;28(1):47–54. Available from: http://www.ncbi.nlm.nih.gov/pmc/articles/PMC3315192/

335. Heijden G Van Der, Wang ZJ, Chu Z, Toffolo G, Manesso E, Sauer PJJ, et al. Strength Exercise Improves Muscle Mass and Hepatic Insulin Sensitivity in Obese Youth. Med Sci Sports Exerc [Internet]. 2010;42(11):1973–80. Available from: http://www.ncbi.nlm.nih.gov/pmc/articles/PMC2944907/pdf/nihms203558.pdf

336. Wagner KR, Liu X, Chang X, Allen RE. Muscle regeneration in the prolonged absence of myostatin. Proc Natl Acad Sci U S A [Internet]. 2005 Mar 15;102(7):2519–24. Available from: http://www.pubmedcentral.nih.gov/articlerender.fcgi?artid=548322&tool=pmcentrez&rendertype=abstract

337. Lee S-J, McPherron AC. Regulation of myostatin activity and muscle growth. Proc Natl Acad Sci U S A [Internet]. 2001 Jul 31;98(16):9306–11. Available from: http://www.pubmedcentral.nih.gov/articlerender.fcgi?artid=55416&tool=pmcentrez&rendertype=abstract

338. Lee S-J. Quadrupling muscle mass in mice by targeting TGF-beta signaling pathways. PLoS One [Internet]. 2007 Jan [cited 2013 Jan 30];2(8):e789. Available from: http://www.pubmedcentral.nih.gov/articlerender.fcgi?artid=1949143&tool=pmcentrez&rendertype=abstract

339. Guo T, Jou W, Chanturiya T, Portas J, Gavrilova O, McPherron AC. Myostatin inhibition in muscle, but not adipose tissue, decreases fat mass and improves insulin sensitivity. PLoS One [Internet]. 2009 Jan [cited 2013 Jan 30];4(3):e4937. Available from: http://www.pubmedcentral.nih.gov/articlerender.fcgi?artid=2654157&tool=pmcentrez&rendertype=abstract

340. Amirouche A, Durieux A-C, Banzet S, Koulmann N, Bonnefoy R, Mouret C, et al. Down-regulation of Akt/mammalian target of rapamycin signaling pathway in response to myostatin overexpression in skeletal muscle. Endocrinology [Internet]. 2009 Jan [cited 2013 Feb 16];150(1):286–94. Available from: http://www.ncbi.nlm.nih.gov/pubmed/18801898

341. Mcpherron AC, Lee S. Suppression of body fat accumulation in myostatin-deficient mice. J Clin Invest [Internet]. 2002;109(5):595–601. Available from: http://www.ncbi.nlm.nih.gov/pmc/articles/PMC150888/pdf/JCI0213562.pdf

342. Zhang C, McFarlane C, Lokireddy S, Bonala S, Ge X, Masuda S, et al. Myostatin-deficient mice exhibit reduced insulin resistance through activating the AMP-activated protein kinase signalling pathway. Diabetologia [Internet]. 2011 Jun [cited 2013 Jan 30];54(6):1491–501. Available from: http://www.ncbi.nlm.nih.gov/pubmed/21347623

343. Hittel DS, Axelson M, Sarna N, Shearer J, Huffman KM, Kraus WE. Myostatin Decreases with Aerobic Exercise and Associates with Insulin Resistance. Med Sci Sports Exerc [Internet]. 2010;42(11):2023–9. Available from: http://www.ncbi.nlm.nih.gov/pmc/articles/PMC2975387/pdf/nihms203559.pdf

344. Wilkes JJ, Lloyd DJ, Gekakis N. Loss-of-Function Mutation in Myostatin Reduces Tumor Necrosis Factor alpha Production and Protects Liver Against Obesity-Induced Insulin Resistance. Diabetes [Internet]. 2009;58(May):1133–43. Available from: http://diabetes.diabetesjournals.org/content/58/5/1133.full.pdf

345. Michael GJ, Averill S, Nitkunan A, Rattray M, Bennett DLH, Yan Q, et al. Nerve growth factor treatment increases brain-derived neurotrophic factor selectively in TrkA-expressing dorsal root ganglion cells and in their central terminations within the spinal cord. J Neurosci [Internet]. 1997 Nov 1;17(21):8476–90. Available from: http://www.ncbi.nlm.nih.gov/pubmed/9334420

346. Gearing DP, Virtue ER, Gearing RP, Drew AC. A fully caninised anti-NGF monoclonal antibody for pain relief in dogs A fully caninised anti-NGF monoclonal antibody for pain relief in dogs. BMC Vet Res [Internet]. 2013;9(226). Available from: http://www.ncbi.nlm.nih.gov/pubmed/24206926

347. Shelton D, Zeller J, Ho W, Pons J, Rosenthal A. Nerve growth factor mediates hyperalgesia and cachexia in auto-immune arthritis. Pain [Internet]. 2005;116(1–2):8–16. Available from: http://www.ncbi.nlm.nih.gov/pubmed/15927377

348. Hirth M, Rukwied R, Gromann A, Turnquist B, Weinkauf B, Francke K, et al. Nerve growth factor induces sensitization of nociceptors without evidence for increased intraepidermal nerve fiber density. Pain [Internet]. 2013;154(11):2500–11. Available from: http://www.ncbi.nlm.nih.gov/pubmed/23891896

349. Quyyumi AA, Dakak N, Andrews NP, Gilligan DM, Panza JA, Cannon III ROC. Contribution of Nitric Oxide to Metabolic Coronary Vasodilation in the Human Heart. Circulation [Internet]. 1995;92:320–6. Available from: http://circ.ahajournals.org/content/92/3/320.full

350. Lee PC, Salyapongse AN, Bragdon GA, Shears II LL, Watkins SC, Edington HDJ, et al. Impaired wound healing and angiogenesis in eNOS-deficient mice. Am J Physiol - Hear Circ Physiol [Internet]. 1999;277:H1600-8. Available from: http://ajpheart.physiology.org/content/277/4/H1600.full.pdf

351. Seilicovich A, Lasaga M, Befumo M, Duvilanski BH, del Carmen Diaz M, Rettori V, et al. Nitric oxide inhibits the release of norepinephrine and dopamine from the medial basal hypothalamus of the rat. Proc Natl Acad Sci U S A [Internet]. 1995 Nov 21;92(24):11299–302. Available from: http://www.pubmedcentral.nih.gov/articlerender.fcgi?artid=40619&tool=pmcentrez&rendertype=abstract

352. Demas GE, Kriegsfeld LJ, Blackshaw S, Huang P, Gammie SC, Nelson RJ, et al. Elimination of Aggressive Behavior in Male Mice Lacking Endothelial Nitric Oxide Synthase. J Neurosci [Internet]. 1999;19(19):2–6. Available from: http://www.ncbi.nlm.nih.gov/pubmed/10493775

353. Gammie SC, Nelson RJ. Maternal aggression is reduced in neuronal nitric oxide synthase-deficient mice. J Neurosci [Internet]. 1999 Sep 15;19(18):8027–35. Available from: http://www.ncbi.nlm.nih.gov/pubmed/10479702

354. Chiavegatto S, Dawson VL, Mamounas LA, Koliatsos VE, Dawson TM, Nelson RJ. Brain serotonin dysfunction accounts for aggression in male mice lacking neuronal nitric oxide synthase. Proc Natl Acad Sci U S A [Internet]. 2001 Jan 30;98(3):1277–81. Available from: http://www.pubmedcentral.nih.gov/articlerender.fcgi?artid=14745&tool=pmcentrez&rendertype=abstract

355. Kriegsfeld LJ, Dawson TM, Dawson VL, Nelson RJ, Snyder SH. Aggressive behavior in male mice lacking the gene for neuronal nitric oxide synthase requires testosterone. Brain Res [Internet]. 1997;769(1):66–70. Available from: http://www.ncbi.nlm.nih.gov/pubmed/9374274

356. Demas GE, Eliasson MJ, Dawson TM, Dawson VL, Kriegsfeld LJ, Nelson RJ, et al. Inhibition of neuronal nitric oxide synthase increases aggressive behavior in mice. Mol Med [Internet]. 1997 Sep;3(9):610–6. Available from: http://www.pubmedcentral.nih.gov/articlerender.fcgi?artid=2230093&tool=pmcentrez&rendertype=abstract

357. Nelson R, Demas G, Huang P, Fishman M, Dawson V, Dawson T, et al. Behavioural abnormalities in male mice lacking neuronal nitric oxide synthase. Nature [Internet]. 1995;378(6555):383–6. Available from: http://www.ncbi.nlm.nih.gov/pubmed/7477374

358. Luo S, Luo J, Cincotta A. Chronic ventromedial hypothalamic infusion of norepinephrine and serotonin promotes insulin resistance and glucose intolerance. Neuroendocrinology [Internet]. 1999;70(6):460–5. Available from: http://www.ncbi.nlm.nih.gov/pubmed/10657739

359. Itoi K, Suda T, Tozawa F, Dobashi I, Ohmori N, Sakai Y, et al. Microinjection of norepinephrine into the paraventricular nucleus of the hypothalamus stimulates corticotropin-releasing factor gene expression in conscious rats. Endocrinology [Internet]. 1994;135(5):2177–82. Available from: http://www.ncbi.nlm.nih.gov/pubmed/7956940

360. Veldhuis JD, Evans WS, Bowers CY. Estradiol Supplementation Enhances Submaximal Feed-Forward Drive of Growth Hormone (GH) Secretion by Recombinant Human GH-Releasing Hormone-1,44-Amide in a Putatively Somatostatin-Withdrawn Milieu. J Clin Endocrinol Metab [Internet]. 2003 Nov 1 [cited 2013 Jan 16];88(11):5484–9. Available from: http://jcem.endojournals.org/cgi/doi/10.1210/jc.2003-030410

361. Slootweg MC, Swolin D, Netelenbos JC, Isaksson OGP, Ohlsson C. Estrogen enhances growth hormone receptor expression and growth hormone action in rat osteosarcoma cells and human osteoblast-like cells. J Endocrinol [Internet]. 1997 Oct;155(1):159–64. Available from: http://www.ncbi.nlm.nih.gov/pubmed/9390018

362. Moll GJ, Rosenfield R, Fang V. Administration of low-dose estrogen rapidly and directly stimulates growth hormone production. Am J Dis Child [Internet]. 1986;140(2):124–7. Available from: http://www.ncbi.nlm.nih.gov/pubmed/3946321

363. Andersson B, Mattsson L-Å, Hahn L, MÅrin P, Lapidus L, Holm G, et al. Estrogen replacement therapy decreases hyperandrogenicity and improves glucose homeostasis and plasma lipids in postmenopausal women with noninsulin-dependent diabetes mellitus. J Clin Endocrinol Metab [Internet]. 1997;82(2):638–43. Available from: http://www.ncbi.nlm.nih.gov/pubmed/9024268

364. Kumagai S, Holmäng A, Björntorp P. The effects of oestrogen and progesterone on insulin sensitivity in female rats. Acta Physiol Scand [Internet]. 1993;149(1):91–7. Available from: http://www.ncbi.nlm.nih.gov/pubmed/8237427

365. Godsland I, Walton C, Felton C, Proudler A, Patel A, Wynn V. Insulin resistance, secretion, and metabolism in users of oral contraceptives. J Clin Endocrinol Metab [Internet]. 1992;74(1):64–70. Available from: http://www.ncbi.nlm.nih.gov/pubmed/1530790

366. Musatov S, Chen W, Pfaff DW, Mobbs C V, Yang X, Clegg DJ, et al. Silencing of estrogen receptor α in the ventromedial nucleus of hypothalamus leads to metabolic syndrome. Proc Natl Acad Sci U S A [Internet]. 2007;104(7):2501–6. Available from: http://www.ncbi.nlm.nih.gov/pmc/articles/PMC1892990/

367. Albert D, Jonik R, Walsh M. Interaction of estradiol, testosterone, and progesterone in the modulation of hormone-dependent aggression in the female rat. Physiol Behav [Internet]. 1992;52(4):773–9. Available from: http://www.ncbi.nlm.nih.gov/pubmed/1409952

368. Albert D, Jonik R, Walsh M. Hormone-dependent aggression in the female rat: testosterone plus estradiol implants prevent the decline in aggression following ovariectomy. Physiol Behav [Internet]. 1991;49(4):673–7. Available from: http://www.ncbi.nlm.nih.gov/pubmed/1881968

369. Trainor BC, Finy MS, Nelson RJ. Rapid effects of estradiol on male aggression depend on photoperiod in reproductively non-responsive mice. Horm Behav [Internet]. 2008;53(1):192–9. Available from: http://www.ncbi.nlm.nih.gov/pmc/articles/PMC2190085/

370. Trainor BC, Greiwe KM, Nelson RJ. Individual differences in estrogen receptor α in select brain nuclei are associated with individual differences in aggression. Horm Behav [Internet]. 2006;50(2):338–45. Available from: http://www.ncbi.nlm.nih.gov/pmc/articles/PMC1950320/pdf/nihms11203.pdf

371. Ogawa S, Eng V, Taylor J, Lubahn DB, Korach KS, Pfaff DW. Roles of Estrogen Receptor-alpha Gene Expression in Reproduction-Related Behaviors in Female Mice. Endocrinology [Internet]. 1998;139(12):5070–81. Available from: http://www.ncbi.nlm.nih.gov/pubmed/9832446

372. Ogawa S, Lubahn DB, Korach KS, Pfaff DW. Behavioral effects of estrogen receptor gene disruption in male mice. Proc Natl Acad Sci U S A [Internet]. 1997;94(February):1476–81. Available from: http://www.pnas.org/content/94/4/1476.full.pdf

373. Ogawa S, Chester AE, Hewitt SC, Walker VR, Gustafsson J-åke, Smithies O, et al. Abolition of male sexual behaviors in mice lacking estrogen receptors α and β (αβERKO ). Proc Natl Acad Sci U S A [Internet]. 2000;97(26):14737–41. Available from: http://www.pnas.org/content/97/26/14737.full.pdf

374. Albrecht ED, Babischkin JS, Yaron L, Anderson LD, Udoff LC, Pepe GJ. Effect of estrogen on angiogenesis in co-cultures of human endometrial cells and microvascular endothelial cells. Hum Reprod [Internet]. 2003 Oct 1 [cited 2013 Jan 16];18(10):2039–47. Available from: http://www.humrep.oupjournals.org/cgi/doi/10.1093/humrep/deg415

375. Seo KH, Lee H-S, Jung B, Ko H-M, Choi J-H, Park SJ, et al. Estrogen enhances angiogenesis through a pathway involving platelet-activating factor-mediated nuclear factor-kappaB activation. Cancer Res [Internet]. 2004 Sep 15 [cited 2013 Jan 16];64(18):6482–8. Available from: http://www.ncbi.nlm.nih.gov/pubmed/15374958

376. Dabrosin C, Palmer K, Muller W, Gauldie J. Estradiol promotes growth and angiogenesis in polyoma middle T transgenic mouse mammary tumor explants. Breast Cancer Res Treat [Internet]. 2003;78(1):1–6. Available from: http://www.ncbi.nlm.nih.gov/pubmed/12611451

377. Morales DE, McGowan KA, Grant DS, Maheshwari S, Bhartiya D, Cid MC, et al. Estrogen Promotes Angiogenic Activity in Human Umbilical Vein Endothelial Cells In Vitro and in a Murine Model. Circulation [Internet]. 1995;91:755–63. Available from: http://circ.ahajournals.org/content/91/3/755.long

378. Mooradian A. Antioxidant properties of steroids. J Steroid Biochem Mol Biol [Internet]. 1993;45(6):509–11. Available from: http://www.ncbi.nlm.nih.gov/pubmed/8518206

379. Hogan AM, Kennelly R, Collins D, Baird AW, Winter DC. Oestrogen inhibits human colonic motility by a non-genomic cell membrane receptor-dependent mechanism. Br J Surg [Internet]. 2009;96(7):817–22. Available from: http://onlinelibrary.wiley.com/doi/10.1002/bjs.6612/abstract;jsessionid=F593FE73B985C4960EC1C77BEBED4079.f03t04

380. Falahati-nini A, Riggs BL, Atkinson EJ, Fallon WMO, Eastell R, Khosla S. Relative contributions of testosterone and estrogen in regulating bone resorption and formation in normal elderly men. J Clin Invest [Internet]. 2000;106(12):1553–60. Available from: http://www.ncbi.nlm.nih.gov/pmc/articles/PMC381474/pdf/JCI0010942.pdf

381. Gill-Sharm MK, Gopalkrishnan K, Balasinor N, Parte P, Jayaraman S, Juneja HS. Effects of tamoxifen the fertility of male rats. J Reprod Fertil [Internet]. 1993;99:395–402. Available from: http://www.reproduction-online.org/content/99/2/395.full.pdf

382. Kasai R, Bianco P, Robey P, Kahn A. Production and characterization of an antibody against the human bone GLA protein (BGP/osteocalcin) propeptide and its use in immunocytochemistry of bone cells. J Bone Miner Res [Internet]. 1994;25(3):167–82. Available from: http://www.ncbi.nlm.nih.gov/pubmed/8086856

383. Lee NK, Sowa H, Hinoi E, Ferron M, Ahn JD, Confavreux C, et al. Endocrine regulation of energy metabolism by the skeleton. Cell [Internet]. 2007 Aug 10;130(3):456–69. Available from: http://www.pubmedcentral.nih.gov/articlerender.fcgi?artid=2013746&tool=pmcentrez&rendertype=abstract

384. Ferron M, Hinoi E, Karsenty G, Ducy P. Osteocalcin differentially regulates beta cell and adipocyte gene expression and affects the development of metabolic diseases in wild-type mice. Proc Natl Acad Sci U S A [Internet]. 2008 Apr 1;105(13):5266–70. Available from: http://www.pubmedcentral.nih.gov/articlerender.fcgi?artid=2278202&tool=pmcentrez&rendertype=abstract

385. Rached M, Kode A, Silva BC, Jung DY, Gray S, Ong H, et al. FoxO1 expression in osteoblasts regulates glucose homeostasis through regulation of osteocalcin in mice. J Clin Invest [Internet]. 2010;120(1):357–68. Available from: http://static.jci.org/content_assets/manuscripts/39000/39901/JCI39901.v2.pdf

386. Oury F, Sumara G, Sumara O, Ferron M, Chang H, Smith CE, et al. Endocrine regulation of male fertility by the skeleton. Cell [Internet]. Elsevier Inc.; 2011 Mar 4 [cited 2013 Mar 1];144(5):796–809. Available from: http://www.pubmedcentral.nih.gov/articlerender.fcgi?artid=3052787&tool=pmcentrez&rendertype=abstract

387. Oury F, Ferron M, Huizhen W, Confavreux C, Xu L, Lacombe J, et al. Osteocalcin regulates murine and human fertility through a pancreas-bone-testis axis. J Clin Invest [Internet]. 2013;123(6):2421–33. Available from: http://ironport.iiserpune.ac.in/cgi-bin/patience.cgi?id=78a10b4d-7471-4158-86f7-9ac0b3519abe

388. Mizokami A, Yasutake Y, Gao J, Matsuda M, Takahashi I, Takeuchi H. Osteocalcin Induces Release of Glucagon-Like Peptide-1 and Thereby Stimulates Insulin Secretion in Mice. PLoS One [Internet]. 2013;8(2):1–8. Available from: http://www.ncbi.nlm.nih.gov/pubmed/23437377

389. Bosch OJ, Meddle SL, Beiderbeck DI, Douglas AJ, Neumann ID. Brain oxytocin correlates with maternal aggression: link to anxiety. J Neurosci [Internet]. 2005 Jul 20 [cited 2013 Feb 20];25(29):6807–15. Available from: http://www.ncbi.nlm.nih.gov/pubmed/16033890

390. De Vries A, Young 3rd W, Nelson R. Reduced aggressive behaviour in mice with targeted disruption of the oxytocin gene. J Neuroendocrinol [Internet]. 1997;9(5):363–8. Available from: http://www.ncbi.nlm.nih.gov/pubmed/9181490

391. Bales KL, Carter CS. Sex differences and developmental effects of oxytocin on aggression and social behavior in prairie voles (Microtus ochrogaster). Horm Behav [Internet]. 2003 Sep [cited 2013 Feb 20];44(3):178–84. Available from: http://linkinghub.elsevier.com/retrieve/pii/S0018506X03001545

392. Calcagnoli F, Boer SF De, Althaus M, Boer JA den, Koolhaas JM. Antiaggressive activity of central oxytocin in male rats. Psychopharmacology (Berl) [Internet]. 2013;229(4):639–51. Available from: http://www.ncbi.nlm.nih.gov/pubmed/23624810

393. Muir J, Pfister H. Influence of exogenously administered oxytocin on the corticosterone and prolactin response to psychological stress. Pharmacol Biochem Behav [Internet]. 1988;29(4):699–703. Available from: http://www.ncbi.nlm.nih.gov/pubmed/3413197

394. Windle RJ, Shanks N, Lightman SL, Ingram CD. Central Oxytocin Administration Reduces Stress- Induced Corticosterone Release and Anxiety Behavior in Rats. Endocrinology [Internet]. 1997;138(7):2829–34. Available from: http://www.ncbi.nlm.nih.gov/pubmed/9202224

395. Ditzen B, Schaer M, Gabriel B, Bodenmann G, Ehlert U, Heinrichs M. Intranasal oxytocin increases positive communication and reduces cortisol levels during couple conflict. Biol Psychiatry [Internet]. 2009;65(9):728–31. Available from: http://www.ncbi.nlm.nih.gov/pubmed/19027101

396. Takayanagi Y, Kasahara Y, Onaka T, Takahashi N, Kawada T, Nishimori K. Oxytocin receptor-deficient mice developed late-onset obesity. Neuroreport [Internet]. 2008;19(9):951–5. Available from: http://www.ncbi.nlm.nih.gov/pubmed/18520999

397. Camerino C. Low sympathetic tone and obese phenotype in oxytocin-deficient mice. Obes (Silver Spring) [Internet]. 2009;17(5):980–4. Available from: http://www.ncbi.nlm.nih.gov/pubmed/19247273

398. Kublaoui BM, Gemelli T, Tolson KP, Wang Y, Zinn AR. Oxytocin deficiency mediates hyperphagic obesity of Sim1 haploinsufficient mice. Mol Endocrinol [Internet]. 2008 Jul [cited 2013 Feb 21];22(7):1723–34. Available from: http://www.pubmedcentral.nih.gov/articlerender.fcgi?artid=2453606&tool=pmcentrez&rendertype=abstract

399. Altszuler N, Hampshire J. Oxytocin infusion increases plasma insulin and glucagon levels and glucose production and uptake in the normal dog. Diabetes [Internet]. 1981;30(2):112–4. Available from: http://www.ncbi.nlm.nih.gov/pubmed/7009266

400. Petersson M, Eklund M, Uvnäs-Moberg K. Oxytocin decreases corticosterone and nociception and increases motor activity in OVX rats. Maturitas [Internet]. 2005;51(4):426–33. Available from: http://www.sciencedirect.com/science/article/pii/S0378512204003640

401. Jackson RA, Peters N, Advani U, Perry G, Rogers J, Brough WH, et al. Forearm Glucose Uptake During the Oral Glucose Tolerance Test in Normal Subjects. Diabetes [Internet]. 1973;2(6):4–5. Available from: http://diabetes.diabetesjournals.org/content/22/6/442.short

402. Shiiya T, Nakazato M, Mizuta M, Date Y, Mondal MS, Tanaka M, et al. Plasma ghrelin levels in lean and obese humans and the effect of glucose on ghrelin secretion. J Clin Endocrinol Metab [Internet]. 2002 Jan;87(1):240–4. Available from: http://www.ncbi.nlm.nih.gov/pubmed/11788653

403. van de Ven KCC, van der Graaf M, Tack CJ, Heerschap A, de Galan BE. Steady-state brain glucose concentrations during hypoglycemia in healthy humans and patients with type 1 diabetes. Diabetes [Internet]. 2012 Aug [cited 2015 May 14];61(8):1974–7. Available from: http://www.pubmedcentral.nih.gov/articlerender.fcgi?artid=3402320&tool=pmcentrez&rendertype=abstract

404. Ohneda A, Aguilar-Parada E, Eisentraut AM, Unger RH. Control of Pancreatic Glucagon Secretion by Glucose. Diabetes [Internet]. 1969;18(1):1–10. Available from: http://diabetes.diabetesjournals.org/content/18/1/1.short

405. Katsumata M, Burton K a, Li J, Dauncey MJ. Suboptimal energy balance selectively up-regulates muscle GLUT gene expression but reduces insulin-dependent glucose uptake during postnatal development. FASEB J [Internet]. 1999 Aug;13(11):1405–13. Available from: http://www.ncbi.nlm.nih.gov/pubmed/10428764

406. Cha SH, Wolfgang M, Tokutake Y, Chohnan S, Lane MD. Differential effects of central fructose and glucose on hypothalamic malonyl-CoA and food intake. Proc Natl Acad Sci U S A [Internet]. 2008 Nov 4;105(44):16871–5. Available from: http://www.pubmedcentral.nih.gov/articlerender.fcgi?artid=2579345&tool=pmcentrez&rendertype=abstract

407. Miller C, Martin R, Whitney M, Edwards G. Intracerebroventricular injection of fructose stimulates feeding in rats. Nutr Neurosci [Internet]. 2002;5(5):359–62. Available from: http://www.ncbi.nlm.nih.gov/pubmed/12385599

408. Ouchi N, Higuchi A, Ohashi K, Oshima Y, Gokce N, Shibata R, et al. Sfrp5 is an anti-inflammatory adipokine that modulates metabolic dysfunction in obesity. Science (80- ) [Internet]. 2010;329(5990):454–7. Available from: http://www.ncbi.nlm.nih.gov/pubmed/20558665

409. Hagiwara H, Fujita Y, Ishima T, Kunitachi S, Shirayama Y, Iyo M, et al. Phencyclidine-induced cognitive deficits in mice are improved by subsequent subchronic administration of the antipsychotic drug perospirone: Role of serotonin 5-HT1A receptors. Eur Neuropsycopharmacology [Internet]. 2008;18(6):448–54. Available from: http://www.sciencedirect.com/science/article/pii/S0924977X07002192

410. Sumiyoshi T, Park S, Jayathilake K, Roy A, Ertugrul A, Meltzer HY. Effect of buspirone, a serotonin1A partial agonist, on cognitive function in schizophrenia: A randomized, double-blind, placebo-controlled study. Schizophr Res [Internet]. 2007;95(1–3):158–68. Available from: http://www.sciencedirect.com/science/article/pii/S0920996407002630

411. Sumiyoshi T, Matsui M, Yamashita I, Nohara S, Kurachi M, Uehara T, et al. The effect of tandospirone, a serotonin1A agonist, on memory function in schizophrenia. Biol Psychiatry [Internet]. 2001;49(10):861–8. Available from: http://www.sciencedirect.com/science/article/pii/S0006322300010258

412. Khaliq S, Haider S, Ahmed S, Perveen T, Haleem D. Relationship of brain tryptophan and serotonin in improving cognitive performance in rats. Pak J Pharm Sci [Internet]. 2006;19(1):11–5. Available from: http://www.ncbi.nlm.nih.gov/pubmed/16632446

413. Nagai T, Murai R, Matsui K, Kamei H, Noda Y, Furukawa H, et al. Aripiprazole ameliorates phencyclidine-induced impairment of recognition memory through dopamine D1 and serotonin 5-HT1A receptors. Psychopharmacology (Berl) [Internet]. 2009 Jan [cited 2013 Feb 19];202(1–3):315–28. Available from: http://www.ncbi.nlm.nih.gov/pubmed/18679658

414. Clarke HF, Dalley JW, Crofts HS, Robbins TW, Roberts AC. Cognitive inflexibility after prefrontal serotonin depletion. Science (80- ) [Internet]. 2004 May 7 [cited 2013 Feb 11];304(5672):878–80. Available from: http://www.ncbi.nlm.nih.gov/pubmed/15131308

415. Blazevic S, Colic L, Culig L, Hranilovic D. Anxiety-like behavior and cognitive flexibility in adult rats perinatally exposed to increased serotonin concentrations. Behav Brain Res [Internet]. Elsevier B.V.; 2012 Apr 21 [cited 2013 Feb 19];230(1):175–81. Available from: http://www.ncbi.nlm.nih.gov/pubmed/22342491

416. Chen X, Margolis KJ, Gershon MD, Schwartz GJ, Sze JY. Reduced serotonin reuptake transporter (SERT) function causes insulin resistance and hepatic steatosis independent of food intake. PLoS One [Internet]. 2012;7(3):1–13. Available from: http://journals.plos.org/plosone/article?id=10.1371/journal.pone.0032511

417. Levkovitz Y, Ben-shushan G, Hershkovitz A, Isaac R, Gil-Ad I, Shvartsman D, et al. Antidepressants induce cellular insulin resistance by activation of IRS-1 kinases. Mol Cell Neurosci [Internet]. 2007;36(3):305–12. Available from: http://www.sciencedirect.com/science/article/pii/S1044743107001339

418. Koopmans SJ, Ruis M, Dekker R, Korte M. Surplus dietary tryptophan inhibits stress hormone kinetics and induces insulin resistance in pigs. Physiol Behav [Internet]. Elsevier Inc.; 2009;98(4):402–10. Available from: http://dx.doi.org/10.1016/j.physbeh.2009.07.001

419. Yadav VK, Ryu J, Suda N, Tanaka K, Gingrich JA, Glorieux FH, et al. Lrp5 controls bone formation by inhibiting serotonin synthesis in the duodenum: an entero-bone endocrine axis. Cell [Internet]. 2009;135(5):825–37. Available from: http://www.ncbi.nlm.nih.gov/pmc/articles/PMC2614332/pdf/nihms82000.pdf

420. Haney EM, Chan BKS, Diem SJ, Ensrud KE, Cauley JA, Barrett-Connor E, et al. Association of low bone mineral density with selective serotonin reuptake inhibitor use by older men. Arch Intern Med [Internet]. 2007 Jun 25;167(12):1246–51. Available from: http://www.ncbi.nlm.nih.gov/pubmed/17592097

421. Richards JB, Papaioannou A, Adachi JD, Joseph L, Whitson HE, Prior JC, et al. Effect of selective serotonin reuptake inhibitors on the risk of fracture. Arch Intern Med [Internet]. 2007 Jan 22;167(2):188–94. Available from: http://www.ncbi.nlm.nih.gov/pubmed/17242321

422. Warden S, Nelson I, Fuchs R, Bliziotes M, Turner C. Serotonin (5-hydroxytryptamine) transporter inhibition causes bone loss in adult mice independently of estrogen deficiency. Menopause [Internet]. 2008;15(6):1176–83. Available from: http://www.ncbi.nlm.nih.gov/pubmed/18725867

423. Cleare AJ, Bond AJ. Does central serotonergic function correlate inversely with aggression? A study using d-fenfluramine in healthy subjects. Psychiatry Res [Internet]. 1997;69(2–3):89–95. Available from: http://www.sciencedirect.com/science/article/pii/S0165178196030521

424. Gibbons J, Barr G, Bridger W, Leibowitz S. Manipulations of dietary tryptophan: effects on mouse killing and brain serotonin in the rat. Brain Res [Internet]. 1979;169(1):139–53. Available from: http://www.ncbi.nlm.nih.gov/pubmed/572256

425. Saudou F, Amara D, Dierich A, LeMeur M, Ramboz S, Segu L, et al. Enhanced aggressive behavior in mice lacking 5-HT1B receptor. Science (80- ) [Internet]. 1994;265(5180):1875–8. Available from: http://www.ncbi.nlm.nih.gov/pubmed/8091214

426. Bjork JM, Dougherty DM, Moeller FG, Cherek DR, Swann AC. The effects of tryptophan depletion and loading on laboratory aggression in men: time course and a food-restricted control. Psychopharmacology (Berl) [Internet]. 1999 Feb;142(1):24–30. Available from: http://www.ncbi.nlm.nih.gov/pubmed/10102779

427. LeMarquand DG, Pihl RO, Young SN, Tremblay RE, Séguin JR, Palmour RM, et al. Tryptophan depletion, executive functions, and disinhibition in aggressive, adolescent males. Neuropsychopharmacology. 1998 Oct;19(4):333–41.

428. DeNapoli J, Dodman N, Shuster L, Rand W, Gross K. Effect of dietary protein content and tryptophan supplementation on dominance aggression, territorial aggression, and hyperactivity in dogs. J Am Vet Med Assoc [Internet]. 2000;217(4):504–8. Available from: http://www.ncbi.nlm.nih.gov/pubmed/10953712

429. Chamberlain B, Ervin F, Pihl R, Young S. The effect of raising or lowering tryptophan levels on aggression in vervet monkeys. Pharmacol Biochem Behav [Internet]. 1987;28(4):503–10. Available from: http://www.ncbi.nlm.nih.gov/pubmed/3432316

430. Cleare AJ, Bond AJ. The effect of tryptophan depletion and enhancement on subjective and behavioural aggression in normal male subjects. Psychopharmacology (Berl) [Internet]. 1995;118(1):72–81. Available from: http://www.ncbi.nlm.nih.gov/pubmed/7597125

431. Peremans K, Audenaert K, Hoybergs Y, Otte A, Goethals I, Gielen I, et al. The effect of citalopram hydrobromide on 5-HT2A receptors in the impulsive-aggressive dog, as measured with 123I-5-I-R91150 SPECT. Eur J Nucl Med Mol Imaging [Internet]. 2005 Jun [cited 2013 Feb 19];32(6):708–16. Available from: http://www.ncbi.nlm.nih.gov/pubmed/15739093

432. Audero E, Mlinar B, Baccini G, Skachokova Z, Corradetti R, Gross C. Suppression of serotonin neuron firing increases aggression in mice. J Neurosci Res [Internet]. 2013;33(20):8678–88. Available from: http://www.ncbi.nlm.nih.gov/pubmed/23678112

433. Li N, Ghia J-E, Wang H, McClemens J, Cote F, Suehiro Y, et al. Serotonin Activates Dendritic Cell Function in the Context of Gut Inflammation. Am J Pathol [Internet]. 2011;178(2):662–71. Available from: http://www.ncbi.nlm.nih.gov/pmc/articles/PMC3069907/pdf/main.pdf

434. Ghia J, Li N, Wang H, Collins M, Deng Y, El-Sharkawy R, et al. Serotonin has a key role in pathogenesis of experimental colitis. Gastroenterology [Internet]. 2009;137(5):1649–60. Available from: http://www.ncbi.nlm.nih.gov/pubmed/19706294

435. Regmi SC, Kang Y, Park S, Park S-Y, Kim J-A. Role of serotonin in the pathogenesis of inflammatory bowel disease. FASEB J [Internet]. 2012;26(1108). Available from: http://www.fasebj.org/cgi/content/meeting_abstract/26/1_MeetingAbstracts/1108.4?sid=22955876-4a4c-48b3-a65f-c7981e0b7a45

436. Duerschmied D, Suidan GL, Demers M, Herr N, Carbo C, Brill A, et al. Platelet serotonin promotes the recruitment of neutrophils to sites of acute inflammation in mice. Blood [Internet]. 2012; Available from: http://bloodjournal.hematologylibrary.org/content/early/2012/12/11/blood-2012-06-437392.abstract

437. Bischoff SC, Mailer R, Pabst O, Weier G, Sedlik W, Li Z, et al. Role of serotonin in intestinal inflammation: knockout of serotonin reuptake transporter exacerbates 2,4,6-trinitrobenzene sulfonic acid colitis in mice. Am J Physiol Gastrointest Liver Physiol [Internet]. 2009 Mar [cited 2015 May 19];296(3):G685-95. Available from: http://www.ncbi.nlm.nih.gov/pubmed/19095763

438. Brown M, Bing C, King P, Pickavance L, Heal D, Wilding J. Sibutramine reduces feeding, body fat and improves insulin resistance in dietary-obese male Wistar rats independently of hypothalamic neuropeptide Y. Br J Pharmacol [Internet]. 2001;132(8):1898–904. Available from: http://www.ncbi.nlm.nih.gov/pmc/articles/PMC1572745/pdf/132-0704030a.pdf/

439. Tecott LH, Sun linda M, Akana SF, Strack AM, Lowenstein DH, Dallman MF, et al. Eating disorder and epilepsy in mice lacking 5-HT2C serotonin receptors. Nature [Internet]. 1994;374:542–6. Available from: http://www.nature.com/nature/journal/v374/n6522/abs/374542a0.html

440. Heisler L, Chu H, Tecott L. Epilepsy and obesity in serotonin 5-HT2C receptor mutant mice. Ann N Y Acad Sci [Internet]. 1998;15(861):74–8. Available from: http://www.ncbi.nlm.nih.gov/pubmed/9928241

441. Nonogaki K, Strack AM, Dallman MF, Tecott LH. Leptin-independent hyperphagia and type 2 diabetes in mice with a mutated serotonin 5-HT2C receptor gene. Nat Med [Internet]. 1998;4(10):1152–6. Available from: http://www.ncbi.nlm.nih.gov/pubmed/9771748

442. Hrboticky N, Leiter LA, Anderson GH. Effects of L-tryptophan on short term food intake in lean men. Nutr Res [Internet]. 1985;5(6):595–607. Available from: http://www.sciencedirect.com/science/article/pii/S0271531785802402

443. Lorrain DS, Riolo J V, Matuszewich L, Hull EM. Lateral hypothalamic serotonin inhibits nucleus accumbens Dopamine : Implications for Sexual Satiety. J Neurosci [Internet]. 1999;19(17):7648–52. Available from: http://www.jneurosci.org/content/19/17/7648.full.pdf

444. Karlsson A. Insulin resistance and sympathetic function in high spinal cord injury. Spinal Cord [Internet]. 1999;37(7):494–500. Available from: http://www.ncbi.nlm.nih.gov/pubmed/10438116

445. Zheng J, DiLorenzo DJ, McLaughlin L, Roberts AT, Greenway LF. Stimulation of sympathetic innervation in the upper gastrointestinal tract as a treatment for obesity. Med Hypotheses [Internet]. 2009;72(6):706–10. Available from: http://www.ncbi.nlm.nih.gov/pubmed/19246162

446. Camilleri M, Toouli J, Herrera M, Kulseng B, Kow L, Pantoja J, et al. Intra-abdominal vagal blocking (VBLOC therapy): clinical results with a new implantable medical device. Surgery [Internet]. 2008;143(6):723–31. Available from: http://www.ncbi.nlm.nih.gov/pubmed/18549888/

447. Lamey P, Savage A, Fisher B, Bloom S, Frier B. Secretion of epidermal growth factor in parotid saliva in diabetic patients: role of autonomic innervation. J Oral Pathol Med [Internet]. 1990;19(8):351–4. Available from: http://www.ncbi.nlm.nih.gov/pubmed/2250225

448. Wardlaw S. Regulation of beta-endorphin, corticotropin-like intermediate lobe peptide, and alpha-melanotropin-stimulating hormone in the hypothalamus by testosterone. Endocrinology [Internet]. 1986;119(1):19–24. Available from: http://www.ncbi.nlm.nih.gov/pubmed/3013585

449. Roberts ML. Testosterone-induced accumulation of epidermal growth factor in the submandibular salivary glands of mice, assessed by radioimmunoassay. Biochem Pharmacol [Internet]. 1974;23(23):3305–8. Available from: http://www.sciencedirect.com/science/article/pii/0006295274906546

450. Kovacheva EL, Hikim APS, Shen R, Sinha I, Sinha-Hikim I. Testosterone supplementation reverses sarcopenia in aging through regulation of myostatin, c-Jun NH2-terminal kinase, Notch, and Akt signaling pathways. Endocrinology [Internet]. 2010 Feb [cited 2013 Feb 16];151(2):628–38. Available from: http://www.pubmedcentral.nih.gov/articlerender.fcgi?artid=2817626&tool=pmcentrez&rendertype=abstract

451. Bhasin S, Storer TW, Berman N, Yarasheski KE, Clevenger B, Phillips J, et al. Testosterone replacement increases fat-free mass and muscle size in hypogonadal men. J Clin Endocrinol Metab [Internet]. 1997;82(2):407–13. Available from: http://www.ncbi.nlm.nih.gov/pubmed/9024227

452. Wang C, Swerdloff RS, Iranmanesh ALI, Dobs A, Snyder PJ, Cunningham G, et al. Transdermal Testosterone Gel Improves Sexual Function, Mood, Muscle Strength, and Body Composition Parameters in Hypogonadal Men. J Clin Endocrinol Metab [Internet]. 2000;85(8):2839–53. Available from: http://jcem.endojournals.org/content/85/8/2839.full.pdf

453. Mårin P, Holmäng S, Jönsson L, Sjöström L, Kvist H, Holm G, et al. The effects of testosterone treatment on body composition and metabolism in middle-aged obese men. Int J Obes Relat Metab Disord [Internet]. 1992;16(12):991–7. Available from: http://www.ncbi.nlm.nih.gov/pubmed/1335979

454. Rebuffé-Scrive M, Mårin P, Björntorp P. Effect of testosterone on abdominal adipose tissue in men. Int J Obes [Internet]. 1991;15(11):791–5. Available from: http://www.ncbi.nlm.nih.gov/pubmed/1778664

455. Xu X, De Pergola G, Björntorp P. Testosterone increases lipolysis and the number of beta-adrenoceptors in male rat adipocytes. Endocrinology [Internet]. 1991;128(1):379–82. Available from: http://www.ncbi.nlm.nih.gov/pubmed/1846106

456. Haider A, Yassin A, Doros G, Saad F. Effects of long-term testosterone therapy on patients with “diabesity”: Results of observational studies of pooled analyses in obese hypogonadal men with Type 2 Diabetes. Int J Endocrinol [Internet]. 2014 Jan;2014:683515. Available from: http://www.pubmedcentral.nih.gov/articlerender.fcgi?artid=3967627&tool=pmcentrez&rendertype=abstract

457. Sattler F, He J, Chukwuneke J, Kim H, Stewart Y, Colletti P, et al. Testosterone Supplementation Improves Carbohydrate and Lipid Metabolism in Some Older Men with Abdominal Obesity. J Gerontol Geriatr Res [Internet]. 2014;3(3). Available from: http://www.ncbi.nlm.nih.gov/pubmed/25392748

458. Ahlbom E, Prins G, Ceccatelli S. Testosterone protects cerebellar granule cells from oxidative stress-induced cell death through a receptor mediated mechanism. Brain Res [Internet]. 2001;892(2):255–62. Available from: http://www.ncbi.nlm.nih.gov/pubmed/11172772

459. Rishpon-Meyerstein N, Kilbridge T, Simone J, Fried W. The Effect of Testosterone on Erythropoietin Levels in Anemic Patients. Blood [Internet]. 1968;31(4):453–60. Available from: http://bloodjournal.hematologylibrary.org/content/31/4/453.long

460. Allemand MC, Irving BA, Asmann YW, Klaus KA, Tatpati L, Charles C, et al. Effect of Testosterone on Insulin Stimulated IRS1 Ser Phosphorylation in Primary Rat Myotubes — A Potential Model for PCOS-Related Insulin Resistance. PLoS One [Internet]. 2009;4(1):e4274. Available from: http://www.ncbi.nlm.nih.gov/pubmed/19169352

461. Franck-Lissbrant I, Häggström S, Damber JE, Bergh A. Testosterone stimulates angiogenesis and vascular regrowth in the ventral prostate in castrated adult rats. Endocrinology [Internet]. 1998 Feb;139(2):451–6. Available from: http://www.ncbi.nlm.nih.gov/pubmed/9449610

462. Moore K, Yeh K, Naito T, Kelley V. TNF-alpha enhances colony-stimulating factor-1-induced macrophage accumulation in autoimmune renal disease. J Immunol [Internet]. 1996;157:427–32. Available from: http://www.ncbi.nlm.nih.gov/pubmed/8683148

463. Hotamisligil GS, Murray DL, Choy LN, Spiegelman BM. Tumor necrosis factor alpha inhibits signaling from the insulin receptor. Proc Natl Acad Sci U S A [Internet]. 1994 May 24;91(11):4854–8. Available from: http://www.pubmedcentral.nih.gov/articlerender.fcgi?artid=43887&tool=pmcentrez&rendertype=abstract

464. Chen Y, Wu H, Winnall WR, Loveland KL, Makanji Y, Phillips DJ, et al. Tumour necrosis factor-α stimulates human neutrophils to release preformed activin A. Immunol Cell Biol [Internet]. Nature Publishing Group; 2011 Nov [cited 2013 Jan 4];89(8):889–96. Available from: http://www.ncbi.nlm.nih.gov/pubmed/21445090

465. Wu H, Chen Y, Winnall WR, Phillips DJ, Hedger MP. Regulation of activin A release from murine bone marrow-derived neutrophil precursors by tumour necrosis factor-α and insulin. Cytokine [Internet]. Elsevier Ltd; 2013 Jan [cited 2013 Jan 8];61(1):199–204. Available from: http://www.ncbi.nlm.nih.gov/pubmed/23116663

466. Matsuno H, Yudoh K, Katayama R, Nakazawa F, Uzuki M, Sawai T, et al. The role of TNF-alpha in the pathogenesis of inflammation and joint destruction in rheumatoid arthritis ( RA ): a study using a human RA / SCID mouse chimera. Rheumatology [Internet]. 2002;41:329–37. Available from: http://rheumatology.oxfordjournals.org/content/41/3/329.full.pdf

467. LaPensee CR, Hugo ER, Ben-Jonathan N. Insulin stimulates interleukin-6 expression and release in LS14 human adipocytes through multiple signaling pathways. Endocrinology [Internet]. 2008 Nov [cited 2013 Apr 23];149(11):5415–22. Available from: http://www.pubmedcentral.nih.gov/articlerender.fcgi?artid=2584585&tool=pmcentrez&rendertype=abstract

468. Finck BN, Johnson RW. Tumor necrosis factor ( TNF )-α induces leptin production through the p55 TNF receptor. Am J Physiol - Regul Integr Comp Physiol [Internet]. 2000;278(2):R537–43. Available from: http://ajpregu.physiology.org/content/278/2/R537.full.pdf

469. Zumbach MS, Boehme MWJ, Wahl P, Stremmel W, Ziegler R, Nawroth PP. Tumor Necrosis Factor Increases Serum Leptin Levels in Humans. J Clin Endocrinol Metab [Internet]. 1997;82(11):4080–2. Available from: http://jcem.endojournals.org/content/82/12/4080.full.pdf

470. Finck BN, Johnson RW. Anti-inflammatory agents inhibit the induction of leptin by tumor necrosis factor-alpha. Am J Physiol Regul Integr Comp Physiol [Internet]. 2002 May [cited 2013 Feb 9];282(5):R1429-35. Available from: http://www.ncbi.nlm.nih.gov/pubmed/11959686

471. Díaz-Delfín J, Hondares E, Iglesias R, Giralt M, Caelles C, Villarroya F. TNF-α represses β-Klotho expression and impairs FGF21 action in adipose cells: involvement of JNK1 in the FGF21 pathway. Endocrinology [Internet]. 2012;153(9):4238–45. Available from: http://www.ncbi.nlm.nih.gov/pubmed/22778214

472. Marsden P, Brenner B. Transcriptional regulation of the endothelin-1 gene by TNF-alpha. Am J Physiol [Internet]. 1992;262(4.1):C854-61. Available from: http://www.ncbi.nlm.nih.gov/pubmed/1566813

473. Elenkov I, Kovács K, Duda E, Stark E, Vizi E. Presynaptic inhibitory effect of TNF-alpha on the release of noradrenaline in isolated median eminence. Presynaptic Inhib Eff TNF-alpha release noradrenaline Isol Median eminenceJournal Neuroimmunol [Internet]. 1992;41(1):117–20. Available from: http://www.ncbi.nlm.nih.gov/pubmed/1460089

474. Laplante M, Charbonneau A, Avramoglu R, Pelletier P, Fang X, Bachelard H, et al. Distinct metabolic and vascular effects of dietary triglycerides and cholesterol in atherosclerotic and diabetic mouse models. Am J Physiol Endocrinol Metab [Internet]. 2013; Available from: http://www.ncbi.nlm.nih.gov/pubmed/23820620

475. Rattigan S, Clark MG, Barrett EJ. Acute Vasoconstriction-Induced Insulin Resistance in Rat Muscle In Vivo. Diabetes [Internet]. 1999 Mar;48(3):564–9. Available from: http://www.ncbi.nlm.nih.gov/pubmed/10078557

476. Borissova A, Tankova T, Kirilov G, Dakovska L, Kovacheva R. The effect of vitamin D3 on insulin secretion and peripheral insulin sensitivity in type 2 diabetic patients. Int J Clin Pract [Internet]. 2003;57(4):258–61. Available from: http://www.ncbi.nlm.nih.gov/pubmed/12800453

477. Cade C, Norman AW. Vitamin D3 improves impaired glucose tolerance and insulin secretion in the vitamin D-deficient rat in vivo. Endocrinology [Internet]. 1986;119(1):84–90. Available from: http://www.ncbi.nlm.nih.gov/pubmed/3013599

478. Holmlund-Suila E, Viljakainen H, Hytinantti T, Lamberg-Allardt C, Andersson S, Mäkitie O. High-dose vitamin d intervention in infants--effects on vitamin d status, calcium homeostasis, and bone strength. J Clin Endocrinol Metab [Internet]. 2012;97(11):4139–47. Available from: http://www.ncbi.nlm.nih.gov/pubmed/22933541

479. Kalueff A V, Keisala T, Minasyan A, Kuuslahti M, Miettinen S, Tuohimaa P. Behavioural anomalies in mice evoked by “‘ Tokyo ’” disruption of the Vitamin D receptor gene. Neurosci Res [Internet]. 2006;54(4):254–60. Available from: http://www.sciencedirect.com/science/article/pii/S0168010205003275

480. Norman A, Frankel J, Heldt A, Grodsky G. Vitamin D deficiency inhibits pancreatic secretion of insulin. Science (80- ) [Internet]. 1980;209(4458):823–5. Available from: http://www.ncbi.nlm.nih.gov/pubmed/6250216

481. Hosseinzadeh H, Moallem S, Moshiri M, Sarnavazi M, Etemad L. Anti-nociceptive and anti-inflammatory effects of cyanocobalamin (vitamin B12) against acute and chronic pain and inflammation in mice. Arzneimittelforschung [Internet]. 2012;62(7):324–9. Available from: http://www.ncbi.nlm.nih.gov/pubmed/22588629

482. Zachwieja JJ, Hendry SL, Smith SR, Harris RBS. Voluntary wheel running decreases adipose tissue mass and expression of leptin inRNA in Osborne-Mendel rats. Diabetes [Internet]. 1997;46(7):1159–66. Available from: http://www.ncbi.nlm.nih.gov/pubmed/9200651

483. Steensberg A, van Hall G, Osada T, Sacchetti M, Saltin B, Klarlund Pedersen B. Production of interleukin-6 in contracting human skeletal muscles can account for the exercise-induced increase in plasma interleukin-6. J Physiol [Internet]. 2000;529(1):237–42. Available from: http://www.ncbi.nlm.nih.gov/pubmed/11080265

484. Bulotta a., Hui H, Anastasi E, Bertolotto C, Boros LG, Di Mario U, et al. Cultured pancreatic ductal cells undergo cell cycle re-distribution and β-cell-like differentiation in response to glucagon-like peptide-1. J Mol Endocrinol [Internet]. 2002;29(3):347–60. Available from: http://www.ncbi.nlm.nih.gov/pubmed/12459036

485. Larsson H, Holst J, Ahrén B. Glucagon-like peptide-1 reduces hepatic glucose production indirectly through insulin and glucagon in humans. Acta Physiol Scand [Internet]. 1997;160(4):413–22. Available from: http://www.ncbi.nlm.nih.gov/pubmed/9338524

486. Farilla L, Bulotta A, Hirshberg B, Li Calzi S, Khoury N, Noushmehr H, et al. Glucagon-Like Peptide 1 Inhibits Cell Apoptosis and Improves Glucose Responsiveness of Freshly Isolated Human Islets. Endocrinology [Internet]. 2003;144(12):5149–58. Available from: http://www.ncbi.nlm.nih.gov/pubmed/12960095

487. Heer J De, Rasmussen C, Coy DH, Holst JJ. Glucagon-like peptide-1 , but not glucose-dependent insulinotropic peptide , inhibits glucagon secretion via somatostatin (receptor subtype 2 ) in the perfused rat pancreas. Diabetologia [Internet]. 2008;51(12):2263–70. Available from: http://www.ncbi.nlm.nih.gov/pubmed/18795252

488. Zander M, Madsbad S, Madsen JL, Holst JJ. Effect of 6-week course of glucagon-like peptide 1 on glycaemic control , insulin sensitivity , and beta-cell function in type 2 diabetes : a parallel-group study. Lancet [Internet]. 2002;359:824–30. Available from: http://www.ncbi.nlm.nih.gov/pubmed/11897280

489. Lepsen E, Lundgren J, Hartmann B, Pedersen O, Hansen T, Jørgensen N, et al. GLP-1 receptor agonist treatment increases bone formation and prevents bone loss in weight-reduced obese women. J Clin Endocrinol Metab [Internet]. 2015; Available from: http://www.ncbi.nlm.nih.gov/pubmed/26043228

490. Ma X, Meng J, Jia M, Bi L, Zhou Y, Wang Y, et al. Exendin-4, a glucagon-like peptide-1 receptor agonist, prevents osteopenia by promoting bone formation and suppressing bone resorption in aged ovariectomized rats. J Bone Miner Res [Internet]. 2013;28(7):1641–52. Available from: http://www.ncbi.nlm.nih.gov/pubmed/23427056

491. Greisen J, Juhl CB, Grøfte T, Vilstrup H, Jensen TS, Schmitz O. Acute pain induces insulin resistance in humans. Anesthesiology [Internet]. 2001;95(3):578–84. Available from: http://anesthesiology.pubs.asahq.org/article.aspx?articleid=1944846#81345070
