# Supplementary material for: Bi-stability in type 2 diabetes mellitus multi-organ signalling network
Source: PLoS One. 2017 Aug 2;12(8):e0181536. doi: 10.1371/journal.pone.0181536 (PMC5540287; doi:10.1371/journal.pone.0181536)
Supplement: S3 Table — (DOCX) [file pone.0181536.s005.docx]

**Supporting information**

**Supporting table**

**S3 Table. List of deletions of the 10% of the nodes that led to uni-stability and complete loss of stability.**

| **Simulation No.** | **Uni-stability** | | | | | | |
| --- | --- | --- | --- | --- | --- | --- | --- |
| **1** | ktg | fty | and | gmo | bdn | glp | adp |
| **2** | klt | noc | fty | sys | lep | pgl | hst |
| **3** | bgl | lep | gst | ctk | ghr | fty | gmo |
| **4** | myo | ins | avp | agr | and | epo | lpa |
| **5** | et1 | mlt | igf | chl | inj | ost | tri |
| **6** | dop | gap | msh | lep | adp | bgl | agr |
| **7** | btc | fty | ctk | grh | lep | inj | otg |
| **8** | avp | btc | myo | oxy | dop | lpa | hst |
| **9** | cts | hst | ost | lep | fty | msl | exe |
| **10** | exe | hgh | msl | hst | and | oxy | chl |
| **11** | noc | avp | vd3 | fdi | msh | oxy | fty |
| **12** | chl | egf | il6 | fty | ngf | glp | cfn |
| **13** | oxy | sys | dip | btc | gst | fty | and |
| **14** | ang | fty | oxy | msl | gt1 | chl | hst |
| **15** | ser | ktg | mlt | exe | gt1 | otg | hst |
| **16** | otg | epo | lpa | car | chl | hst | ocl |
| **17** | agr | adp | ins | tnf | ost | nox | epo |
| **18** | aox | fdi | lep | glp | fty | and | ina |
| **19** | myo | ina | bgl | dop | nep | et1 | epo |
| **20** | fdi | klt | ctk | avp | inr | ina | gmo |
| **21** | igf | gap | ina | ffa | bgl | edp | myo |
| **22** | mlt | dip | ocl | dop | sfr | gst | ina |
|  | | | | | | | |
| **Simulation No.** | **Complete loss of stability** | | | | | | |
| **1** | otg | gng | chl | tet | sys | pgl | gab |
| **2** | fty | et1 | dip | ser | agr | exe | igf |
| **3** | agr | bar | fdi | vd3 | gap | oxy | msl |
| **4** | fdi | ocl | crh | and | ina | msl | ang |
| **5** | dop | v12 | ghr | noc | ngf | ins | fty |
| **6** | tet | inj | aox | gap | vdl | dop | otg |
| **7** | vdl | dop | ocl | glp | agr | ser | gap |
| **8** | ata | chl | fty | inj | pgl | ina | bar |
| **9** | ina | mlt | oxy | bar | ocl | ata | dop |
| **10** | vd3 | chl | ang | hst | fty | cck | inr |
| **11** | mlt | agr | nep | et1 | aox | gmo | myo |

S3 Table footnotes. 7 nodes (10% of the nodes) were randomly deleted from each simulation and the model was run. The above mentioned sets of nodes are those which when deleted, led to uni-stability (1 similar state for all the nodes) or led to complete loss of stability.
