# Supplementary material for: Bi-stability in type 2 diabetes mellitus multi-organ signalling network
Source: PLoS One. 2017 Aug 2;12(8):e0181536. doi: 10.1371/journal.pone.0181536 (PMC5540287; doi:10.1371/journal.pone.0181536)
Supplement: S1 Text — (DOCX) [file pone.0181536.s006.docx]

**Supporting Information**

**Supporting Text**

**S1 Text. Merger and exclusion of links according to criteria defined in the text:** Components of inflammation like macrophage accumulation and reactive oxygen species were merged into one due to similar effects they show on the rest of the molecules in the network. Myokines were grouped under cytokines except for BDNF, SFRP5 and IL6. The latter three are branching points since they show some effects not shown by the other cytokines. The receptors of all the hormones included in the network were grouped along with their respective hormones expect for insulin and leptin. Insulin resistance and leptin resistance are observed in T2DM and they are phenomena that happen at the receptor level and not at the hormone production level. Hence, insulin action and leptin action are nodes and require a discrete place in the network. Gonadotropin-releasing hormone is responsible for the release of Leutinizing hormone and Follicle stimulating hormone and hence they were grouped under the former. Growth factors were grouped together with the exceptions of Nerve growth factor, Insulin-like growth factor 1 and Epidermal growth factor who deemed a different node. GABA in pancreas and in CNS has different effects on the other nodes in the network. But GABA in the pancreas has no upstream signal (from within the network). Hence, it was excluded.
